# Enantioselective synthesis of spiro[indoline-3,1′-pyrazolo[1,2-*b*]phthalazine] derivatives *via* an organocatalytic three-component cascade reaction†

**DOI:** 10.1039/d5ra01633a

**Published:** 2025-05-21

**Authors:** Luying Song, Yuhong Sun, Renbo An, Liming Wang, Ying Jin

**Affiliations:** a Department of Pharmacy, Jilin Medical University Jilin Jilin 132013 China jinying2288@163.com 13630635312@163.com; b College of Pharmacy, Yanbian University Yanji Jilin 133000 China

## Abstract

An asymmetric synthesis of spiro[indoline-3,1′-pyrazolo[1,2-*b*]phthalazine] derivatives was first developed through an organocatalysed cascade Knoevenagel/Michael/cyclization reaction using a quinidine-derived squaramide. Under the optimized conditions, the three-component reaction of isatins, malononitrile (cyanoacetate) and phthalhydrazide yields the desired pyrazolophthalazinyl spirooxindoles in good yields (73–90%) with up to >99% enantiomer excess (ee).

## Abstract 2

A series of chiral spiro[indoline-3,1′-pyrazolo[1,2-*b*]phthalazine] derivatives with excellent enantioselectivity were obtained for the first time through an organocatalysed cascade reaction.

## Graphical abstract

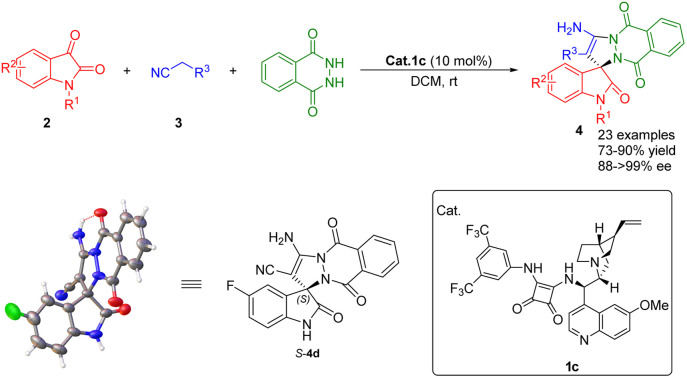

## Introduction

**Fig. 1:**
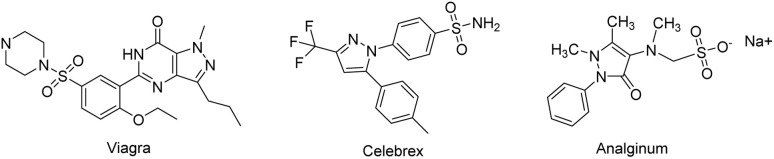
Examples of biologically active drugs containing pyrazole ring.

The pyrazole ring is a key moiety in numerous biologically active compounds and clinical drugs,^1^ such as Viagra, Celebrex, and Analginum (Fig. 1). Therefore, the synthesis of heterocyclic compounds containing the pyrazole ring is a research hotspot in the fields of organic synthesis and medicinal chemistry.

On the other hand, heterocyclic fused phthalazines have been found to possess cytotoxic, antimicrobial, anticonvulsant, antifungal, anticancer, anti-inflammatory, and cardiotonic activities.^2^ They also exhibited potential as new luminescence materials or fluorescence probes.^3^

In addition, optically active spirooxindole derivatives have been extensively investigated because of their prominent activities and wide utility as synthetic intermediates for alkaloids and clinical drugs.^4^ The methods for synthesizing various spirooxindoles, including spiro[N-heterocycle-oxindoles], spiro[cycloalkane-oxindoles] and spiro[O-heterocycle-oxindoles] *etc.* have been well established.^5*a*^ Over the past few years, significant progress has been made in the stereoselective construction of spirooxindole.^5^

**Scheme 1:**
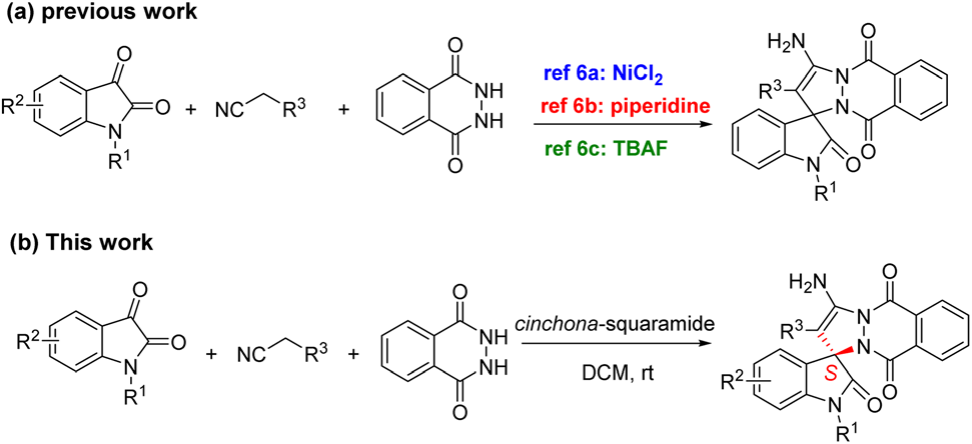
Spiro[indoline-3,1′-pyrazolo[1,2-*b*]phthalazine].

Based on the biological activity of pyrazole, phthalazine, and spiroindole skeleton, according to the splicing principle, chemists have considered combining these three parts into one molecule. At present, several methods have been developed for the synthesis of racemic spiro[indoline-3,1′-pyrazolo[1,2-*b*]phthalazine] derivatives.^6^ Zhang,^6*a*^ Shi^6*b*^ and Kefayati^6*c*^ groups reported one-pot three-component reaction catalyzed by nickel chloride, pyridine, or tetrabutylammonium fluoride (TBAF) respectively as shown in Scheme 1(a). However, there are no reports on the synthesis of optically pure pyrazolophthalazinyl spirooxindoles.

**Fig. 2:**
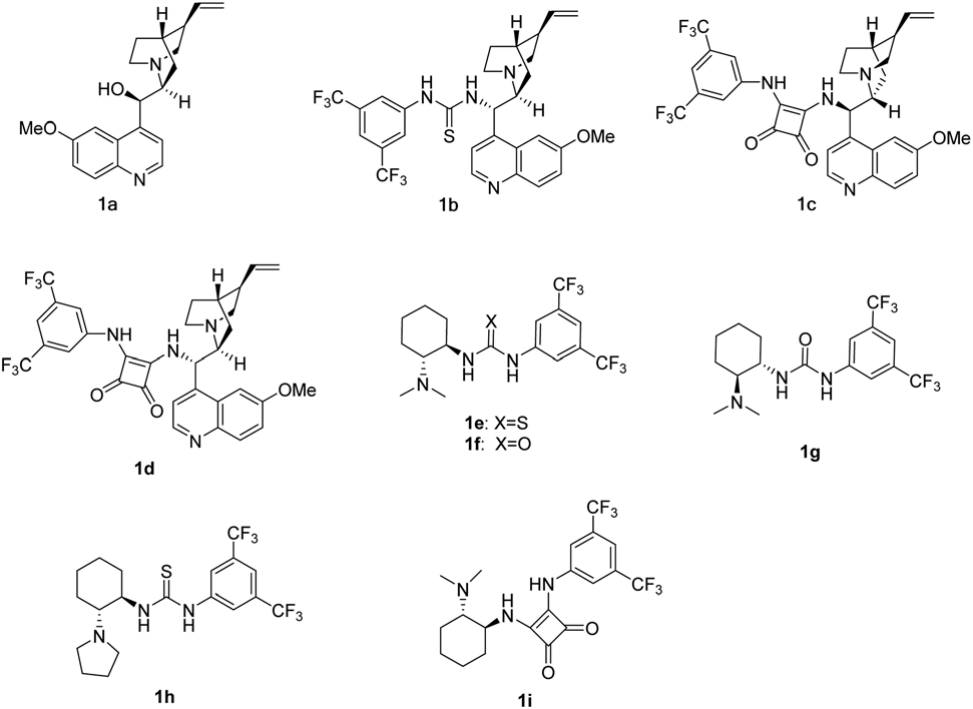
The structure of catalysts 1a–1i.

As well known, the development of synthetic methods to access optically active spiro-heterocycles would be of significance for drug discovery.^7^ So, we herein first reported the asymmetric Michael/cyclization cascade reaction of malononitrile (cyanoacetate), phthalhydrazide with a variety of the substituted isatins (N1, C4, C5, C6, C7 substituents) to construct spiro-heterocycles by using cinchona-squaramide catalyst (Scheme 1(b)). Bifunctional organocatalysts 1a–1i including cinchona alkaloids and Takemoto's catalysts bearing (thio)urea or squaramide^8^ have been applied in this cascade reaction as shown in Fig. 2.

## Results and discussion

We initially screened the bifunctional catalysts in the Knoevenagel/Michael/cyclization reaction of isatin (2a), malononitrile (3a) and phthalhydrazide with DCM as a solvent in the presence of 10 mol% of catalysts 1a–1i (Table 1).

**Table 1:** Asymmetric cascade reaction of isatin, malononitrile and phthalhydrazide catalyzed by 1a–i^a^

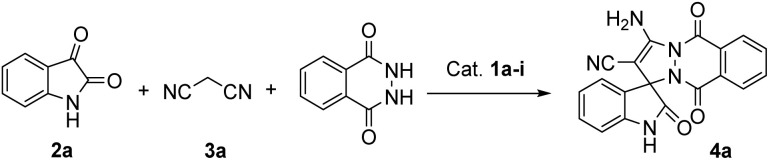					
Entry	Cat.	Solvent	Cat. loading (mol%)	Yield^b^ (%)	%ee^c^^,^^d^
1	1a	DCM	10	75	6 (−)
2	1b	DCM	10	83	92 (+)
3	1c	DCM	10	88	98 (−)
4	1d	DCM	10	82	94 (+)
5	1e	DCM	10	76	79 (+)
6	1f	DCM	10	80	97 (+)
7	1g	DCM	10	78	94 (−)
8	1h	DCM	10	81	89 (−)
9	1i	DCM	10	82	96 (+)
10	1c	CHCl_3_	10	83	93
11	1c	DCE	10	84	95
12	1c	CH_3_CN	10	70	85
13	1c	EA	10	68	51
14	1c	EtOH	10	65	39
15	1c	Et_2_O	10	—	—
16	1c	DCM	5	70	79

a Reaction conditions: isatin 2a (0.1 mmol), malononitrile 3a (0.1 mmol), phthalhydrazide (0.1 mmol) and catalysts 1a–1i in solvent (1.0 mL) at rt for 24 h.

b Isolated yield.

c Determined by HPLC analysis (Chiralpak OJ–H).

d The sign in parentheses indicates the sign of the optical rotation.

As shown in Table 1, catalysts 1a–i smoothly catalyzed the reaction to provide the desired product in 75–88% yields (entries 1–9). Among them, quinidine-derived squaramide catalyst 1c was screened to be the optimal catalyst in terms of yield and enantioselectivity (98% ee, entry 3). In addition, Takemoto's catalyst 1f with a urea moiety and catalyst 1i bearing a squaramide moiety induced the reaction with excellent enantioselectivities (entries 6 and 9). By comparison, catalysts 1e and 1h with a thiourea moiety showed bad asymmetric induction to give decreased ee values (79% and 89%, entries 5 and 8).

Since stereoselectivity of up to 98% has been achieved under the current conditions, there is not much room for further improvement. However, we would like to investigate the effect of solvents on the stereoselectivity of the reaction, and whether lowering the loading of catalyst can maintain excellent enantioselectivity. The results showed that solvents have a significant impact on the catalytic performance of the catalyst. Among the 8 selected solvents, dichloromethane was screened as the optimal solvent for the yield and enantioselectivity (entry 3), and halogenated alkane as solvents were found to be suitable for this reaction to give 93% and 95% ees respectively (entries 10 and 11), while low enantioselectivities were obtained in ethyl acetate and EtOH (entries 13 and 14). Surprisingly, Et_2_O was used as solvent, the transformation did not occur even with prolonged the reaction time of 36 h (entry 15). Additionally, reducing the catalyst loading to 5 mol% led to an obvious decrease in enantioselectivity and yield (entry 16 *vs.* 3). Thus, the optimized conditions were determined to be DCM as the solvent with a 10 mol% loading of catalyst 1c at rt.

With the optimized conditions in hand, we explored the scope and general applicability of the protocol. A wide range of substituted isatins (N1, C4, C5, C6 and C7 substituents) and 2 types of electron-rich nucleophiles, including malononitrile and cyanoacetates, were evaluated as shown in Table 2.

**Table 2:** Scope of the enantioselective three-component Knoevenagel/Michael/cyclization^a^

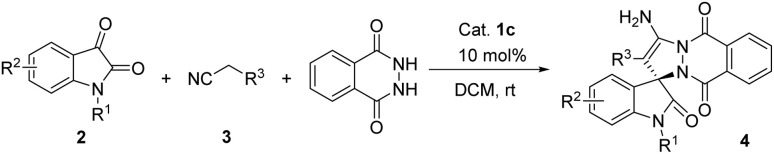					
Entry	2 (*R*^1^, *R*^2^)	*R* ^3^	Product	Yield^b^ (%)	%ee^c^
1	2a (H,H)	CN	4a	88	98
2	2b (H, 4-Cl)	CN	4b	80	96
3	2c (H, 4-Br)	CN	4c	87	92
4	2d (H, 5-F)	CN	4d	81	98
5	2e (H, 5-Cl)	CN	4e	82	94
6	2f (H, 5-Br)	CN	4f	83	98
7	2g (H, 5-NO_2_)	CN	4g	90	93
8	2h (H, 5-Me)	CN	4h	85	99
9	2i (H, 5-OMe)	CN	4i	79	96
10	2j (H, 6-Br)	CN	4j	80	97
11	2k (H, 7-F)	CN	4k	82	90
12	2l (H, 7-Cl)	CN	4l	78	91
13	2m (H, 7-Br)	CN	4m	81	98
14	2n (H, 7-Me)	CN	4n	80	95
15	2o (H, 7-NO_2_)	CN	4o	79	93
16	2p (Me, H)	CN	4p	83	>99
17	2q (Bn, H)	CN	4q	81	88
18	2r (Bn, 5-Cl)	CN	4r	82	92
19	2a (H, H)	COOMe	4s	73	91
20	2a (H, H)	COOEt	4t	78	98
21	2f (H, 5-Br)	COOEt	4u	80	99
22	2m (H, 7-Br)	COOEt	4v	81	99
23	2p (Me, H)	COOEt	4w	82	99

a Reaction conditions: isatins 2 (0.1 mmol), 3 (0.1 mmol), phthalhydrazide (0.1 mmol) and catalyst 1c (0.01 mmol) in DCM (1.0 mL) at rt for 24 h.

b Isolated yield.

c Determined by HPLC analysis (Chiralpak IA, AS, AD, Chiralcel OD, OJ).

All of the substrates reacted smoothly to give the corresponding products in 73–90% yield with excellent ees (88–>99%). Compared with *N*1-benzyl substituted isatin, no substitution or methyl substituted isatin as substrates were found to be better for the enantioselectivity (entries 1 and 16 *vs.* entry 17). To broaden the scope of substrates, cyanoacetates were applied instead of malononitrile in the reaction, and excellent ee values of 91–99% were obtained (entries 19–23). Notably, ethyl cyanoacetate as substrate was found to be better than methyl cyanoacetate for the enantioselectivity and yield of the reaction (entry 20 *vs.* entry 19). Therefore, the screened catalytic condition showed excellent general applicability for this three components cascade reaction.

**Fig. 3:**
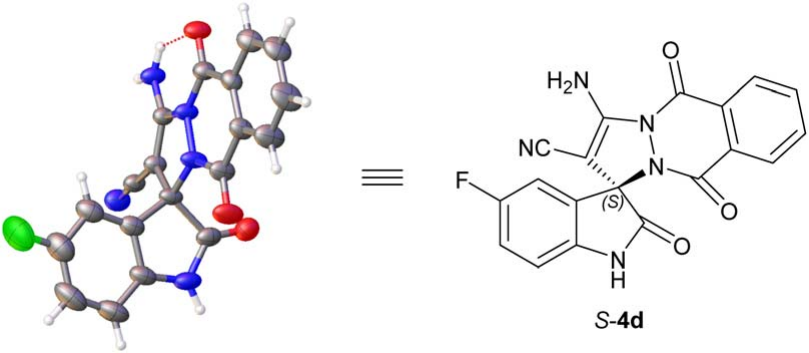
X-ray crystal structure of *S*-4d (CCDC: 2384066).^9^

The absolute configuration of spiro[indoline-3,10-pyrazolo[1,2-*b*]phthalazine] products 4 were unambiguously assigned as *S* according to the X-ray crystal structure analysis of 4d (Fig. 3).

**Scheme 2:**
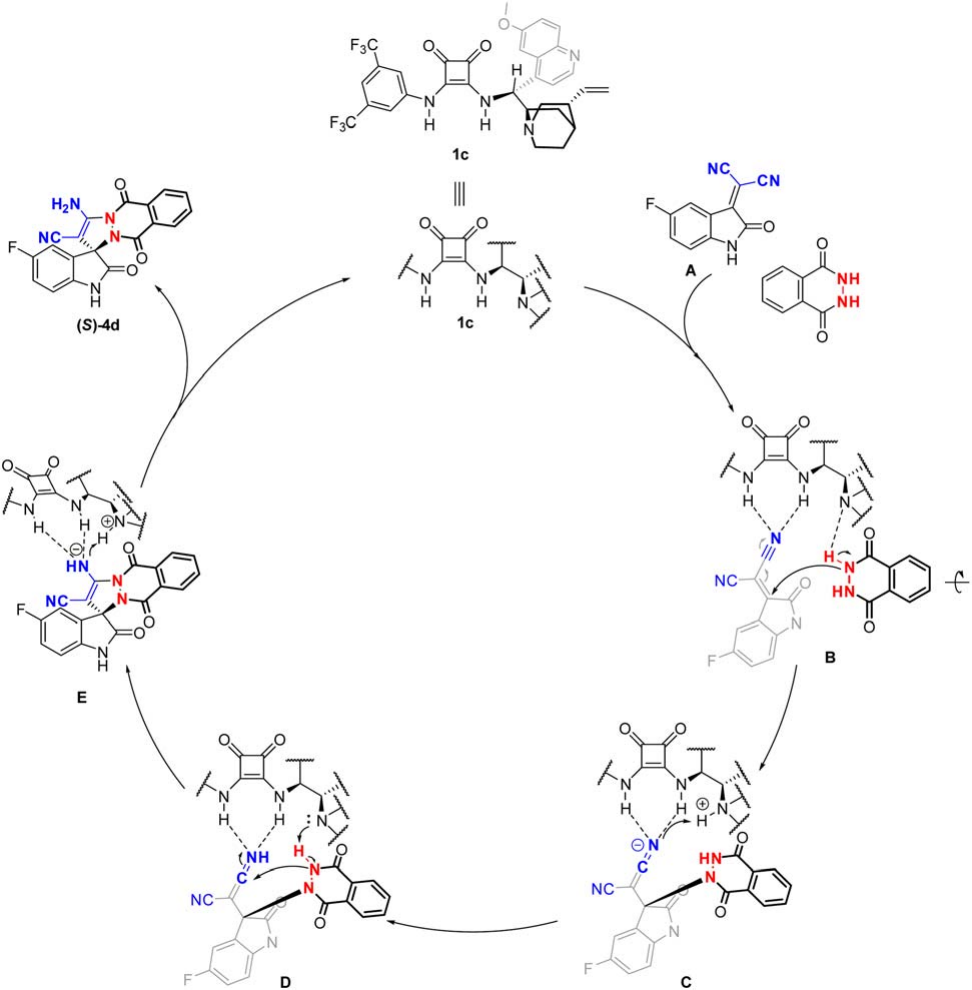
Proposed mechanism for the formation of *S*-4d.

On the basis of the absolute stereochemistry of 4d, a plausible mechanism for the formation of spirooxindole derivative 4d was proposed according to the literature (Scheme 2).^10^ Isatin (2a) first condenses with malononitrile to yield the isatylidene malononitrile (A), one cyanide group of which is fixed and activated by the neighboring two squaramide N–H moieties through double H-bonding, while phthalhydrazide is activated by an interaction between the tertiaryamine moiety of 1c and the amino group of phthalhydrazide (B). Then, *Si*-face addition of the nucleophilic phthalhydrazide to the electron-deficient isatylidene malononitrile results in a Michael adduct intermediate (C), which in turn transfers to intermediate (D) through proton immigration. Next, the intramolecular nucleophilic addition of the amino group to the cyano group produces a cyclic imino carbanion (E), which is further transformed to the final (*S*)-4d through subsequent proton immigration and tautomerization (Scheme 2).

## Conclusions

In summary, we described the first enantioselective Knoevenagel/Michael/cyclization cascade reaction of isatins, malononitrile (or cyanoacetates) and phthalhydrazide to produce spiro[indoline-3,10-pyrazolo[1,2-*b*]phthalazine] in high enantioselectivity (88–>99% ees). Moreover, we used our optimized conditions to expand upon the substrate scope of this reaction.

## Data availability

The data supporting this article have been included as part of the ESI.†

## Author contributions

L. Y. Song and Y. H. Sun are co-first authors; they contributed equally to this work. They performed the experiments, acquired the original data and cultivated the single crystal. R. B. An conducted instrumental analysis. L. M. Wang supervised the experiment, analyzed and checked all the data, provided funding supporting. Y. Jin designed the research plan, wrote the draft and revised manuscript. All authors have given approval to the final version of the manuscript.

## Conflicts of interest

There are no conflicts to declare.

## Supplementary Material

RA-015-D5RA01633A-s001

RA-015-D5RA01633A-s002
