# Supplementary material for: Enantioselective synthesis of spiro[indoline-3,1′-pyrazolo[1,2-b]phthalazine] derivatives via an organocatalytic three-component cascade reaction
Source: RSC Adv. 2025 May 21;15(21):17031–5. doi: 10.1039/d5ra01633a (PMC12094013; doi:10.1039/d5ra01633a)
Supplement: RA-015-D5RA01633A-s001 [file RA-015-D5RA01633A-s001.pdf]

Supplementary Information (SI) for RSC Advances.
This journal is © The Royal Society of Chemistry 2025

Supporting Information

Enantioselective Synthesis of Spiro [indoline-3,1′-pyrazolo [1,2-*b*] phthalazine] Derivatives *via* Organocatalytic Three-Component Cascade Reaction

Luying Song, [†a,b] Yuhong Sun, [†a,b] Renbo An, [b] Liming Wang,*[a] Ying Jin*[a,b]

[a] Department of Pharmacy, Jilin Medical University, Jilin, Jilin 132013, China
[b] Department of Pharmacy, Yanbian University, Yanji, Jilin 133000, China
*E-mail: jinying2288@163.com; 13630635312@163.com

†These authors contributed equally to this work.

Table of Contents

1. General Information S1
2. Experimental Procedure S1
3. ^{1}H NMR and ^{13}C NMR Spectra S9
4. HPLC Traces S25
5. X-Ray Crystal Data of Compound (*S*)-**4d** S48

1. General Information

^1H NMR and ^{13}C NMR spectra were recorded respectively on a Bruker spectrometer at 500/400 MHz and 125/100 MHz, using DMSO-d_6 as a solvent. The chemical shifts were reported in ppm, and the residual nondeuterated solvent as internal standard (2.5 and 39.5 ppm, respectively). High resolution mass spectra (HRMS) were measured on a triple TOF 5600+ mass spectrometer equipped with an electrospray ionization (ESI†) source in the positive-ion mode. The enantiomeric excess (ee) values of the products were determined by chiral HPLC, using Daicel Chiralpak IA-H, AS-H, AD-H, and Daicel Chiralcel OD-H, OJ-H columns (4.6 mm, 250 mm). The reactions were monitored by thin layer chromatography (TLC). Purifications by column chromatography were conducted over silica gel (200–300 mesh). The catalysts **1a–1i** were purchased from Daicel Chiral Technologies (China)

2. Experimental Procedures

General procedure for the asymmetric Knoevenagel/Michael/cyclization reaction of isatins, malononitrile or cyanoacetates and phthalhydrazide

To a solution of isatins **2** (0. 10 mmol), malononitrile or cyanoacetates **3** (0.10 mmol), and phthalhydrazide (0.10 mmol) and **1c** (0.01 mmol), CH_2Cl_2 (1.0 mL) was added. The resulting mixture was stirred at room temperature for 24 hours (TLC). After the reaction was finished, the crude mixture was directly loaded onto a column packed with silica gel with hexane/EtOAc (2:1) as eluent to afford the 23 chiral compounds **4a-w**. Among of 23 products, there are 8 new compounds. The measured ^1H NMR data of known compounds were consistent with the corresponding data in the literature. [1]

(*S*)-3′-amino-2,5′,10′-trioxo-5′,10′-dihydrospiro[indoline-3,1′-pyrazolo[1,2-*b*]phthalazine]-2′-carbonitrile (4a) (known compound lit.[1a]): light yellow solid, m.p.: 270.8-271.8 °C; ^1H NMR (500 MHz, DMSO-d_6) δ 10.94 (s, 1H), 8.35-8.28 (m, 3H), 8.11 – 7.93 (m, 3H), 7.47 (d, J = 7.5 Hz, 1H), 7.31 (td, J = 7.5, 1.5 Hz, 1H), 7.01 (t, J = 7.5 Hz, 1H), 6.93 (d, J = 8.0 Hz, 1H); $[\alpha]_D^{25}$= -7.9 (c 0.45, MeOH)(98% ee); HPLC (Chiralcel OJ, hexane:^{i}PrOH = 70:30, 1.0 mL/min, 254 nm), t_R= 14.7 min (major),

24.1 min (minor).

(*S*)-3′-amino-4-chloro-2,5′,10′-trioxo-5′,10′-dihydrospiro[indoline-3,1′-pyrazolo[1,2-*b*]phthalazine]-2′-carbonitrile (**4b**) (known compound, lit.[1b]): light yellow solid, m.p.: 300.2-301.1 °C; ^{1}H NMR (500 MHz, DMSO-d_6) δ 11.27 (s, 1H), 8.47 (s, 2H), 8.36 – 8.27 (m, 1H), 8.15 – 7.99 (m, 3H), 7.37 (t, *J* = 8.0 Hz, 1H), 7.05 (d, *J* = 8.0 Hz, 1H), 6.96 (d, *J* = 8.0 Hz, 1H); $[\alpha]_D^{25}$= 91.7 (c 0.52, MeOH)(96% ee); HPLC (Chiralcel OD-H, hexane:^{i}PrOH = 80:20, 1.0 mL/min, 254 nm), t_R= 24.9 min (major), 36.4 min (minor).

(*S*)-3′-amino-4-bromo-2,5′,10′-trioxo-5′,10′-dihydrospiro[indoline-3,1′-pyrazolo[1,2-*b*]phthalazine]-2′-carbonitrile (**4c**) (known compound, lit.[1b]): light yellow solid, m.p.: 308.9-309.7 °C; ^{1}H NMR (500 MHz, DMSO-d_6) δ 11.25 (s, 1H), 8.47 (s, 2H), 8.39 – 8.27 (m, 1H), 8.19 – 7.95 (m, 3H), 7.29 (t, *J* = 8.0 Hz, 1H), 7.19 (dd, *J* = 8.0, 1.0 Hz, 1H), 6.98 (dd, *J* = 8.0, 1.0 Hz, 1H); $[\alpha]_D^{25}$= 92.7 (c 0.58, MeOH)(92% ee); HPLC (Chiralcel OD-H, hexane: ^{i}PrOH = 80:20, 1.0 mL/min, 254 nm), t_R= 25.9 min (major), 36.5 min (minor).

(*S*)-3′-amino-5-fluoro-2,5′,10′-trioxo-5′,10′-dihydrospiro[indoline-3,1′-pyrazolo[1,2-*b*]phthalazine]-2′-carbonitrile (**4d**) (known compound, lit.[1b]): light yellow green solid, m.p.: 257.0-258.8 °C; ^{1}H NMR (500 MHz, DMSO-d_6) δ 10.97 (s, 1H), 8.38 (s, 2H), 8.34 – 8.26 (m, 1H), 8.14 – 7.93 (m, 3H), 7.50 (dd, *J* = 8.0, 2.5 Hz, 1H), 7.15 (ddd, *J* = 9.5, 8.5, 2.5 Hz, 1H), 6.93 (dd, *J* = 8.5, 4.0 Hz, 1H); $[\alpha]_D^{25}$= -19.0 (c 0.49, MeOH)(98% ee); HPLC (Chiralpak AD-H, hexane:^{i}PrOH = 70:30, 1.0 mL/min, 254 nm), t_R= 18.3 min (major), 31.5 min (minor).

(*S*)-3′-amino-5-chloro-2,5′,10′-trioxo-5′,10′-dihydrospiro[indoline-3,1′-pyrazolo[1,2-*b*]phthalazine]-2′-carbonitrile (**4e**) (known compound, lit.[1a]): light yellow green solid, m.p.: 324.6-325.8 °C; ^{1}H NMR (500 MHz, DMSO-d_6) δ 11.09 (s, 1H), 8.39 (s, 2H), 8.35 – 8.24 (m, 1H), 8.14 – 7.94 (m, 3H), 7.70 (d, *J* = 2.0 Hz, 1H), 7.36 (dd, *J* = 8.5, 2.5 Hz, 1H), 6.95 (d, *J* = 8.5 Hz, 1H); $[\alpha]_D^{25}$= 47.5 (c 0.50, MeOH)(94% ee); HPLC (Chiralcel OJ, hexane:^{i}PrOH = 80:20, 1.0 mL/min, 254 nm), t_R= 18.6 min (major), 27.8 min (minor).

(*S*)-3′-amino-5-bromo-2,5′,10′-trioxo-5′,10′-dihydrospiro[indoline-3,1′-

pyrazolo[1,2-*b*]phthalazine]-2′-carbonitrile (**4f**) (known compound, lit.[1a]): light yellow green solid, m.p.: 349.9-350.7 °C; ^{1}H NMR (500 MHz, DMSO-d_6) δ 11.09 (s, 1H), 8.39 (s, 2H), 8.31 (dd, *J*= 8.5, 2.0 Hz, 1H), 8.16 – 7.92 (m, 3H), 7.82 (d, *J*= 2.0 Hz,1H),7.49 (dd, *J*= 8.5, 2.0 Hz, 1H), 6.90 (d, *J* = 8.5 Hz, 1H); $[\alpha]_D^{25}$= 48.2 (c 0.54, MeOH)(98% ee); HPLC (Chiralpak OJ, hexane:^{i}PrOH = 70:30, 1.0 mL/min, 254 nm), t_R= 11.7 min (major), 15.4 min (minor).

(*S*)-3′-amino-5-nitro-2,5′,10′-trioxo-5′,10′-dihydrospiro[indoline-3,1′-pyrazolo[1,2-*b*]phthalazine]-2′-carbonitrile (**4g**) (known compound, lit.[1a]): light yellow green solid, m.p.: 280.6-281.7 °C; ^{1}H NMR (500 MHz, DMSO-d_6) δ 11.68 (s, 1H), 8.63 (d, *J* = 2.5 Hz, 1H), 8.46 (s, 2H), 8.37 – 8.25 (m, 2H), 8.07 – 7.95 (m, 3H), 7.16 (d, *J* = 8.5 Hz, 1H); $[\alpha]_D^{25}$= 165.0 (c 0.57, MeOH)(93% ee); HPLC (Chiralpak AD, hexane:^{i}PrOH = 70:30, 1.0 mL/min, 254 nm), t_R= 18.6 min (major), 47.9 min (minor).

(*S*)-3′-amino-5-methyl-2,5′,10′-trioxo-5′,10′-dihydrospiro[indoline-3,1′-pyrazolo[1,2-*b*]phthalazine]-2′-carbonitrile (**4h**) (known compound, lit.[1a]): light yellow solid, m.p.: 277.2-278.1 °C; ^{1}H NMR (500 MHz, DMSO-d_6) δ 10.82 (s, 1H), 8.39 – 8.27 (m, 3H), 8.11 – 7.94 (m, 3H), 7.31 (d, *J* = 1.5 Hz, 1H), 7.11 (ddd, *J* = 8.0, 2.0, 1.0 Hz, 1H), 6.81 (d, *J* = 8.0 Hz, 1H), 2.21 (s, 3H); $[\alpha]_D^{25}$= 38.9(c 0.49, MeOH)(99% ee); HPLC (Chiralcel OJ, hexane:^{i}PrOH = 80:20, 1.0 mL/min, 254 nm), t_R= 18.8 min (major), 25.6 min (minor).

(*S*)-3′-amino-5-methoxy-2,5′,10′-trioxo-5′,10′-dihydrospiro[indoline-3,1′-pyrazolo[1,2-*b*]phthalazine]-2′-carbonitrile (**4i**) : light yellow solid, m.p.: 251.0-251.9 °C; ^{1}H NMR (500 MHz, DMSO-d_6) δ 10.74 (s, 1H), 8.43 – 8.25 (m, 3H), 8.09 – 7.96 (m, 3H), 7.20 (d, *J* = 2.5 Hz, 1H), 6.90 – 6.77 (m, 2H), 3.67 (s, 3H);^{13}C NMR (125 MHz, DMSO-d_6) δ 172.4, 156.4, 155.6, 152.5, 151.7, 135.3, 135.1, 134.4, 128.7, 127.9, 127.6, 127.0, 126.6, 115.4, 114.4, 111.1, 110.8, 70.2, 60.4, 55.5; HRMS (ESI) m/z: $[M+Na]^+$ calcd for $C_{20}H_{13}N_5O_4Na^+$: 410.0860, found 410.0866; $[\alpha]_D^{25}$= 38.8 (c 0.43, MeOH)(96% ee); HPLC (Chiralpak AS, hexane:^{i}PrOH = 80:20, 1.0 mL/min, 254 nm), t_R= 40.8 min (major), 47.1 min (minor).

(*S*)-3′-amino-6-bromo-2,5′,10′-trioxo-5′,10′-dihydrospiro[indoline-3,1′-

pyrazolo[1,2-*b*]phthalazine]-2′-carbonitrile (**4j**) (known compound, lit.[1b]): light yellow solid, m.p.: 257.9-258.6 °C; ^{1}H NMR (500 MHz, DMSO-d_6) δ 11.11 (s, 1H), 8.38 (s, 2H), 8.33 – 8.22 (m, 1H), 8.11 – 7.94 (m, 3H), 7.47 (d, *J* = 8.0 Hz, 1H), 7.22 (dd, *J* = 8.0, 2.0 Hz, 1H), 7.09 (d, *J* = 2.0 Hz, 1H); $[\alpha]_D^{25}$= -13.5 (c 0.35, MeOH)(97% ee); HPLC (Chiralcel OJ, hexane:^{i}PrOH = 80:20, 1.0 mL/min, 254 nm), t_R= 18.7 min (major), 32.2 min (minor).

(*S*)-3′-amino-7-fluoro-2,5′,10′-trioxo-5′,10′-dihydrospiro[indoline-3,1′-pyrazolo[1,2-*b*]phthalazine]-2′-carbonitrile (**4k**): light yellow solid, m.p.: 206.3-207.1 °C; ^{1}H NMR (500 MHz, DMSO-d_6) δ 11.52 (s, 1H), 8.40 (s, 2H), 8.35 – 8.27 (m, 1H), 8.12 – 7.94 (m, 3H), 7.38 (dd, *J* = 7.5, 1.0 Hz, 1H), 7.26 (ddd, *J* = 10.5, 8.5, 1.0 Hz, 1H), 7.05 (ddd, *J* = 8.5, 7.5, 4.5 Hz, 1H); ^{13}C NMR (125 MHz, DMSO-d_6) δ 172.4, 156.3, 152.7, 151.8, 147.5, 145.5, 135.1, 134.5, 129.2 (d, *J*=12.9 Hz), 128.7, 128.2 (d, *J*= 3.8 Hz),127.7 (d, *J*= 3.9 Hz), 127.0, 123.5(d, *J*= 5.5 Hz), 120.8, 117.5 (d, *J*= 17.0 Hz), 114.3, 69.7, 59.6; HRMS (ESI) m/z: $[M+Na]^+$ calcd for $C_{19}H_{10}FN_5O_3Na^+$ 398.0660, found 398.0665; $[\alpha]_D^{25}$= -16.3 (c 0.40, MeOH)(90% ee); HPLC (Chiralpak AS, hexane:^{i}PrOH = 80:20, 1.0 mL/min, 254 nm), t_R= 21.6 min (minor), 34.7 min (major).

(*S*)-3′-amino-7-chloro-2,5′,10′-trioxo-5′,10′-dihydrospiro[indoline-3,1′-pyrazolo[1,2-*b*]phthalazine]-2′-carbonitrile (**4l**): light yellow solid, m.p.: 275.7-276.6 °C; ^{1}H NMR (500 MHz, DMSO-d_6) δ 11.44 (s, 1H), 8.40 (s, 2H), 8.30 (dd, *J* = 2.0, 8.0 Hz, 1H), 8.13 – 7.90 (m, 3H), 7.50 (dd, *J* = 7.5, 1.0 Hz, 1H), 7.40 (dd, *J* = 8.0, 1.0 Hz, 1H), 7.05 (dd, *J* = 7.5, 8.0 Hz, 1H);^{13}C NMR (125 MHz, DMSO-d_6) δ 172.6, 156.3, 152.6, 151.9, 139.8, 135.1, 134.5, 130.5, 128.7, 127.7, 127.3, 127.0, 123.9, 123.4, 114.5, 114.3, 70.1, 59.5; HRMS (ESI) m/z: $[M+Na]^+$ calcd for $C_{19}H_{10}ClN_5O_3Na^+$ 414.0364, found 414.0370; $[\alpha]_D^{25}$= -122.5 (c 0.56, MeOH)(91% ee); HPLC (Chiralpak AS, hexane:^{i}PrOH = 70:30, 1.0 mL/min, 254 nm), t_R= 13.0 min (minor), 19.2 min (major).

(*S*)-3′-amino-7-bromo-2,5′,10′-trioxo-5′,10′-dihydrospiro[indoline-3,1′-pyrazolo[1,2-*b*]phthalazine]-2′-carbonitrile (**4m**): light yellow solid, m.p.: 204.1-205.0 °C; ^{1}H NMR (500 MHz, DMSO-d_6) δ 11.29 (s, 1H), 8.40 (s, 2H), 8.35 – 8.26

(m, 1H), 8.15 – 7.93 (m, 3H), 7.52 (ddd, *J* = 8.0, 4.5, 1.0 Hz, 2H), 6.99 (t, *J* = 8.0 Hz, 1H); ^{13}C NMR (125 MHz, DMSO-d_6) δ 172.6, 156.3, 152.6, 151.9, 141.5, 135.1, 134.5, 133.4, 128.7, 128.6, 127.7, 127.4, 127.0, 124.2, 123.9, 114.3, 102.7, 70.3, 59.6; HRMS (ESI) m/z: $[M+Na]^+$ calcd for $C_{19}H_{10}BrN_5O_3Na^+$ 457.9859, found 457.9855; $[\alpha]_D^{25}$= -253.3 (c 0.51, MeOH)(98% ee); HPLC (Chiralpak AS, hexane:^{i}PrOH = 70:30, 1.0 mL/min, 254 nm), t_R= 13.4 min (minor), 21.3 min (major).

(*S*)-3′-amino-7-methyl-2,5′,10′-trioxo-5′,10′-dihydrospiro[indoline-3,1′-pyrazolo[1,2-*b*]phthalazine]-2′-carbonitrile (**4n**): light yellow solid, m.p.: 187.5-188.5 °C; ^{1}H NMR (500 MHz, DMSO-d_6) δ 10.96 (s, 1H), 8.41 – 8.24 (m, 3H), 8.11 – 7.94 (m, 3H), 7.28 (dd, *J* = 7.5, 1.0 Hz, 1H), 7.13 (dt, *J* = 7.5, 1.0 Hz, 1H), 6.92 (t, *J* = 7.5 Hz, 1H), 2.27 (s, 3H);^{13}C NMR (125 MHz, DMSO-d_6) δ 173.0, 156.3, 152.5, 151.7, 140.7, 135.1, 134.4, 131.7, 128.6, 127.9, 127.6, 127.0, 125.1, 122.4, 121.9, 119.5, 114.4, 70.1, 60.3; HRMS (ESI) m/z: $[M+Na]^+$ calcd for $C_{20}H_{13}N_5O_3Na^+$ 394.0911, found 394.0915; $[\alpha]_D^{25}$= -42.0 (c 0.46, MeOH)(95% ee); HPLC (Chiralpak AS, hexane:^{i}PrOH = 70:30, 1.0 mL/min, 254 nm), t_R= 12.6 min (minor), 27.2 min (major).

(*S*)-3′-amino-7-nitro-2,5′,10′-trioxo-5′,10′-dihydrospiro[indoline-3,1′-pyrazolo[1,2-*b*]phthalazine]-2′-carbonitrile (**4o**): light brown solid, m.p.: 212.6-213.5 °C; ^{1}H NMR (500 MHz, DMSO-d_6) δ 11.77 (s, 1H), 8.49 (s, 2H), 8.36 – 8.29 (m, 1H), 8.15 (dd, *J* = 8.5, 1.0 Hz, 1H), 8.08 – 8.00 (m, 4H), 7.28 (dd, *J* = 8.5, 7.5 Hz, 1H);^{13}C NMR (125 MHz, DMSO-d_6) δ 173.0, 156.3, 152.7, 152.1, 138.2, 135.1, 134.5, 131.3, 131.0, 128.9, 128.7, 127.6, 127.4, 127.0, 125.4, 122.8, 114.0, 68.2, 58.8; HRMS (ESI) m/z: $[M+Na]^+$ calcd for $C_{19}H_{10}N_6O_5Na^+$ 425.0605, found 425.0608; $[\alpha]_D^{25}$= -41.5 (c 0.52, MeOH)(93% ee); HPLC (Chiralpak AD, hexane:^{i}PrOH = 70:30, 1.0 mL/min, 254 nm), t_R= 24.1 min (minor), 39.8 min (major).

(*S*)-3′-amino-1-methyl-2,5′,10′-trioxo-5′,10′-dihydrospiro[indoline-3,1′-pyrazolo[1,2-*b*]phthalazine]-2′-carbonitrile (**4p**) (known compound, lit.[1a]): white solid, m.p.: 282.8-284.0 °C; ^{1}H NMR (500 MHz, DMSO-d_6) δ 8.38 (s, 2H), 8.33 – 8.28 (m, 1H), 8.06 – 7.97 (m, 3H), 7.54 (dd, *J* = 7.5, 1.0 Hz, 1H), 7.42 (td, *J* = 7.5, 1.5 Hz, 1H), 7.15 (d, *J* = 8.0 Hz, 1H), 7.10 (td, *J* = 7.5, 1.0 Hz, 1H), 3.25 (s, 3H); $[\alpha]_D^{25}$=

-4.6 (c 0.35, MeOH)(>99% ee); HPLC (Chiralpak OJ, hexane:^{i}PrOH = 70:30, 1.0 mL/min, 254 nm), t_R= 17.9 min (major), 27.2 min (minor).

(*S*)-3′-amino-1-benzyl-2,5′,10′-trioxo-5′,10′-dihydrospiro[indoline-3,1′-pyrazolo[1,2-*b*]phthalazine]-2′-carbonitrile (4q) (known compound, lit.[1a]): light brown solid, m.p.: 264.6-265.7 ^{o}C; ^{1}H NMR (500 MHz, DMSO-d_6) δ 8.42 (s, 2H), 8.35 – 8.29 (m, 1H), 8.14 – 8.06 (m, 1H), 8.06 – 7.97 (m, 2H), 7.59 (dd, *J* = 7.5, 1.5 Hz, 1H), 7.49 – 7.41 (m, 2H), 7.38 – 7.32 (m, 2H), 7.30 (td, *J* = 8.0, 1.5 Hz, 2H), 7.07 (td, *J* = 7.5, 1.0 Hz, 1H), 6.90 (d, *J* = 8.0 Hz, 1H), 5.12 – 4.95 (m, 2H); $[\alpha]_D^{25}$= -4.6 (c 0.33, MeOH)(88% ee); HPLC (Chiralpak AD, hexane:^{i}PrOH = 70:30, 1.0 mL/min, 254 nm), t_R= 26.0 min (minor), 42.8 min (major).

(*S*)-3′-amino-5-chloro-1-benzyl-2,5′,10′-trioxo-5′,10′-dihydrospiro[indoline-3,1′-pyrazolo[1,2-*b*]phthalazine]-2′-carbonitrile (4r): light yellow solid, m.p.: 253.7-254.3 ^{o}C; ^{1}H NMR (500 MHz, DMSO-d_6) δ 8.46 (s, 2H), 8.37 – 8.30 (m, 1H), 8.10 (dd, *J* = 7.5, 2.0 Hz, 1H), 8.07 – 7.98 (m, 2H), 7.82 (d, *J* = 2.0 Hz, 1H), 7.43 (d, *J* = 7.0 Hz, 2H), 7.40 – 7.26 (m, 4H), 6.93 (d, *J* = 8.5 Hz, 1H), 5.05 (dd, *J* = 16.0, 6.0 Hz, 2H); ^{13}C NMR (125 MHz, DMSO-d_6) δ 171.1, 156.5, 152.7, 152.1, 141.3, 135.1, 135.1, 134.5, 130.2, 128.9, 128.6, 127.6, 127.5, 127.1, 127.0, 127.0, 124.9, 114.3, 111.3, 69.2, 59.2, 43.5; HRMS (ESI) m/z: $[M+Na]^+$ calcd for $C_{26}H_{16}ClN_5O_3Na^+$ 504.0834, found 504.0838; $[\alpha]_D^{25}$= 52.0(c 0.50, MeOH)(92% ee); HPLC (Chiralpak AD-H, hexane:^{i}PrOH = 80:20, 1.0 mL/min, 254 nm), t_R= 44.2 min (minor), 60.5 min (major).

(*S*)-3′-amino-2,5′,10′-trioxo-5′,10′-dihydrospiro[indoline-3,1′-pyrazolo[1,2-*b*]phthalazine]-2′-methyl acetate (4s) (known compound, lit.[1a]): light yellow solid, m.p.: 317.6-318.6 ^{o}C; ^{1}H NMR (400 MHz, DMSO) δ 10.73 (s, 1H), 8.36 – 8.24 (m, 1H), 8.08 – 7.97 (m, 3H), 7.30 (d, *J* = 7.4 Hz, 1H), 7.21 (t, *J* = 7.7 Hz, 1H), 6.88 (t, *J* = 7.5 Hz, 1H), 6.83 (d, *J* = 7.8 Hz, 1H), 3.44 (s, 3H); $[\alpha]_D^{25}$= -51.0 (c -0.34, MeOH) (91% ee); HPLC (Chiralcel OJ, hexane:^{i}PrOH = 80:20, 1.0 mL/min, 254 nm), t_R= 45.7 min (major), 49.8 min (minor).

(*S*)-3′-amino-2,5′,10′-trioxo-5′,10′-dihydrospiro[indoline-3,1′-pyrazolo[1,2-*b*]phthalazine]-2′-ethyl acetate (4t) (known compound, lit.[1a]):light yellow solid, m.p.:

284.5-285.3 °C; ^{1}H NMR (500 MHz, DMSO) δ 10.74 (s, 1H), 8.37 – 8.26 (m, 1H), 8.08 – 8.04 (m, 1H), 8.02 – 7.97 (m, 2H), 7.32 – 7.28 (m, 1H), 7.22 (td, *J* = 7.0, 1.0 Hz, 1H), 6.89 (td, *J* = 7.5, 1.0 Hz, 1H), 6.83 (dt, *J* = 7.5, 0.7 Hz, 1H), 3.85 (ddd, *J* = 11.0, 6.5, 3.0 Hz, 2H), 0.87 (t, *J* = 7.0 Hz, 3H); $[\alpha]_D^{25}$= -0.5 (c 0.42, MeOH)(98% ee); HPLC (Chiralcel OJ, hexane:^{i}PrOH = 80:20, 1.0 mL/min, 254 nm), t_R= 14.4 min (major), 26.1 min (minor).

(*S*)-3′-amino-5-bromo-2,5′,10′-trioxo-5′,10′-dihydrospiro[indoline-3,1′-pyrazolo[1,2-*b*]phthalazine]-2′-ethyl acetate (**4u**) (known compound, lit.[1a]): light yellow solid, m.p.: 330.8-331.8 °C; ^{1}H NMR (500 MHz, DMSO) δ 10.89 (s, 1H), 8.34 – 8.28 (m, 1H), 8.09 – 8.05 (m, 1H), 8.04 – 7.98 (m, 2H), 7.63 (d, *J* = 2.0 Hz, 1H), 7.40 (dd, *J* = 8.5, 2.0 Hz, 1H), 6.80 (d, *J* = 8.5 Hz, 1H), 3.89 (t, *J* = 8.0 Hz, 2H), 0.91 (t, *J* = 7.5 Hz, 3H); $[\alpha]_D^{25}$= 32.9 (c 0.47, MeOH)(99% ee); HPLC (Chiralpak AD, hexane:^{i}PrOH = 70:30, 1.0 mL/min, 254 nm), t_R=31.4 min (major), 43.1 min (minor).

(*S*)-3′-amino-7-bromo-2,5′,10′-trioxo-5′,10′-dihydrospiro[indoline-3,1′-pyrazolo[1,2-*b*]phthalazine]-2′-ethyl acetate (**4v**): light yellow solid, m.p.: 242.9-243.9 °C; ^{1}H NMR (500 MHz, $CDCl_3$) δ 8.41 – 8.32 (m, 1H), 8.28 – 8.17 (m, 1H), 7.92 – 7.82 (m, 2H), 7.79 (s, 1H), 7.41 (dd, *J* = 8.0, 1.0 Hz, 1H), 7.11 (dt, *J* = 7.0, 1.0 Hz, 1H), 6.91 (dd, *J* = 8.0, 7.5 Hz, 1H), 3.96 (s, 2H), 0.94 (s, 3H); ^{13}C NMR (125 MHz, $CDCl_3$) δ 172.1, 157.0, 153.4, 141.0, 134.9, 134.0, 132.6, 128.6, 128.0, 127.9, 124.2, 122.4, 103.3, 71.4, 59.8, 13.5; HRMS (ESI) m/z: $[M+Na]^+$ calcd for $C_{21}H_{15}BrN_4O_5Na^+$ 505.0118, found 505.013; $[\alpha]_D^{25}$= -265.4 (c 0.62, MeOH)(99% ee); HPLC (Chiralpak IA, hexane:^{i}PrOH = 80:20, 1.0 mL/min, 254 nm), t_R= 20.5 min (minor), 56.9 min (major).

(*S*)-3′-amino-1-methyl-2,5′,10′-trioxo-5′,10′-dihydrospiro[indoline-3,1′-pyrazolo[1,2-*b*]phthalazine]-2′-ethyl acetate (**4w**) (known compound, lit.[1c]): light yellow solid, m.p.: 287.5-288.4 °C; ^{1}H NMR (500 MHz, DMSO) δ 8.33 – 8.29 (m, 1H), 8.05 – 7.98 (m, 3H), 7.37 (dd, *J* = 7.5, 1.0 Hz, 1H), 7.33 (td, *J* = 7.5, 1.5 Hz, 1H), 7.05 (dd, *J* = 8.0, 1.0 Hz, 1H), 6.98 (td, *J* = 7.5, 1.0 Hz, 1H), 3.80 (dd, *J* = 14.0, 7.0 Hz, 2H), 3.22 (s, 3H), 0.82 (d, *J* = 8.0 Hz, 3H); $[\alpha]_D^{25}$= -226.2 (c 0.51, MeOH)(99%

ee); HPLC (Chiralpak AS, hexane:^{i}PrOH = 80:20, 1.0 mL/min, 254 nm), t_R= 18.5 min (major), 25.0 min (minor).

[1] (a) X. N. Zhang, Y. X. Li and Z. H. Zhang, Nickel chloride-catalyzed one-pot three-component synthesis of pyrazolophthalazinyl spirooxindoles, *Tetrahedron*, 2011, **67**, 7426-7430; (b) H. Chen and D. Q. Shi, Efficient One-Pot Synthesis of Spiro[indoline-3,10-pyrazolo[1,2-b]phthalazine] Derivatives via Three-Component Reaction, *J. Heterocyclic Chem.*, 2013, **50**, 56-60; (c) J. X. Wang, X. G. Bai, C. L. Xu, Y. C. Wang, W. Lin, Y. Zou and D. Q. Shi, Ultrasound-Promoted One-Pot, Three-Component Synthesis of Spiro[indoline-3,1'-pyrazolo[1,2-*b*]phthalazine] Derivatives, *Molecules*, 2012, **17**, 8674-8686

3. ^{1}H NMR and ^{13}C NMR spectra

4a

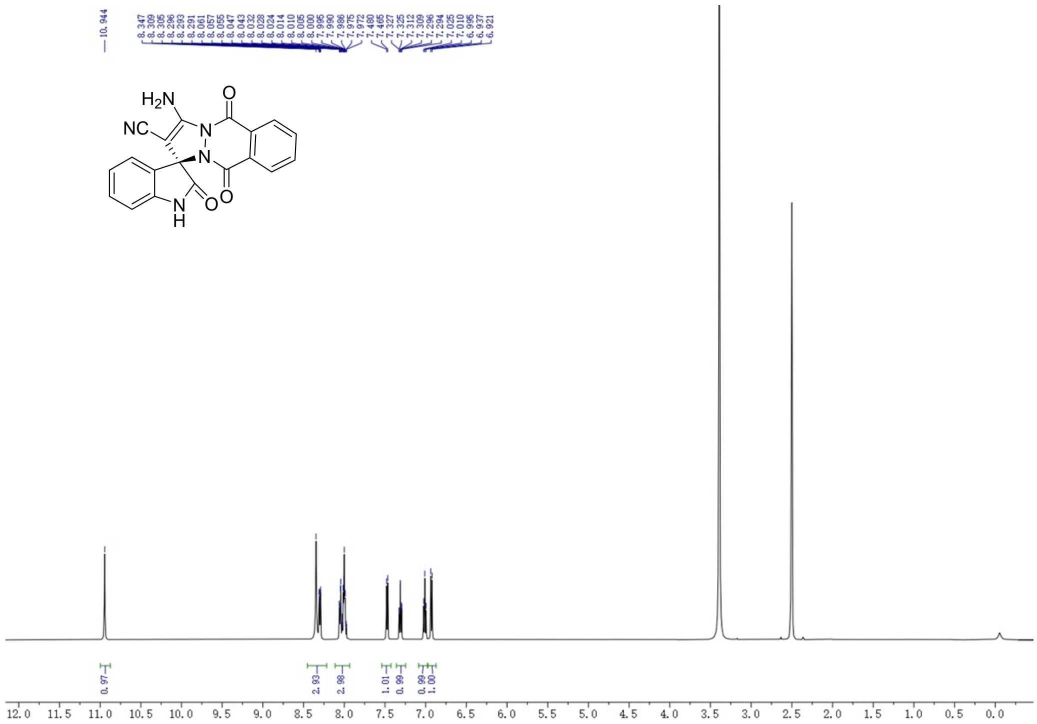

4b

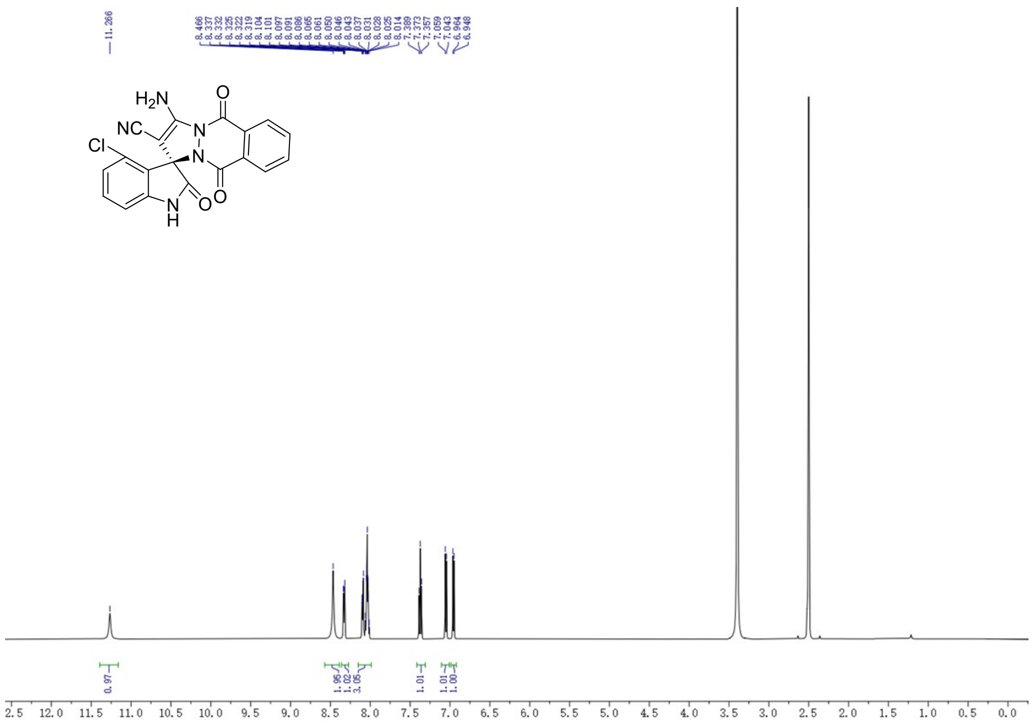

4c

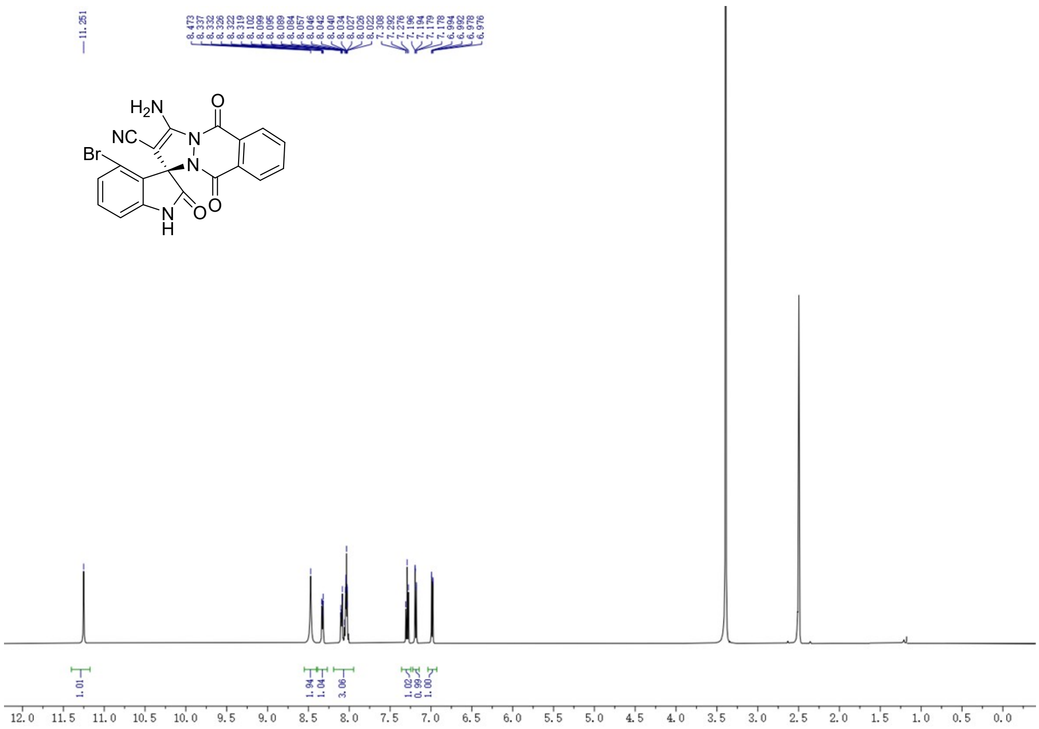

4d

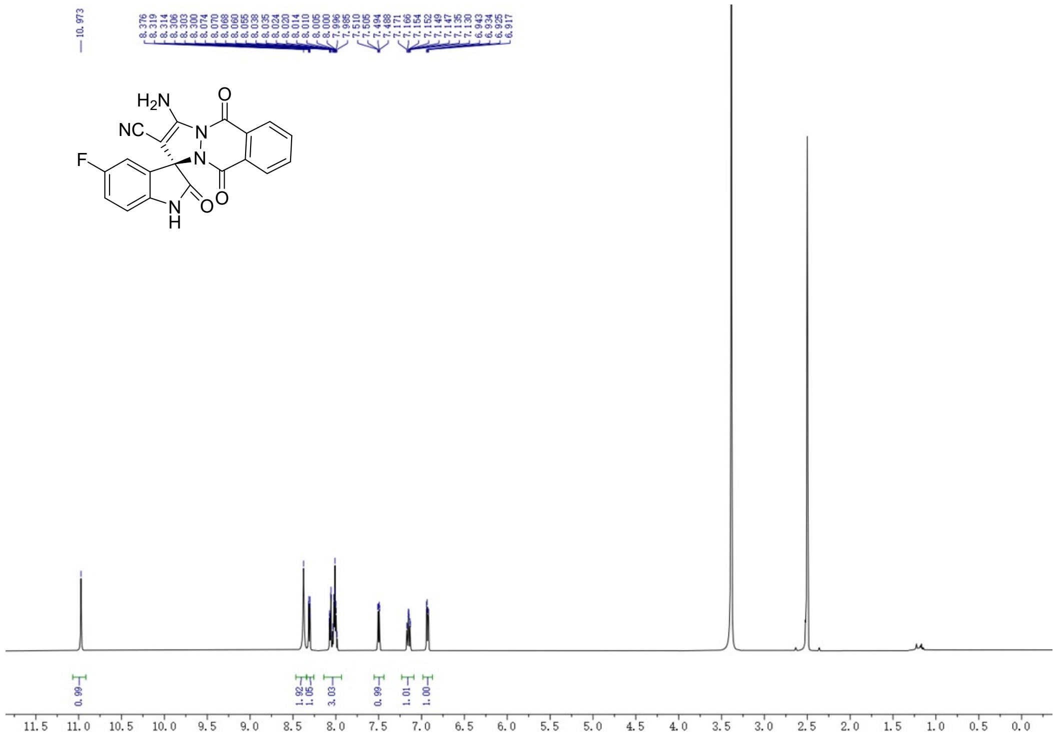

4e

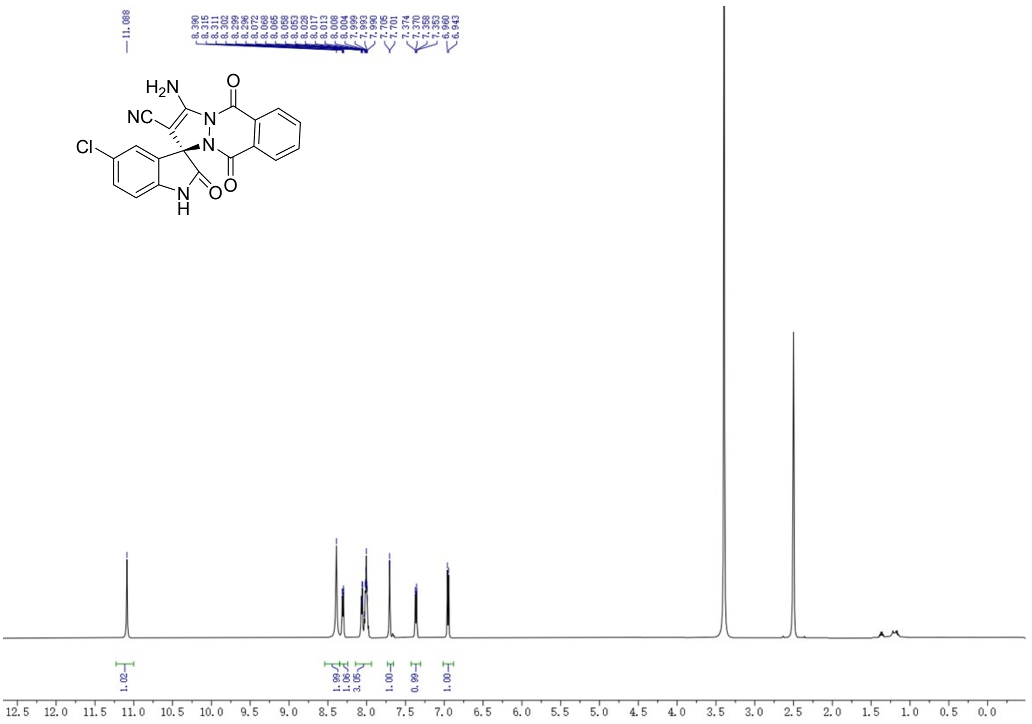

4f

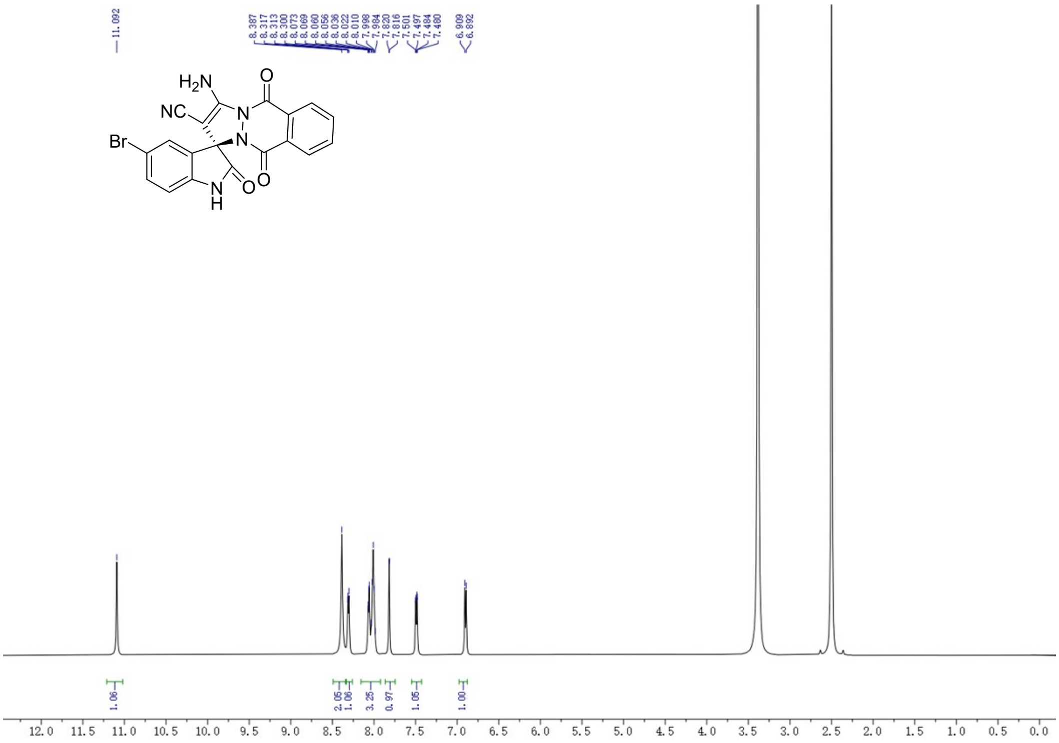

4g

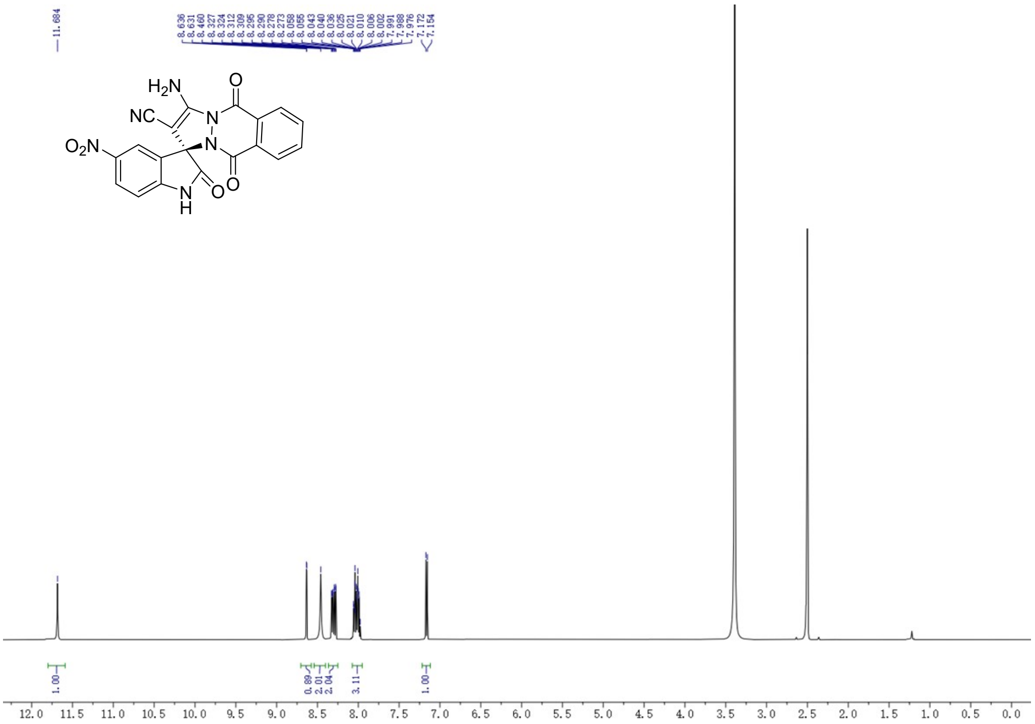

4h

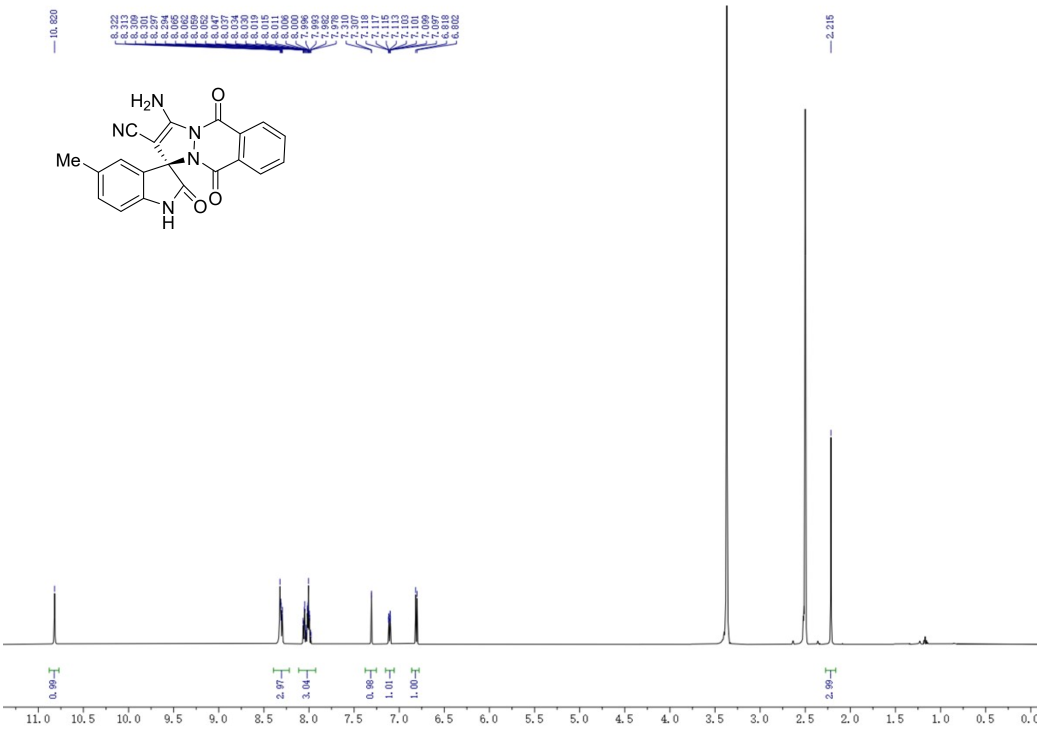

4i

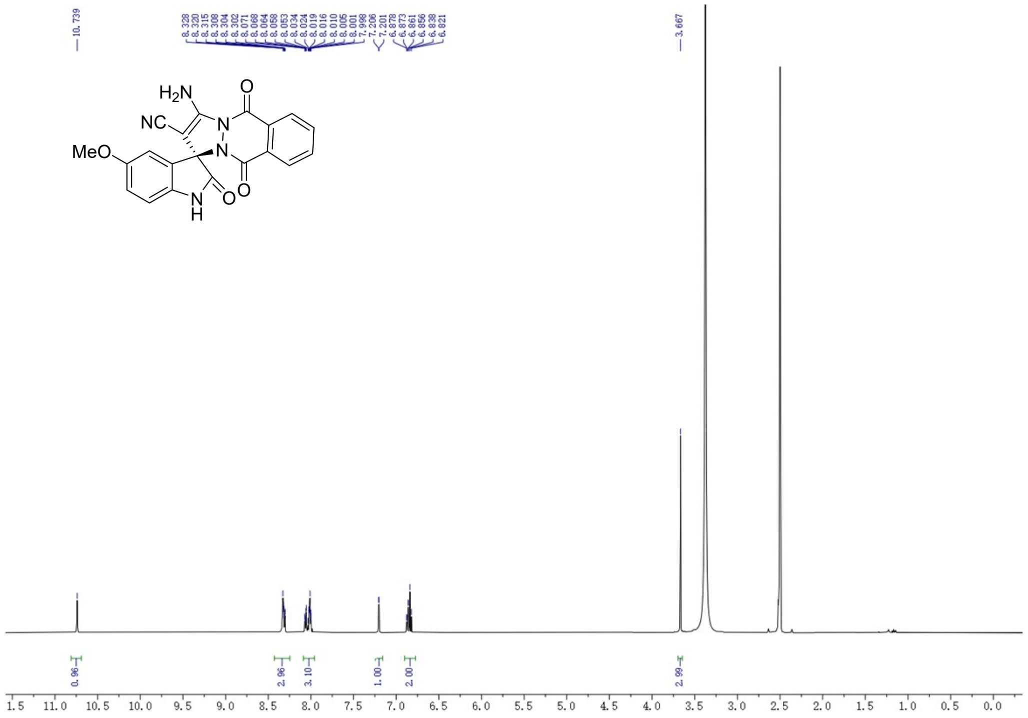

10.739
3.667
H2N
NC
MeO
O
N
N
NH
O
O

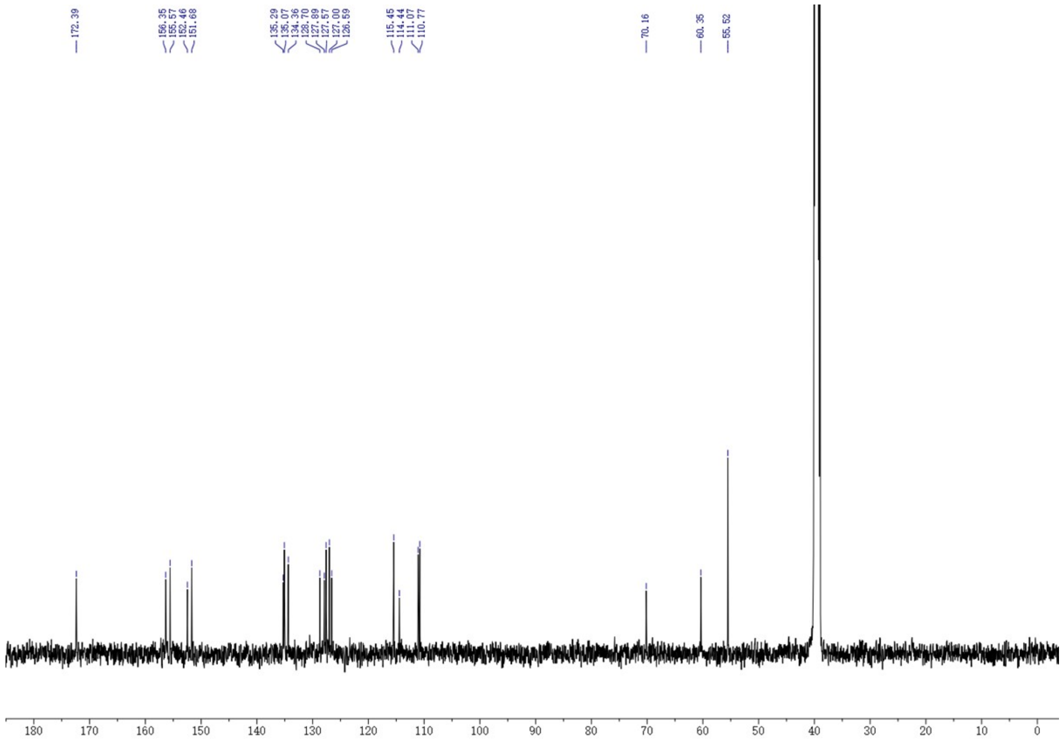

172.39
156.35
155.57
152.46
151.68
135.29
135.07
134.36
128.70
127.89
127.57
127.00
126.59
115.45
114.44
111.07
110.77
70.16
60.35
55.52

4j

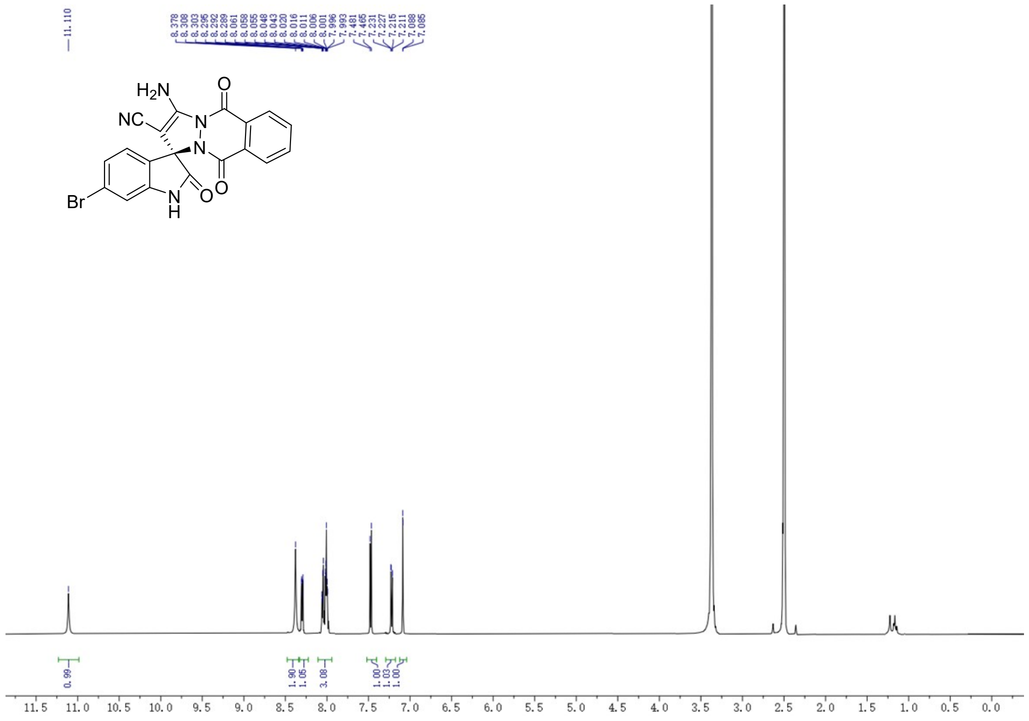

4k

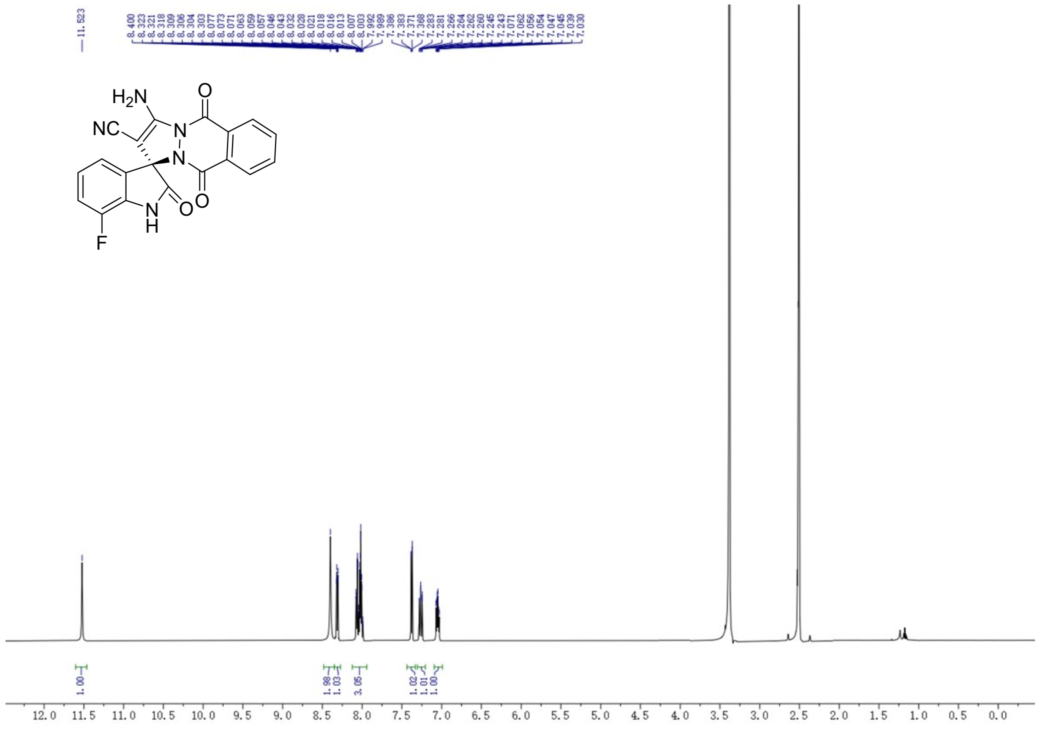

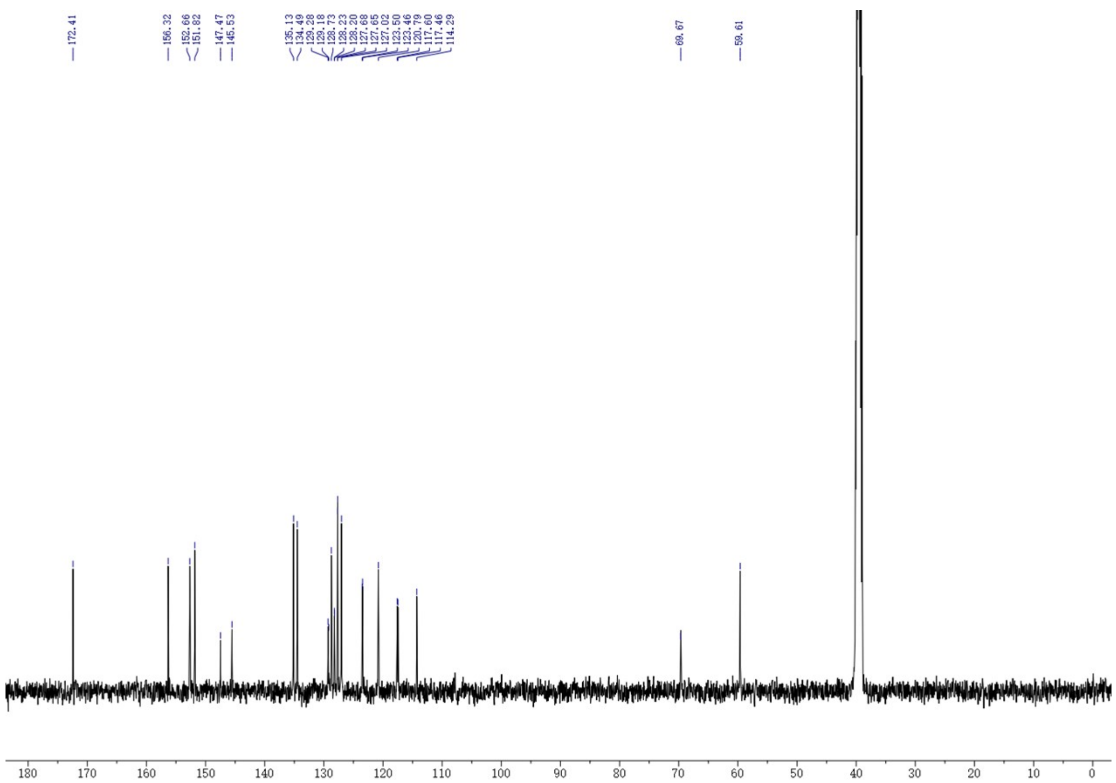

4l

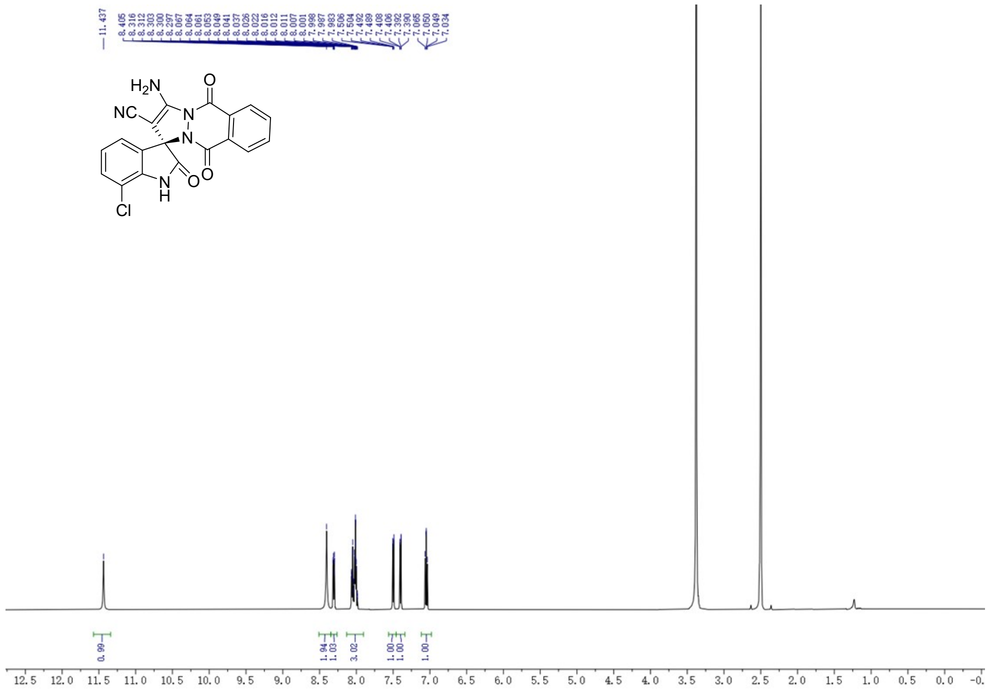

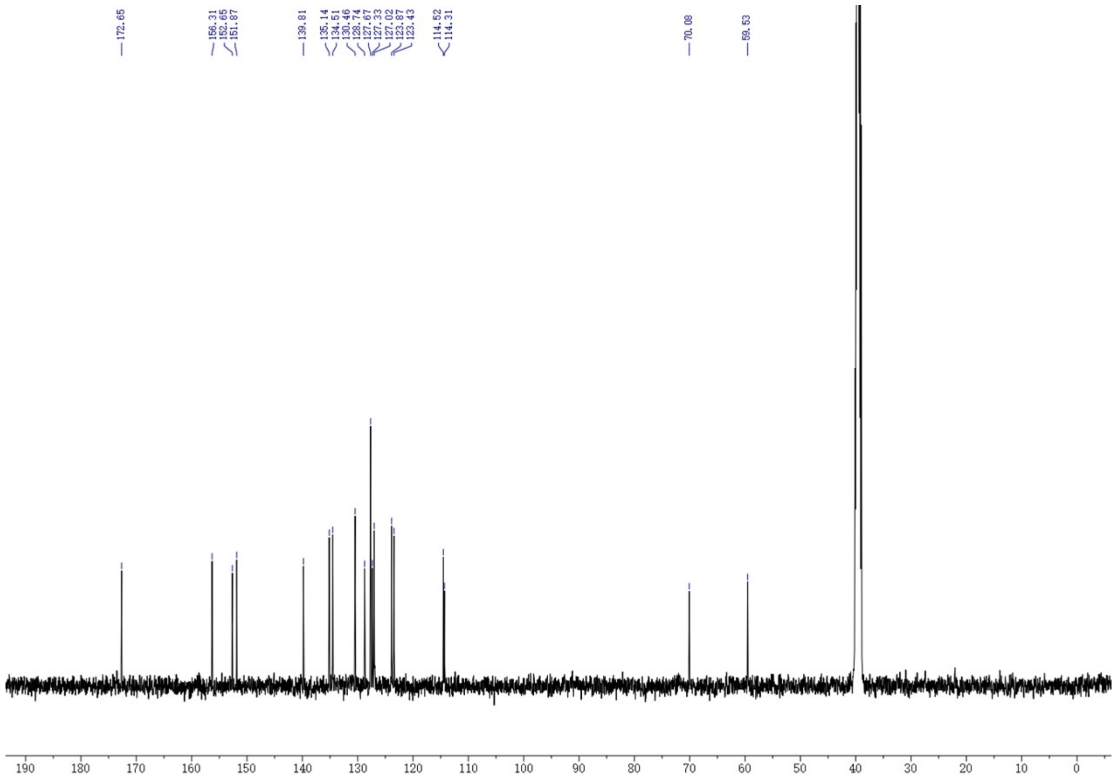
172.65
156.31
152.65
151.87
139.81
135.14
134.51
130.46
128.74
127.67
127.33
127.02
123.87
123.43
114.52
114.31
70.08
59.53
190 180 170 160 150 140 130 120 110 100 90 80 70 60 50 40 30 20 10 0

4m

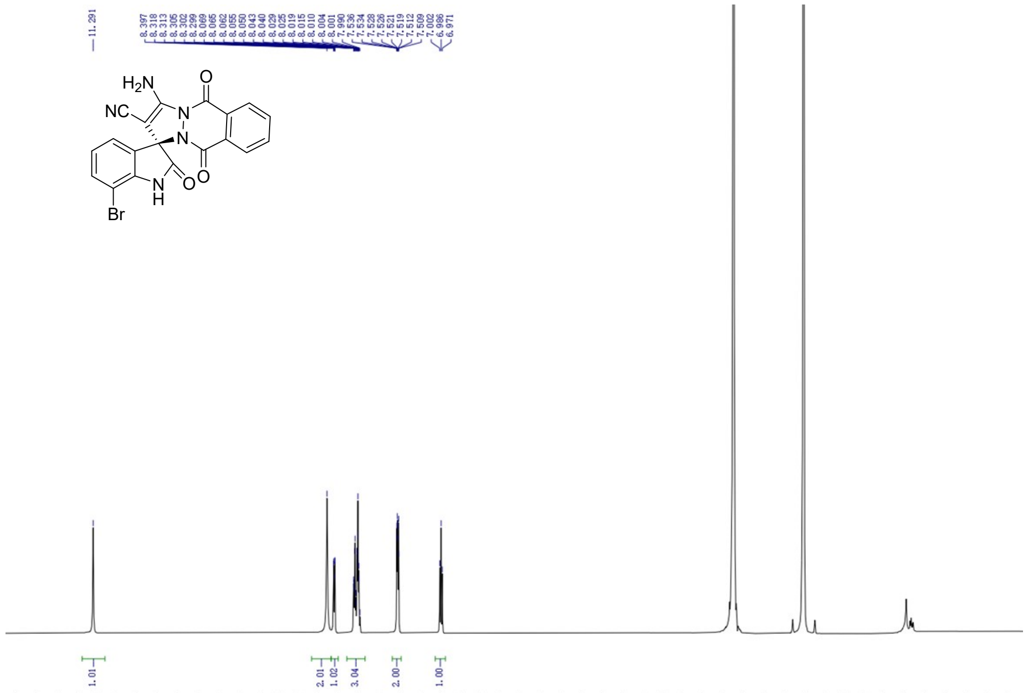
11.291
H_2N
NC
O
N
N
O
O
N
H
Br
2.01
1.02
3.04
2.00
1.00
1.01
12.0 11.5 11.0 10.5 10.0 9.5 9.0 8.5 8.0 7.5 7.0 6.5 6.0 5.5 5.0 4.5 4.0 3.5 3.0 2.5 2.0 1.5 1.0 0.5 0.0

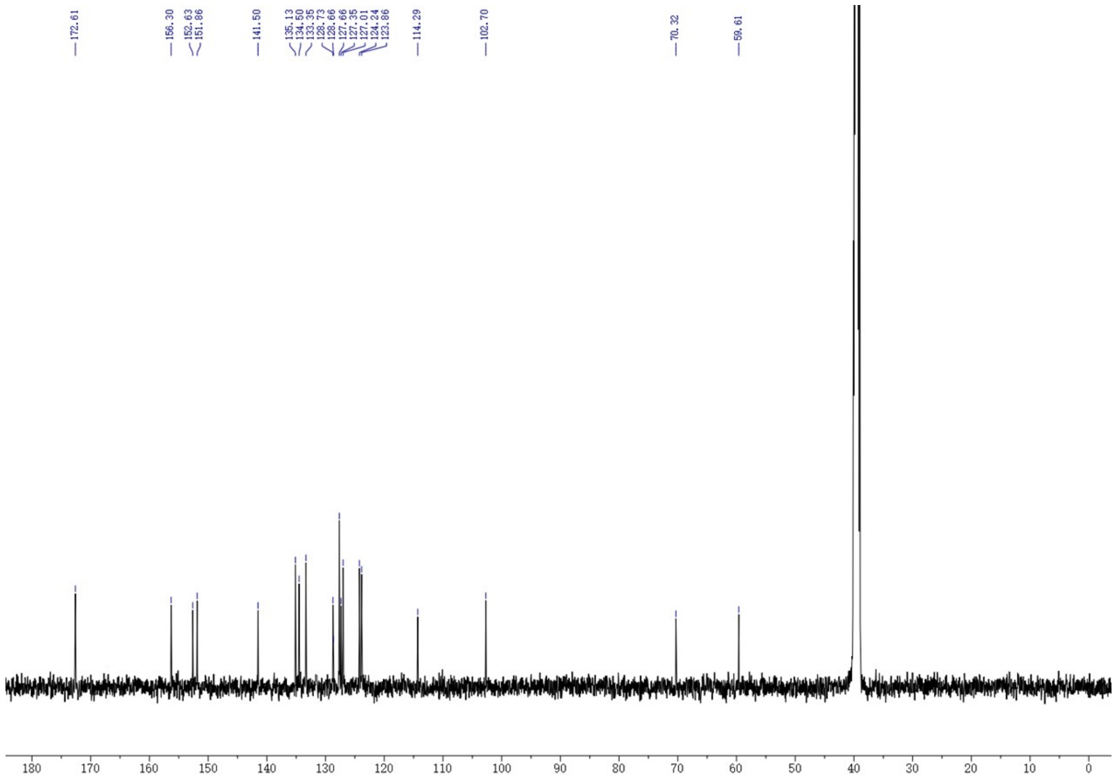

4n

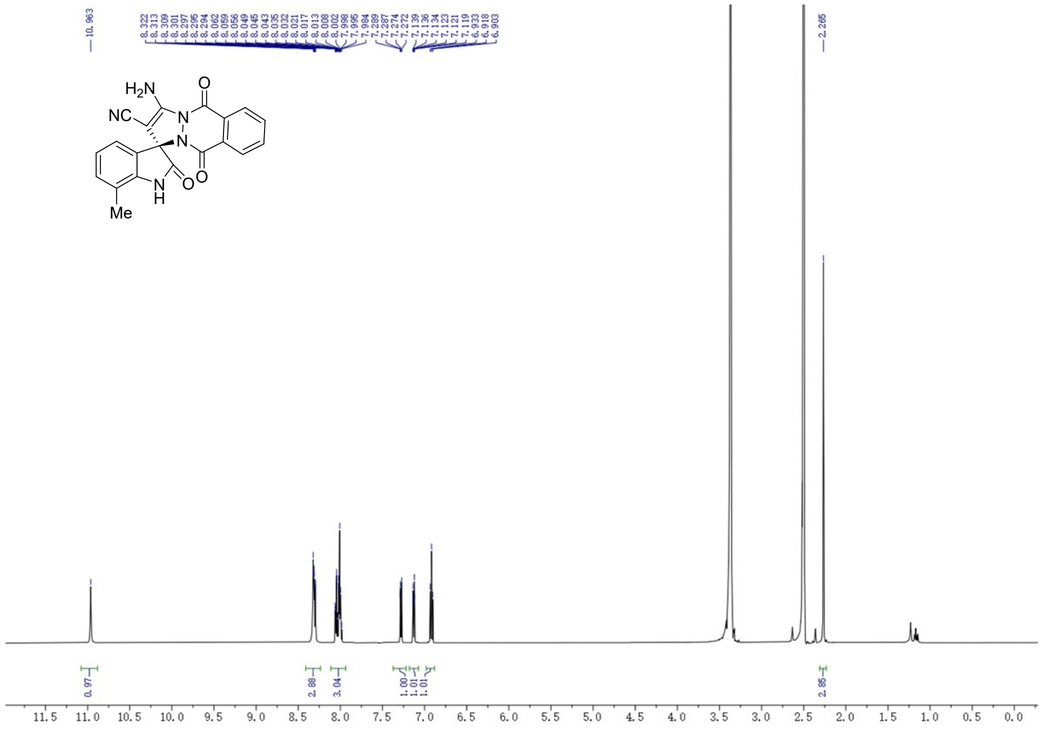

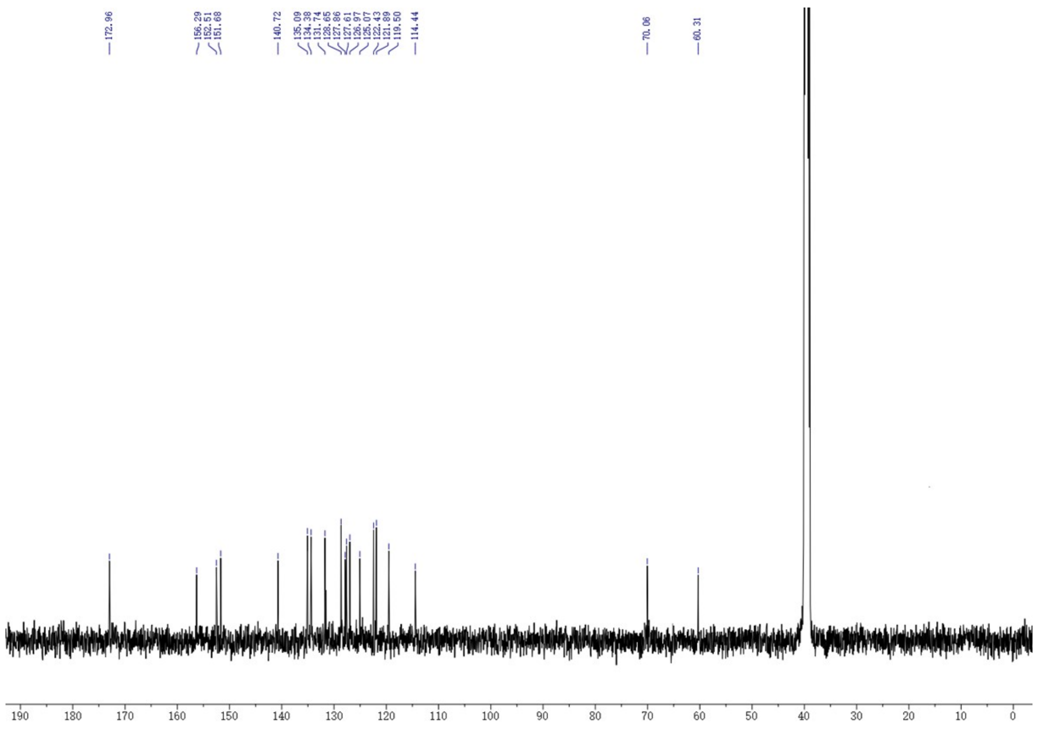

4o

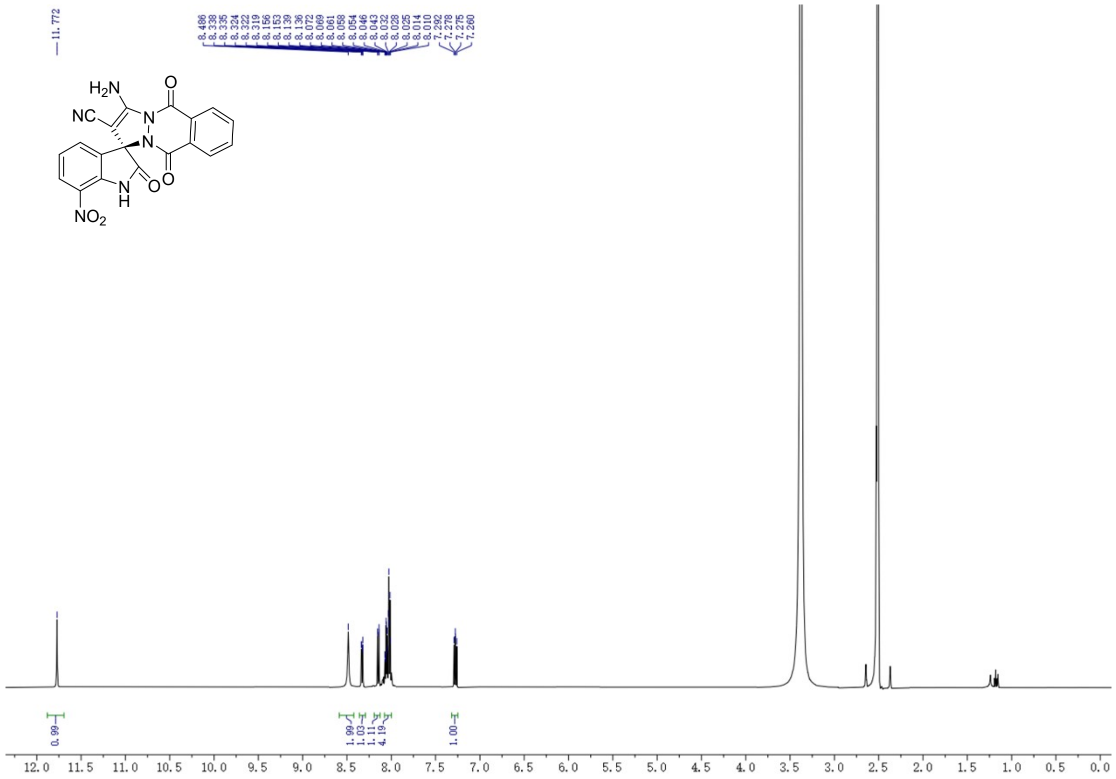

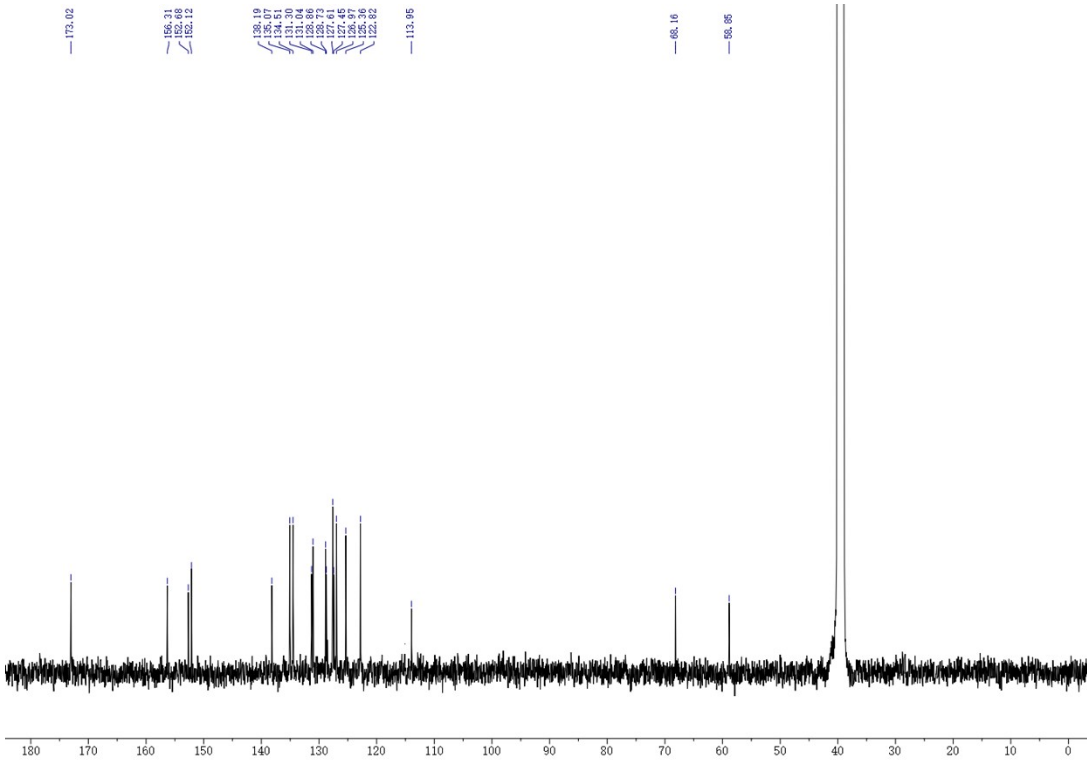

4p

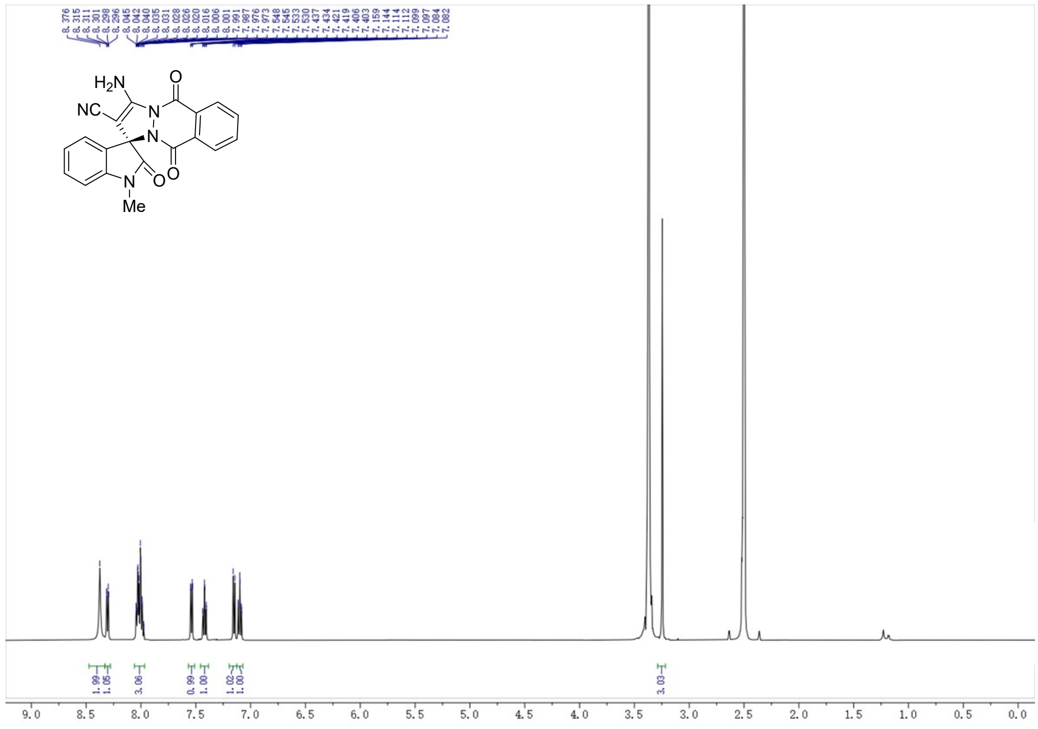

4q

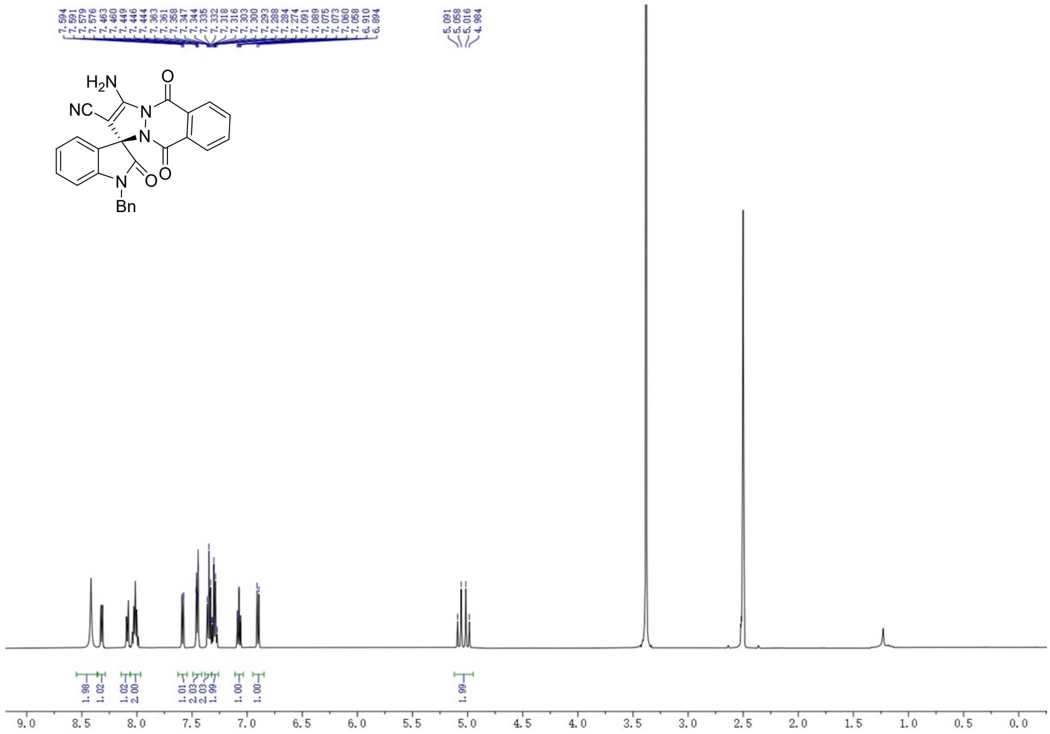

4r

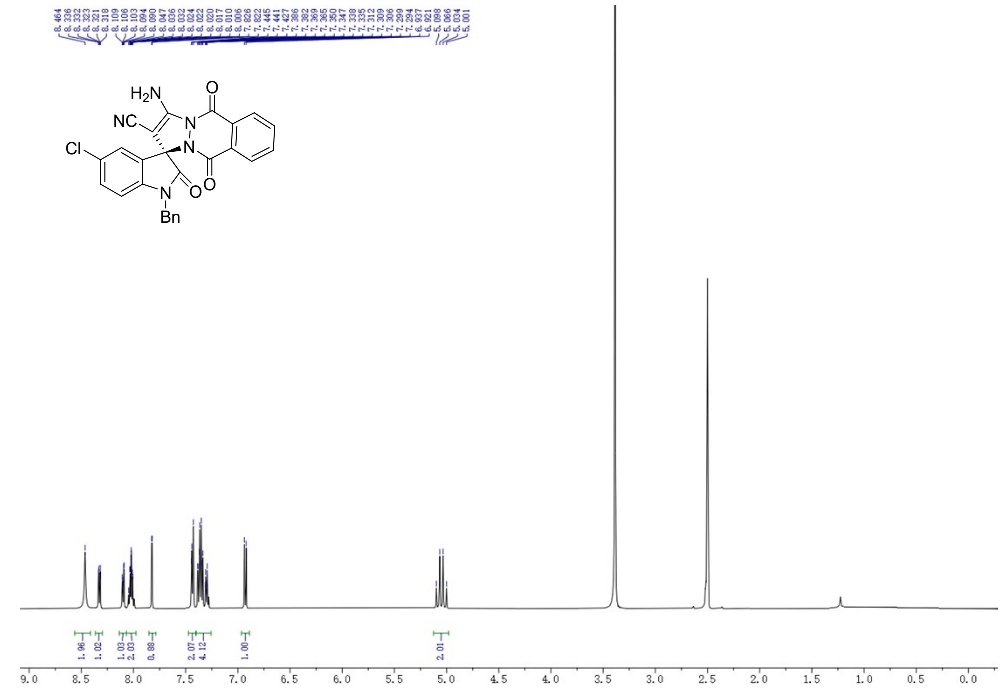

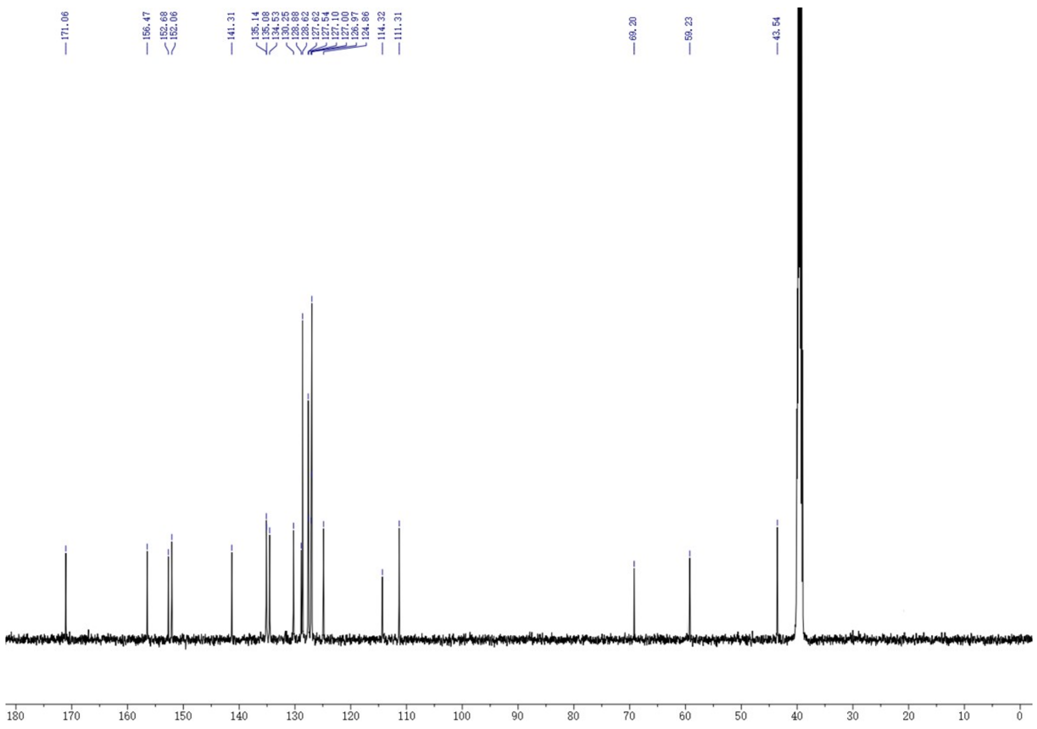
171.06
156.47
152.68
152.06
141.31
135.14
135.08
134.53
130.25
128.88
128.62
127.62
127.54
127.10
127.00
126.97
124.86
114.32
111.31
69.20
59.23
43.54
180 170 160 150 140 130 120 110 100 90 80 70 60 50 40 30 20 10 0

4s

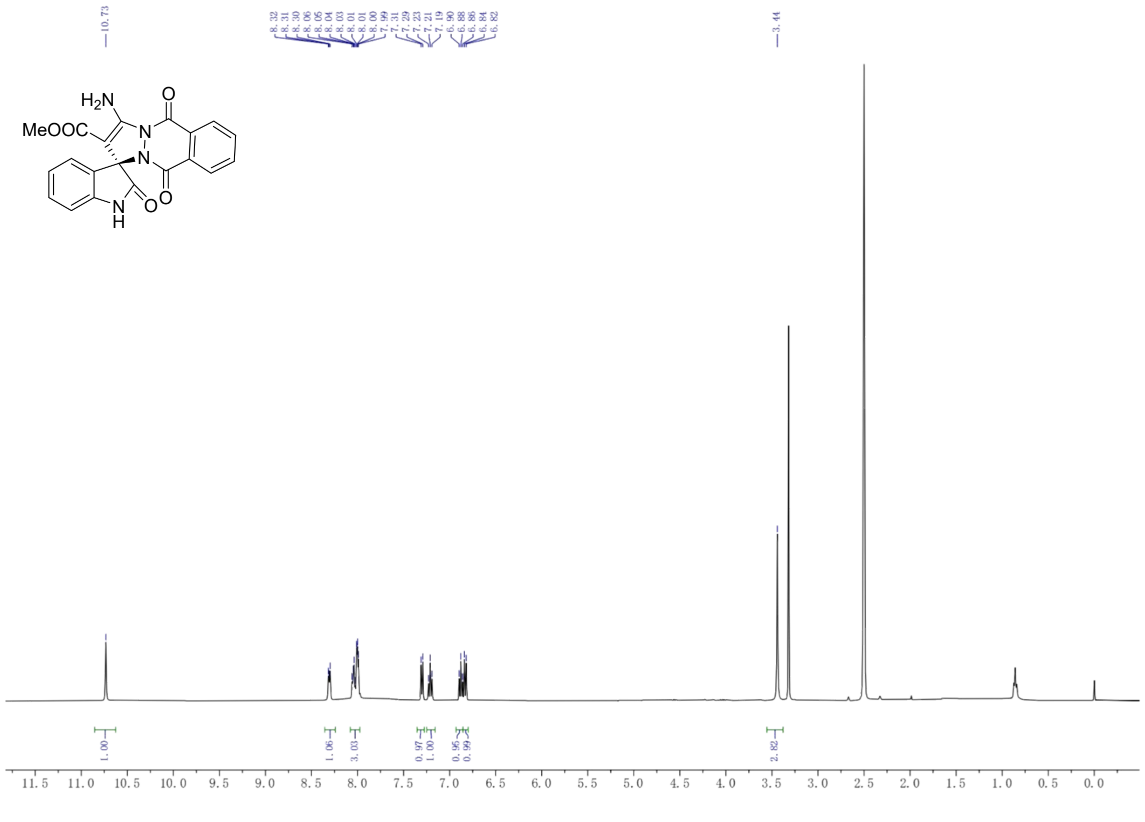
10.73
8.32
8.31
8.30
8.06
8.05
8.04
8.03
8.01
8.01
8.00
7.99
7.31
7.29
7.23
7.21
7.19
6.90
6.88
6.84
6.82
3.44
H_2N
O
MeOOC
N
N
O
O
NH
1.00
1.06
3.03
0.97
1.00
0.95
0.99
2.82
11.5 11.0 10.5 10.0 9.5 9.0 8.5 8.0 7.5 7.0 6.5 6.0 5.5 5.0 4.5 4.0 3.5 3.0 2.5 2.0 1.5 1.0 0.5 0.0

4t

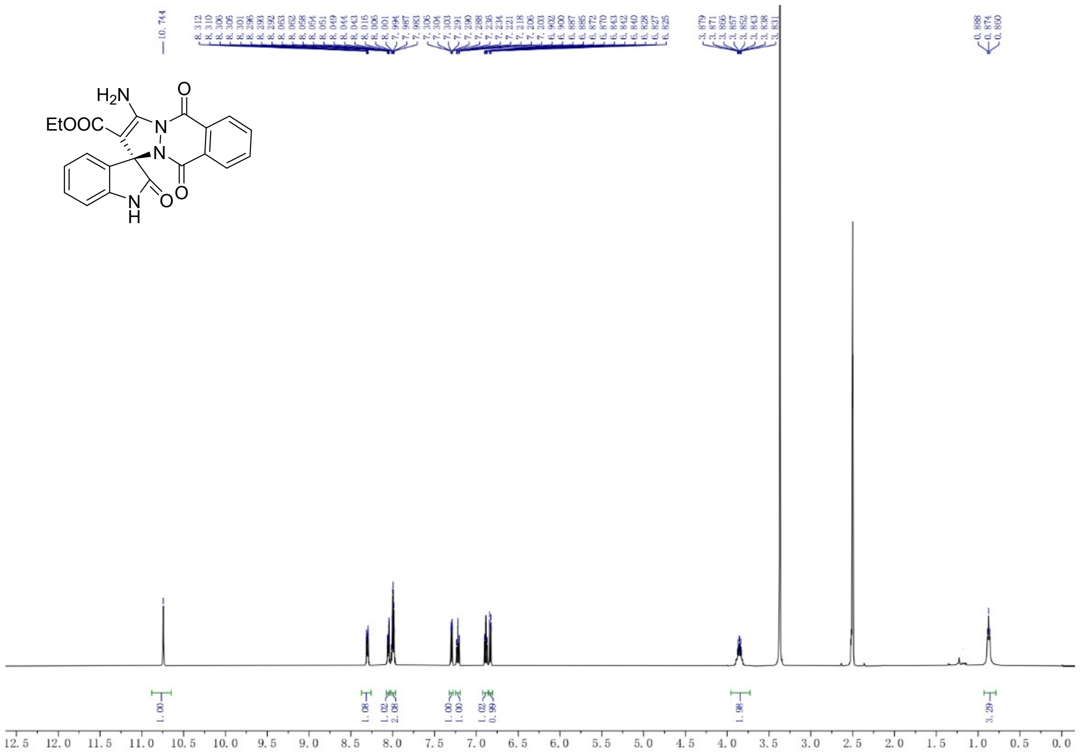

4u

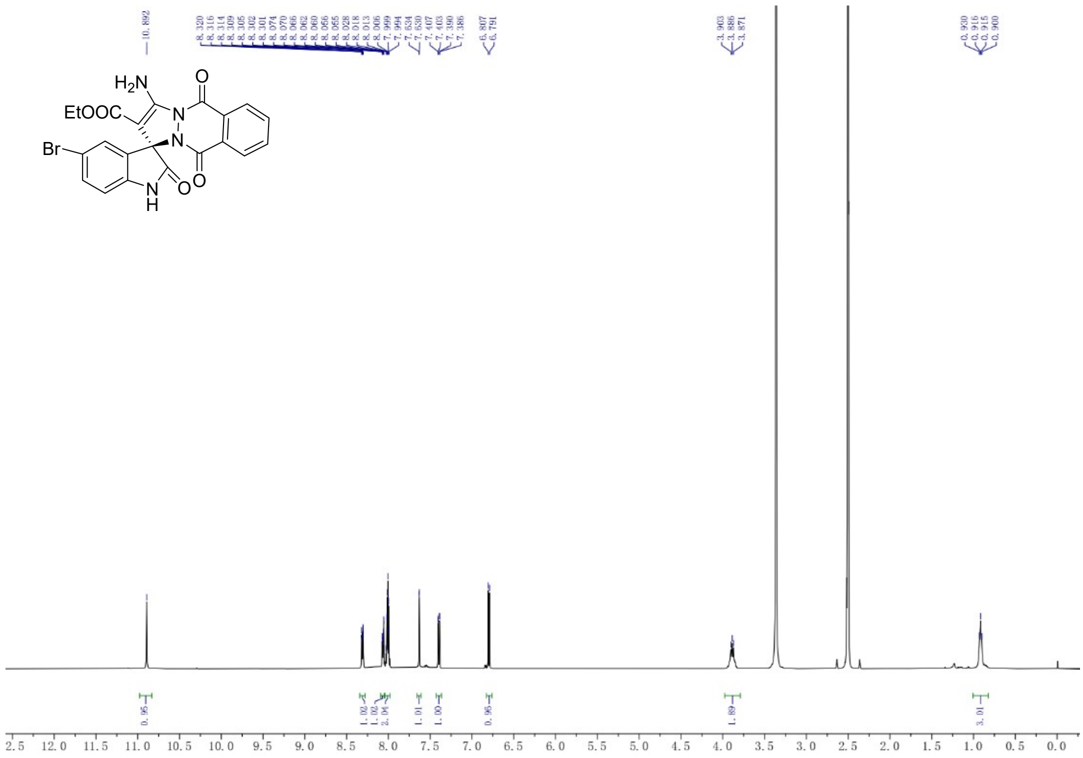

4v

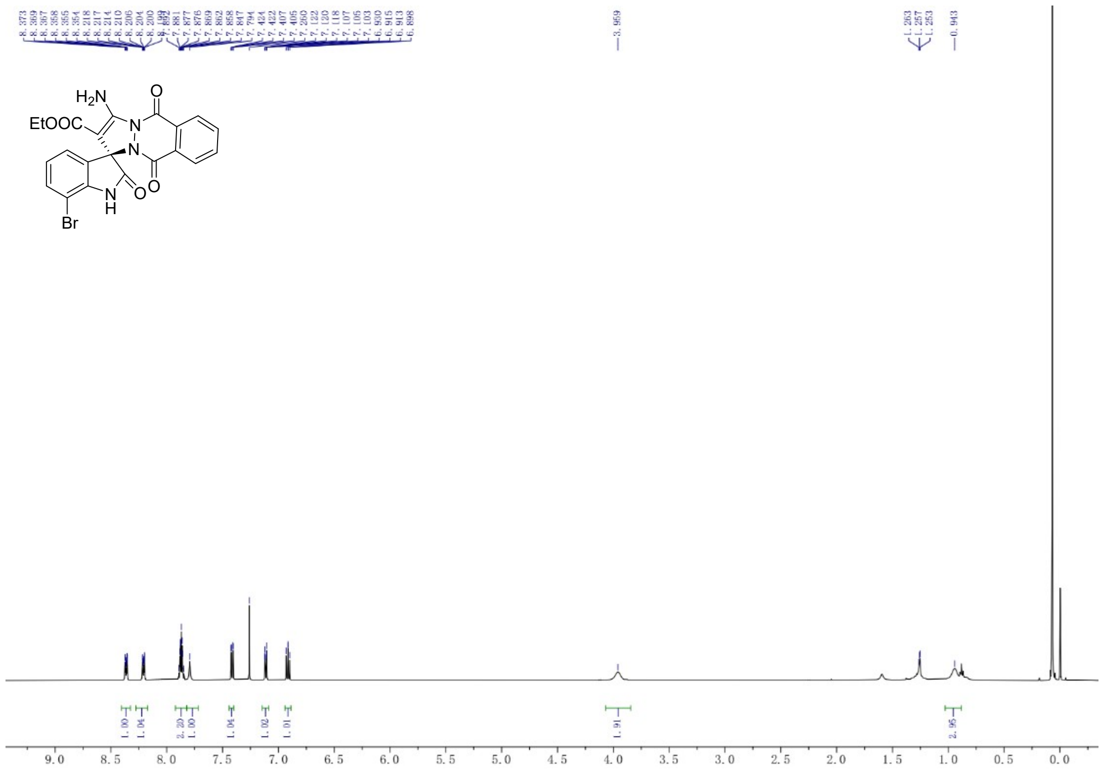

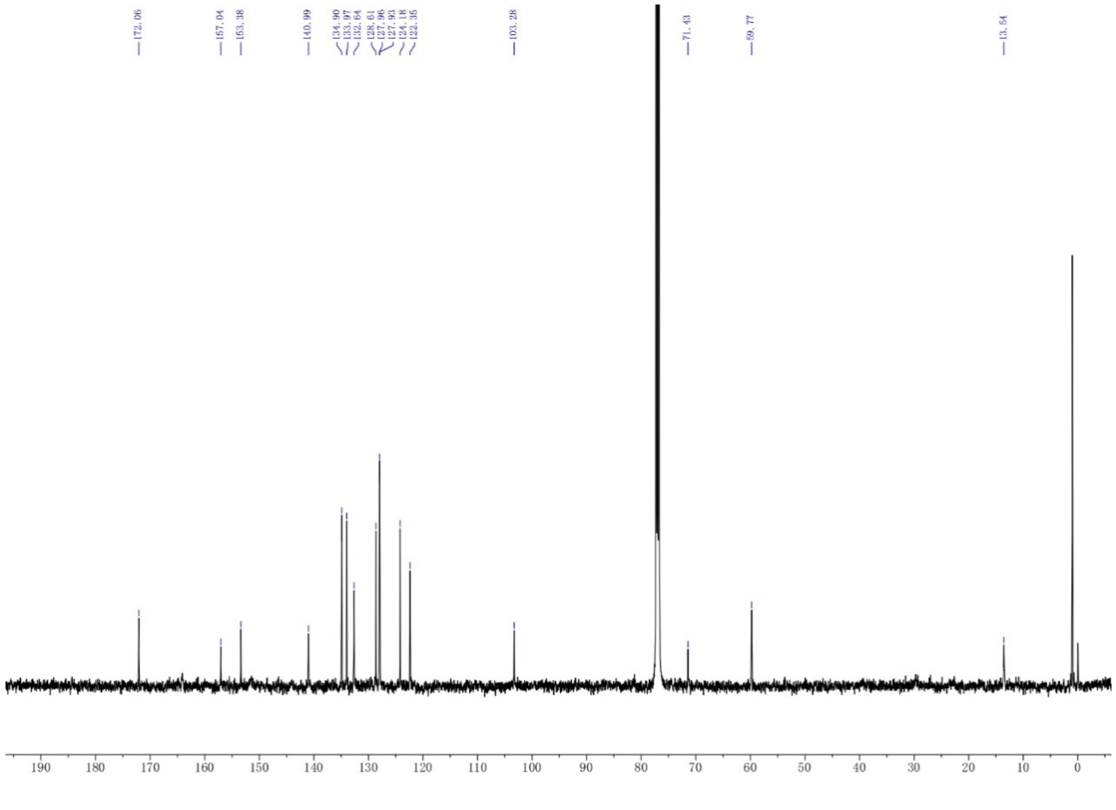

4w

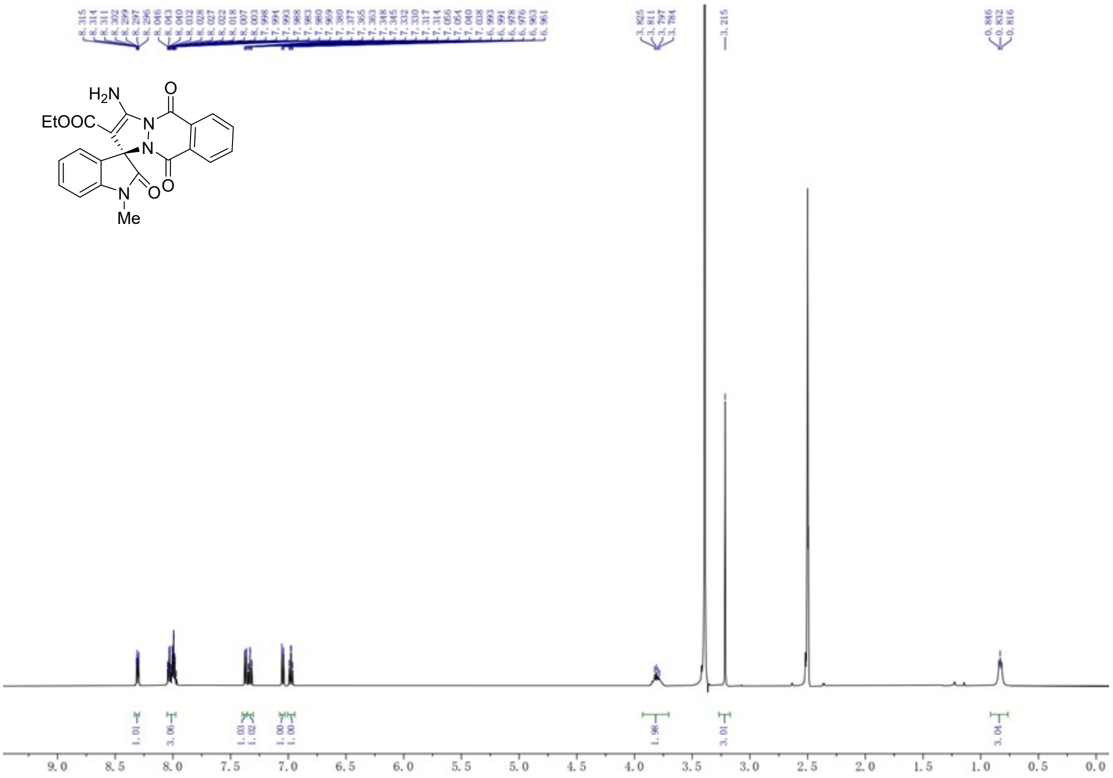
H2N
EtOOC
N
O
Me
9.0
8.5
8.0
7.5
7.0
6.5
6.0
5.5
5.0
4.5
4.0
3.5
3.0
2.5
2.0
1.5
1.0
0.5
0.0

4. HPLC trace

4a

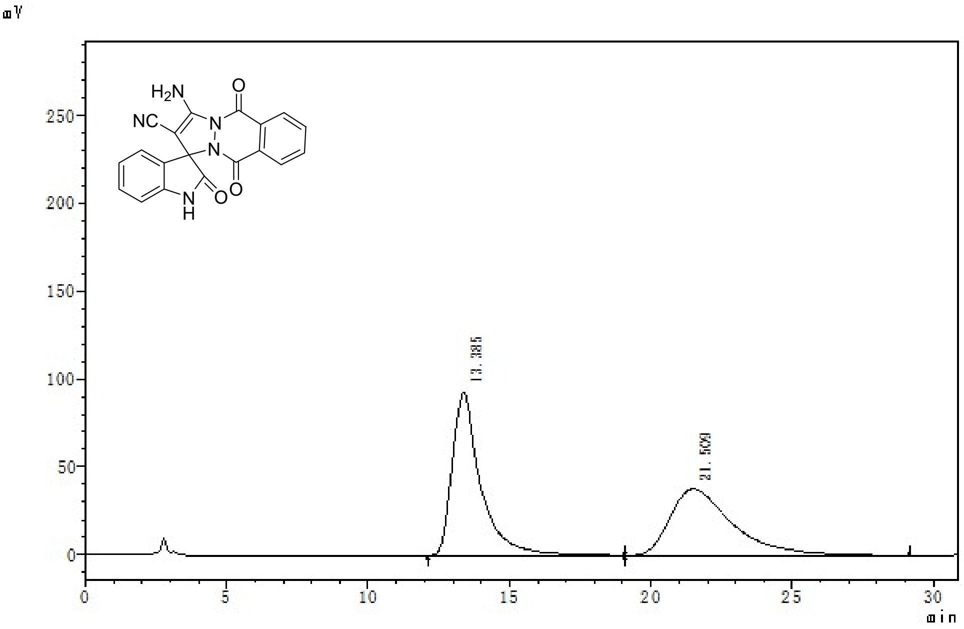

	Retention Time	Area	Height	Area%
1	13.385	6583126	93212	52.839
2	21.509	5875620	37888	47.161
Total		12458747	131100	100.000

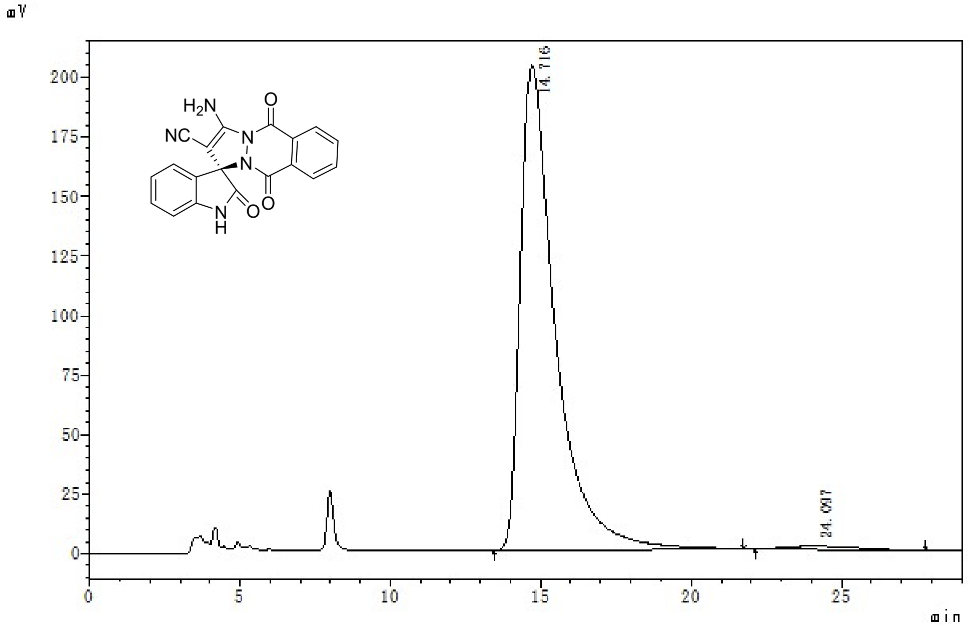

	Retention Time	Area	Height	Area%
1	14.716	16541510	203631	98.828
2	24.097	196178	1298	1.172
Total		16737688	204929	100.000

4b

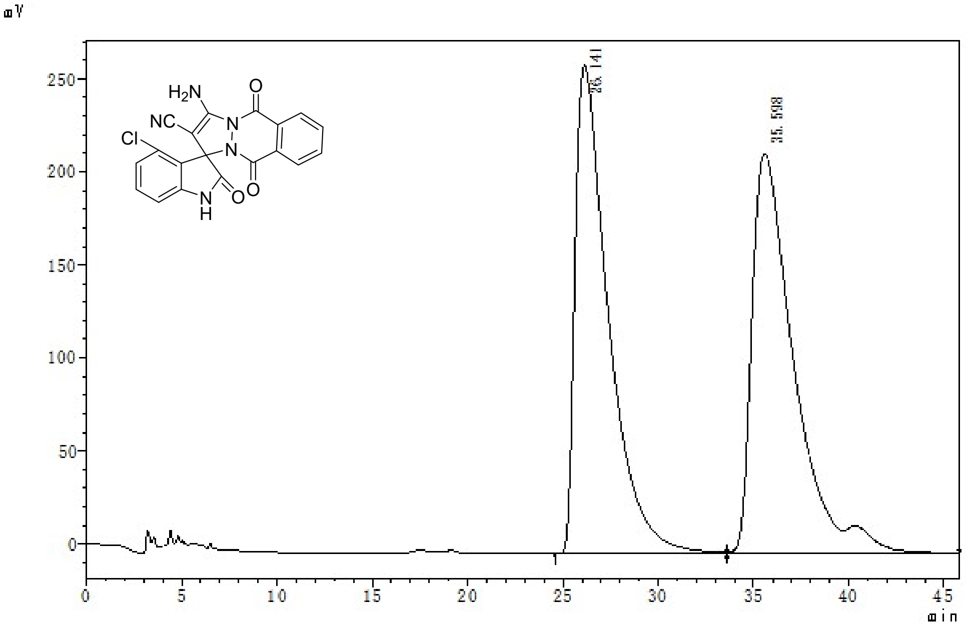

	Retention Time	Area	Height	Area%
1	26.141	32024460	262815	49.274
2	35.598	32967868	215022	50.726
Total		64992328	477838	100.000

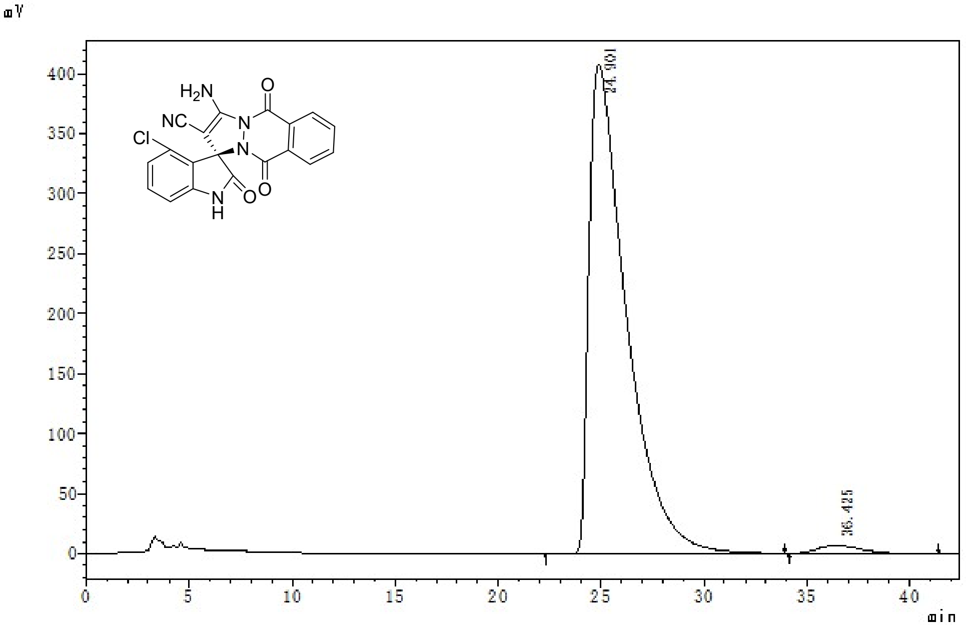

	Retention Time	Area	Height	Area%
1	24.901	51524079	407481	98.103
2	36.425	996221	6612	1.897
Total		52520300	414093	100.000

4c

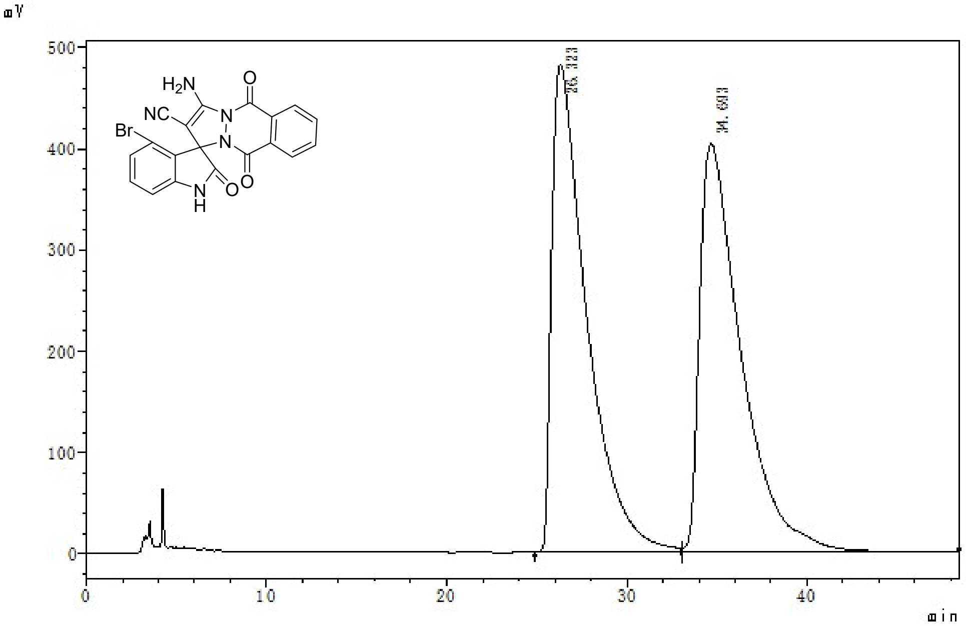

	Retention Time	Area	Height	Area%
1	26.323	65037776	481622	50.129
2	34.693	64703394	403947	49.871
Total		129741170	885568	100.000

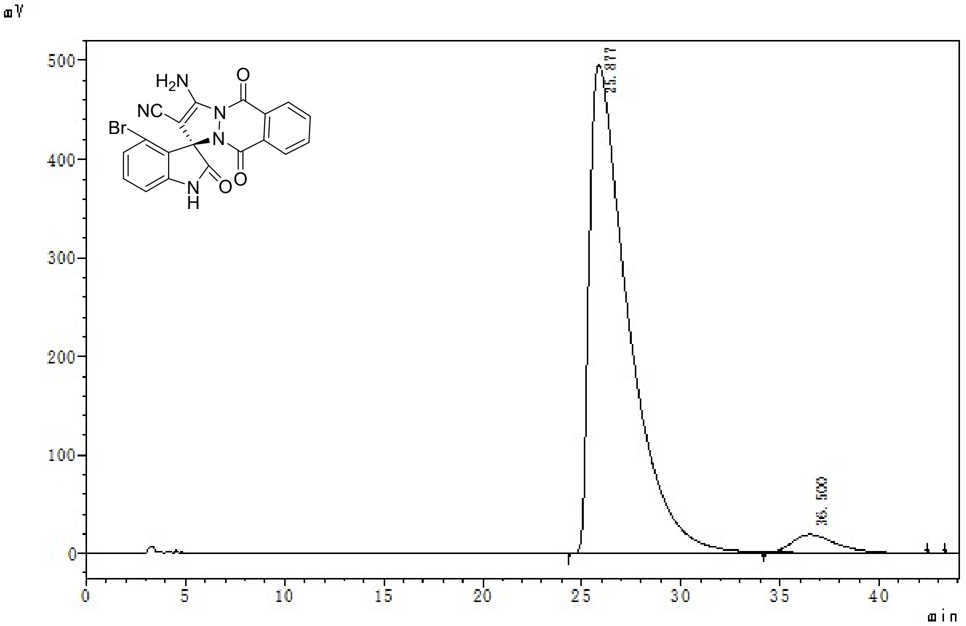

	Retention Time	Area	Height	Area%
1	25.877	68266698	495969	96.125
2	36.500	2751630	17906	3.875
Total		71018328	513875	100.000

4d

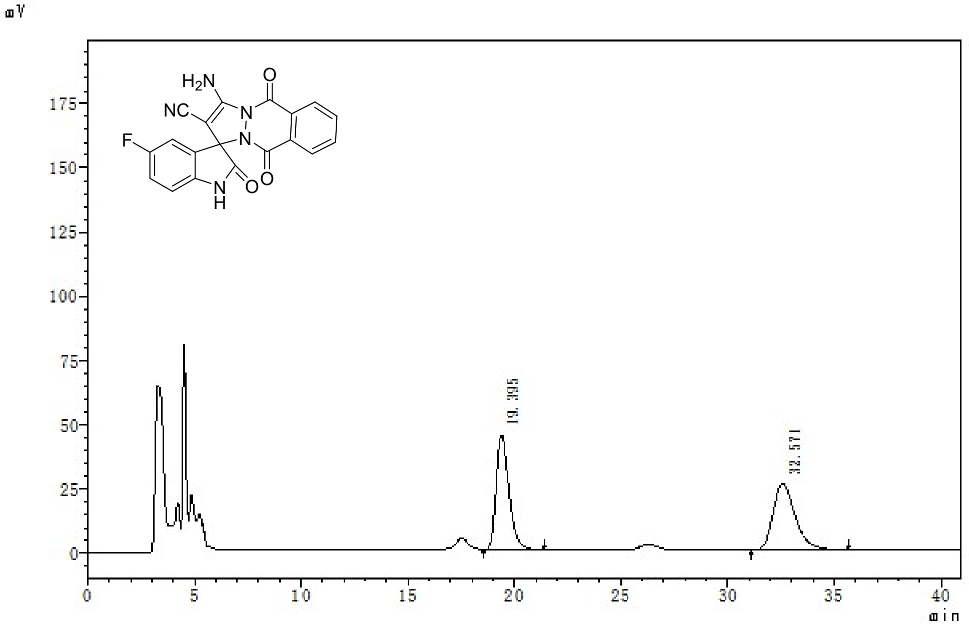

	Retention Time	Area	Height	Area%
1	19.395	1837387	44443	49.801
2	32.571	1852063	25924	50.199
Total		3689451	70367	100.000

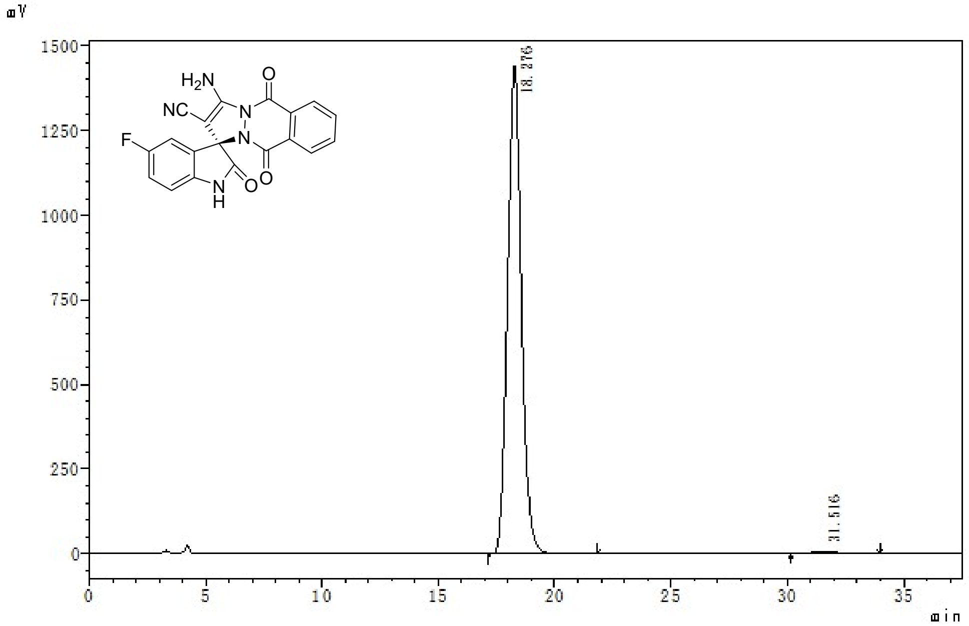

	Retention Time	Area	Height	Area%
1	18.276	59615028	1443741	98.900
2	31.516	663173	9237	1.100
Total		60278200	1452978	100.000

4e

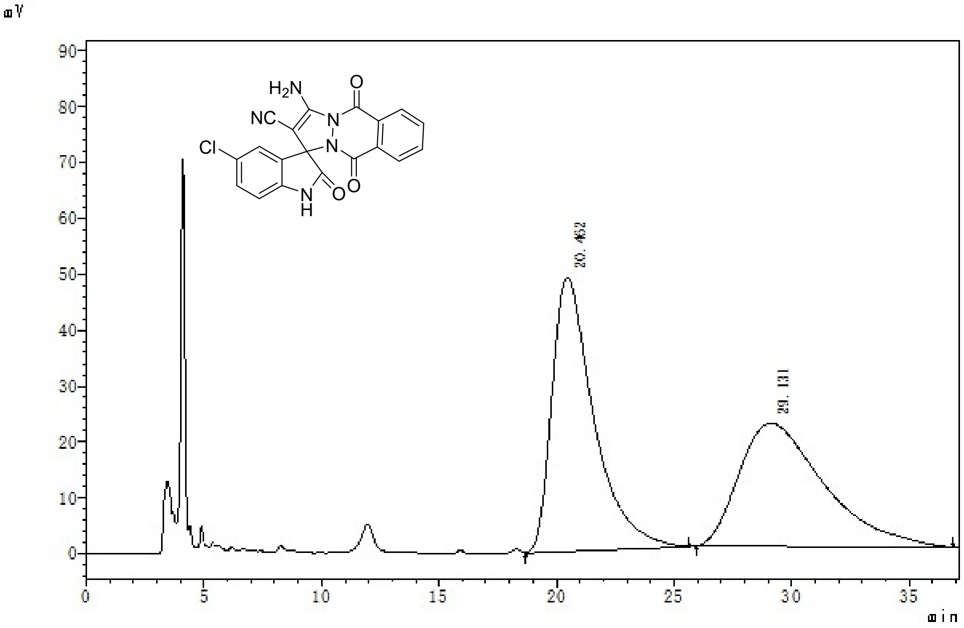

	Retention Time	Area	Height	Area%
1	20.462	6073687	49063	52.213
2	29.131	5558887	22070	47.787
Total		11632575	71133	100.000

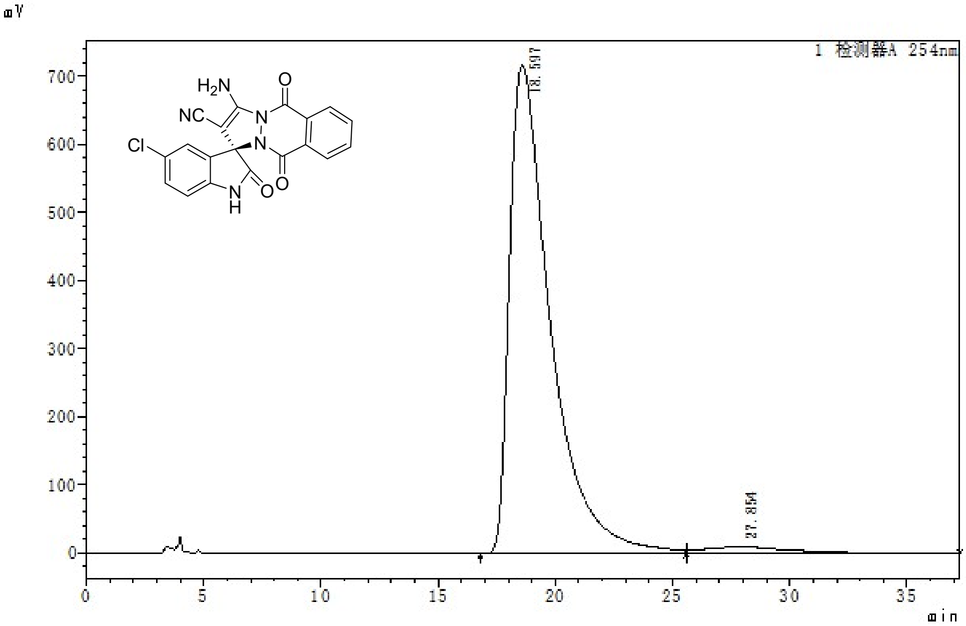

	Retention Time	Area	Height	Area%
1	18.597	87663210	718593	96.969
2	27.854	2739801	9232	3.031
Total		90403010	727825	100.000

4f

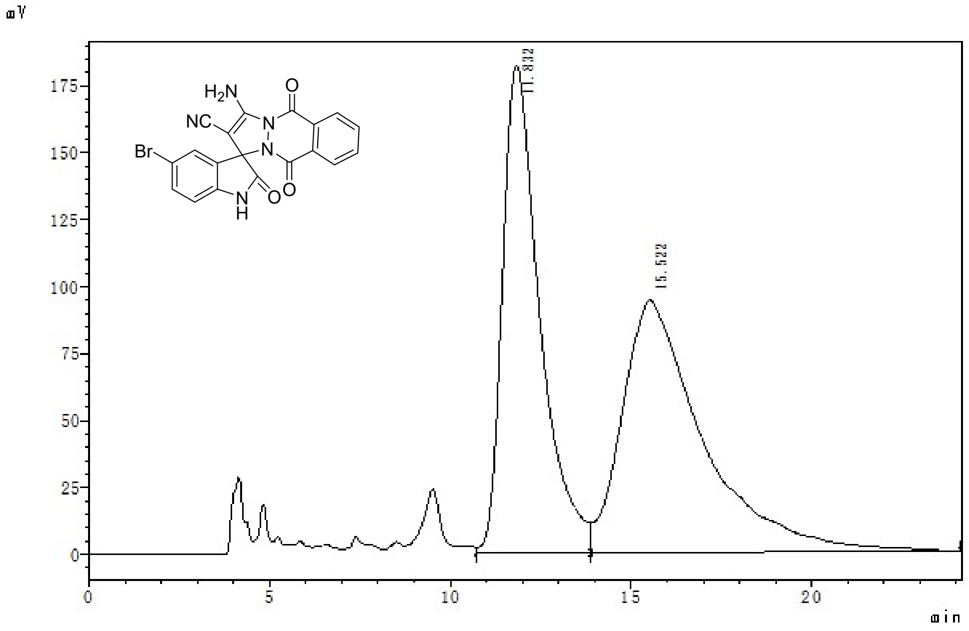

	Retention Time	Area	Height	Area%
1	11.832	13085664	181833	47.273
2	15.522	14595503	94369	52.727
Total		27681167	276202	100.000

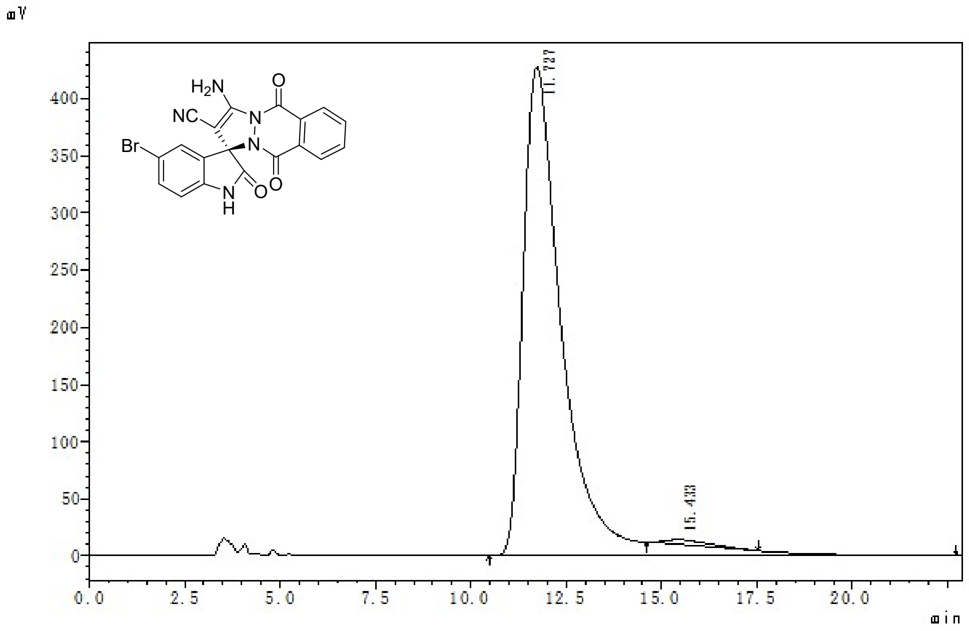

	Retention Time	Area	Height	Area%
1	11.727	31550707	427636	98.893
2	15.433	353300	3902	1.107
Total		31904007	431538	100.000

4g

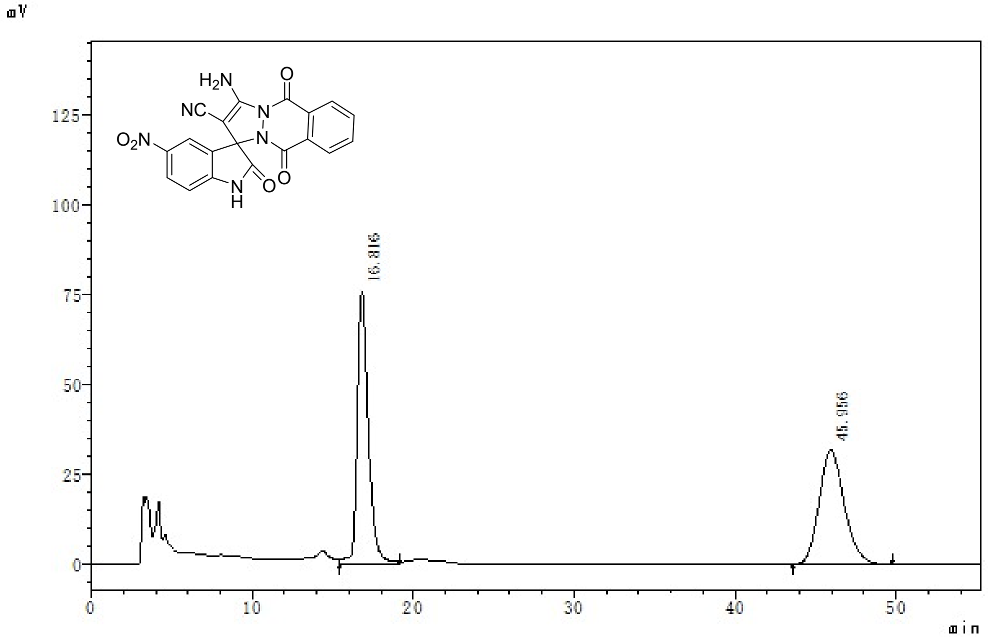

	Retention Time	Area	Height	Area%
1	16.816	3673980	76003	51.505
2	45.956	3459327	31800	48.495
Total		7133306	107803	100.000

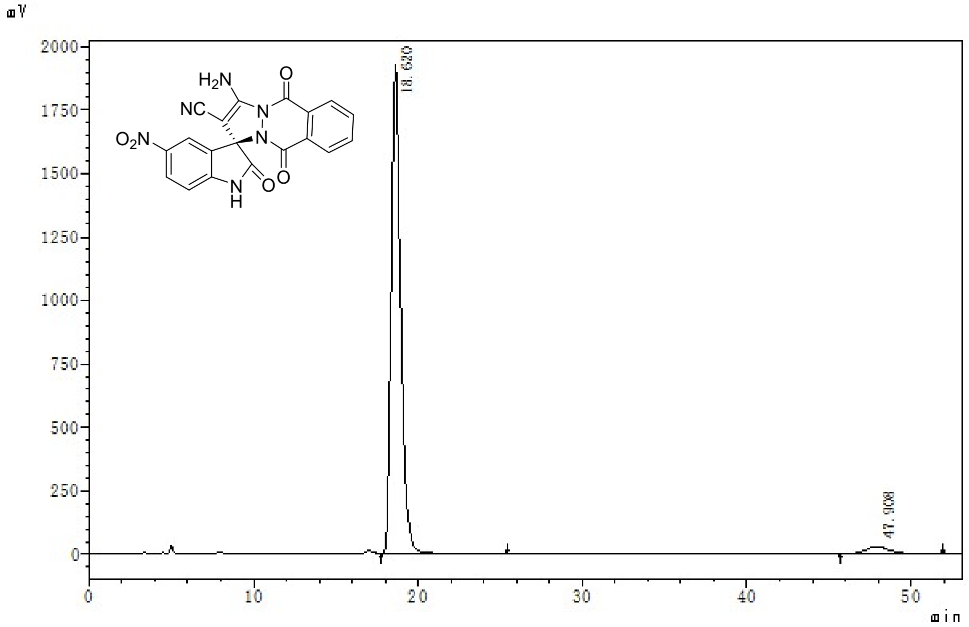

	Retention Time	Area	Height	Area%
1	18.620	77858354	1924139	96.535
2	47.908	2794925	25914	3.465
Total		80653279	1950052	100.000

4h

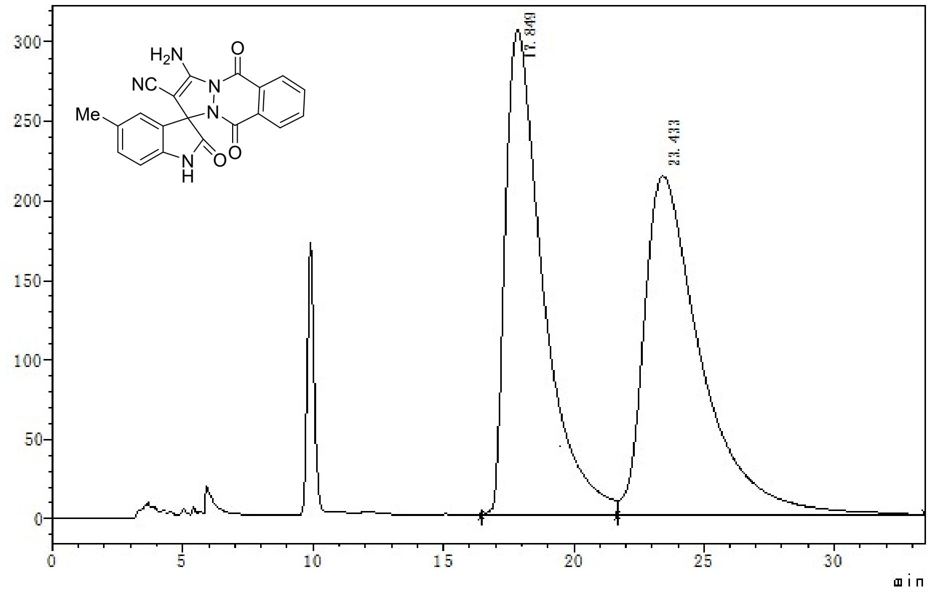

	Retention Time	Area	Height	Area%
1	17.849	31634415	305159	49.425
2	23.433	32370932	213263	50.5754
Total		64005347	518423	100.000

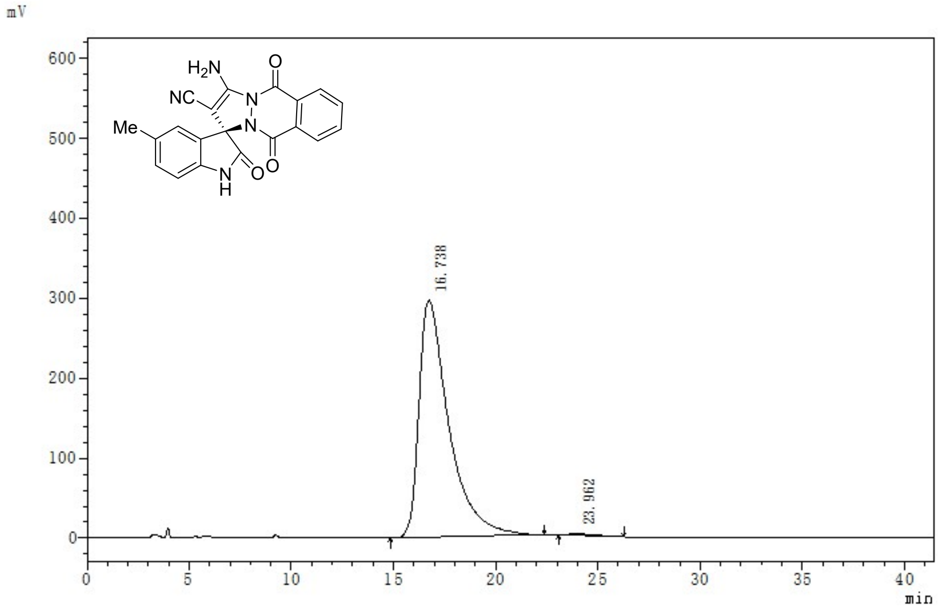

	Retention Time	Area	Height	Area%
1	16.738	30039958	296022	99.438
2	23.962	169881	2053	0.562
Total		30209839	298074	100.000

4i

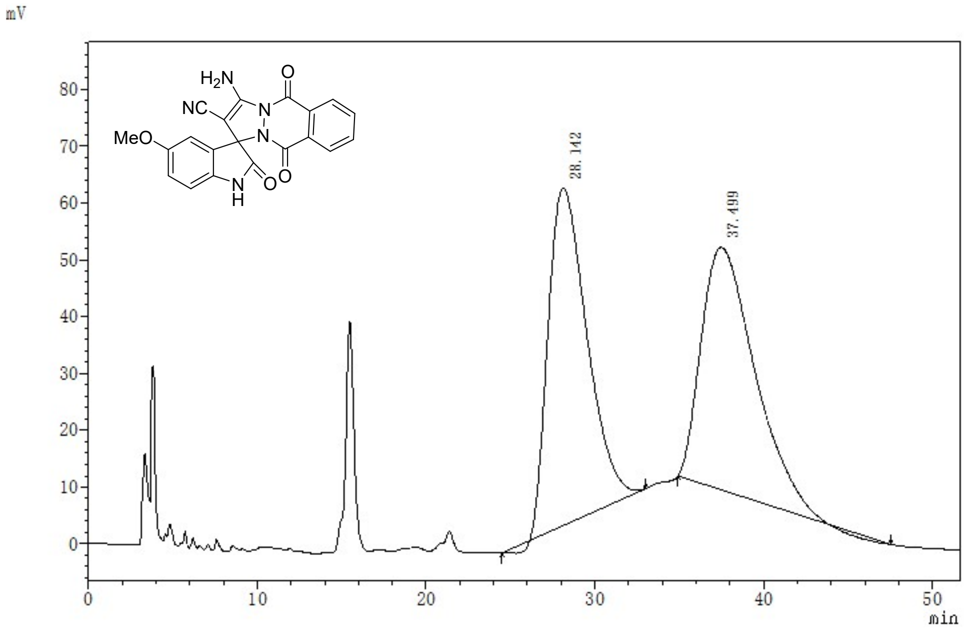

	Retention Time	Area	Height	Area%
1	28.142	9025802	58582	49.995
2	37.499	9761070	43100	50.005
Total		18786872	101682	100.000

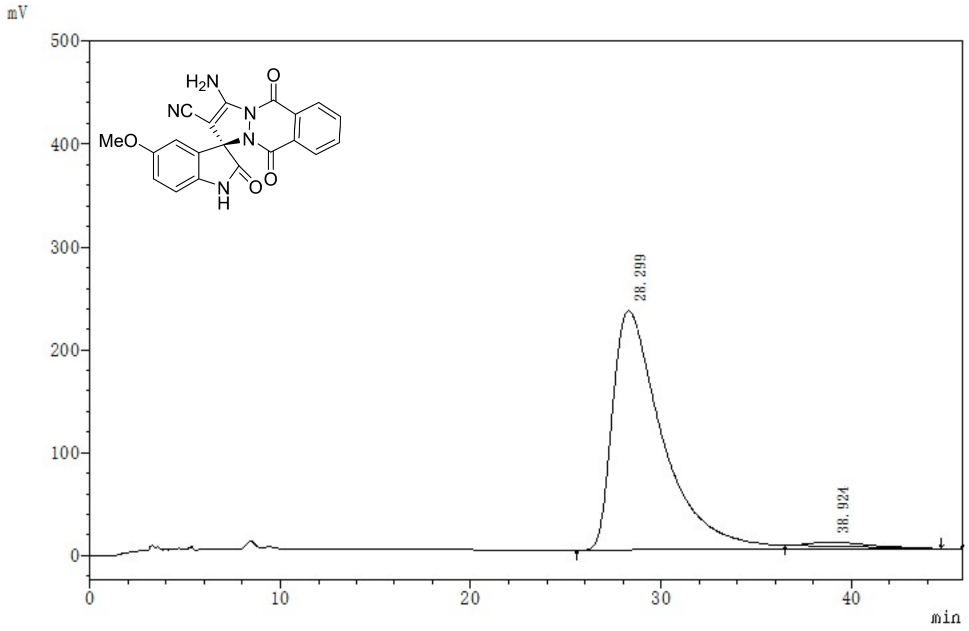

	Retention Time	Area	Height	Area%
1	28.299	43651862	232498	98.206
2	38.924	797233	3720	1.794
Total		44449095	236218	100.000

4j

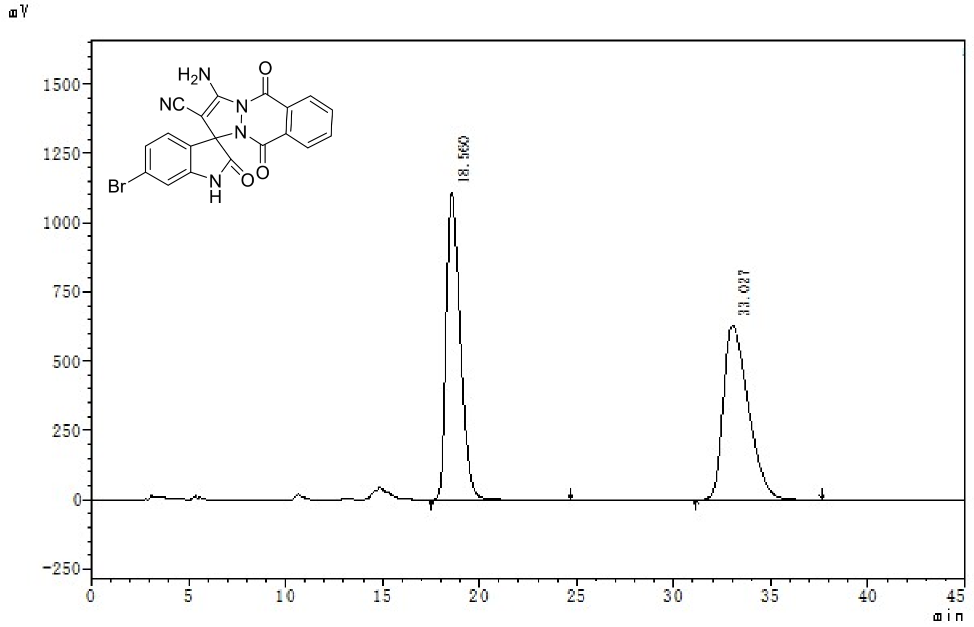

	Retention Time	Area	Height	Area%
1	18.560	55608378	1106191	49.725
2	33.027	56223154	627980	50.275
Total		111831532	1734171	100.000

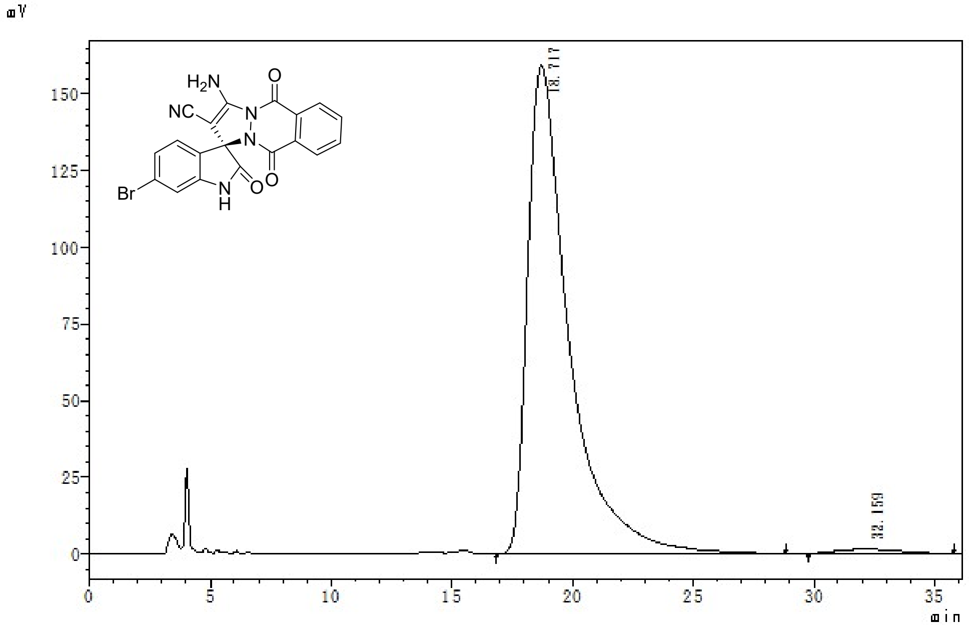

	Retention Time	Area	Height	Area%
1	18.717	19005717	159357	98.658
2	32.159	258569	1414	1.342
Total		19264286	160771	100.000

4k

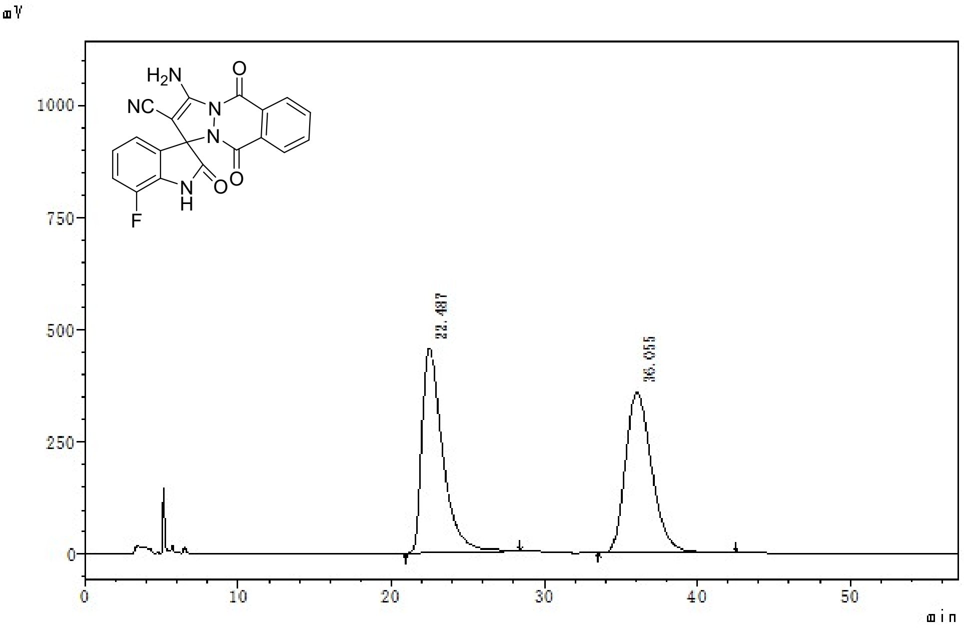

	Retention Time	Area	Height	Area%
1	22.487	42910118	454598	49.934
2	36.055	43023058	357665	50.066
Total		85933176	812263	100.000

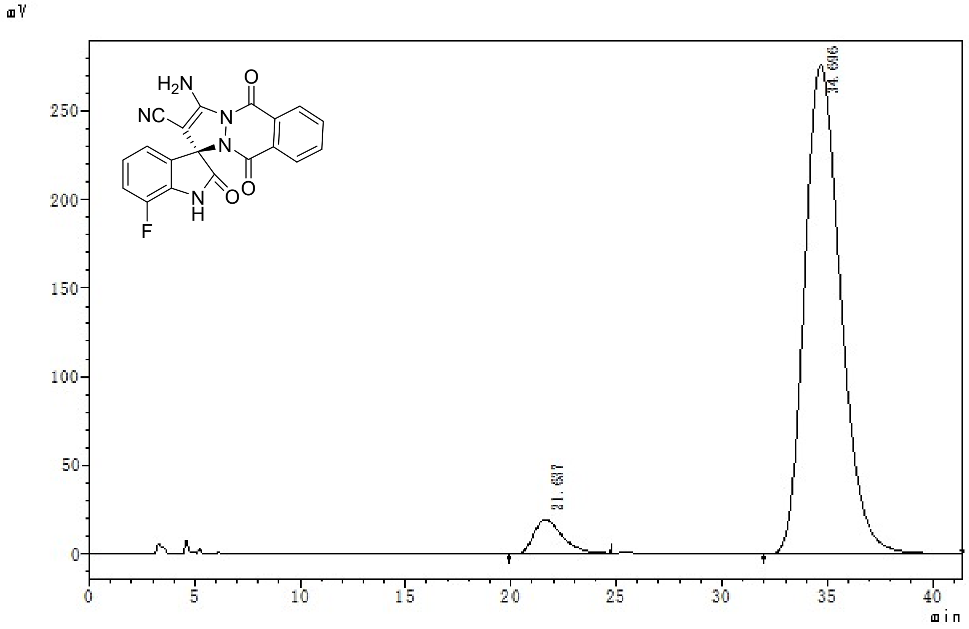

	Retention Time	Area	Height	Area%
1	21.637	1829222	19489	5.270
2	34.696	32880095	275956	94.730
Total		34709317	295446	100.000

4l

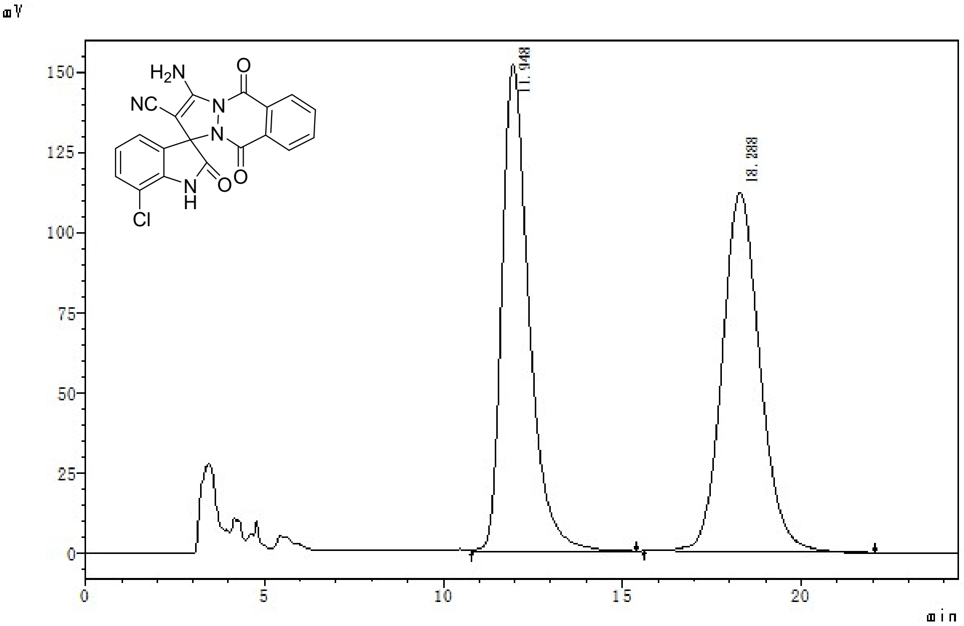

	Retention Time	Area	Height	Area%
1	11.948	7994911	151559	49.413
2	18.288	8184800	111755	50.587
Total		16179711	263313	100.000

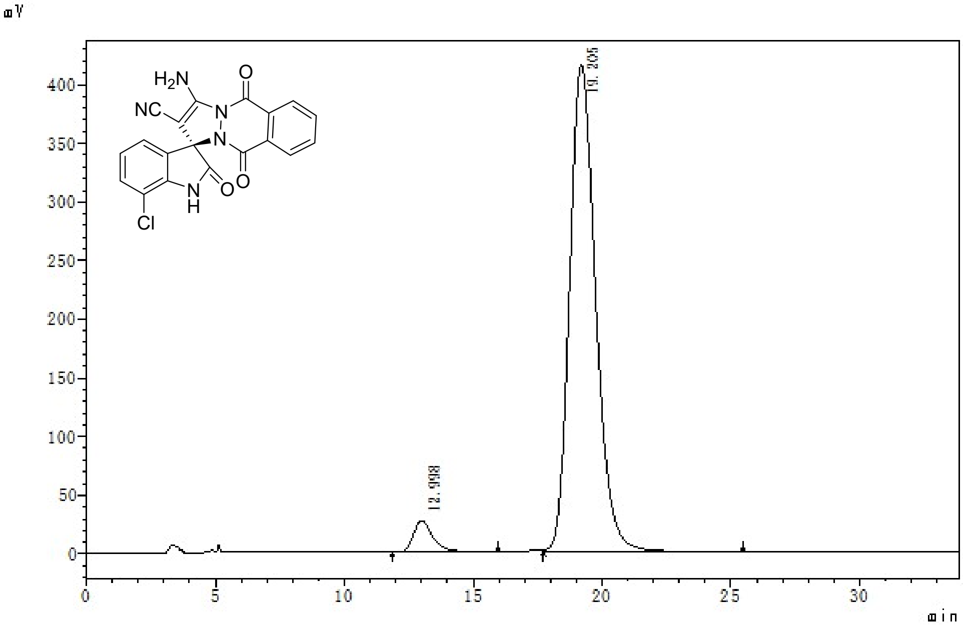

	Retention Time	Area	Height	Area%
1	12.998	1415389	26801	4.601
2	19.205	29344695	415198	95.399
Total		30760084	441999	100.000

4m

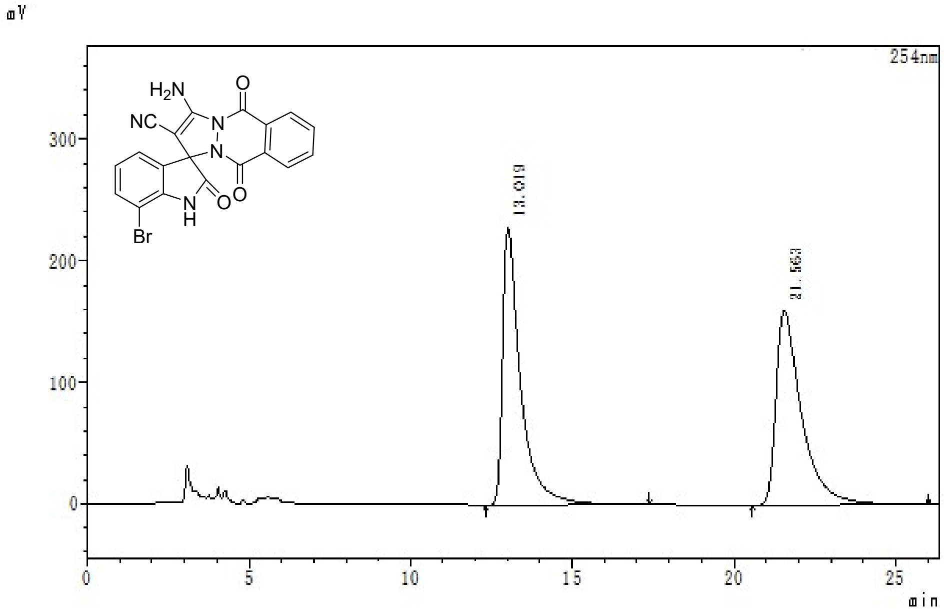

	Retention Time	Area	Height	Area%
1	13.019	8862836	227644	50.070
2	21.563	8837921	159634	49.930
Total		17700757	387278	100.000

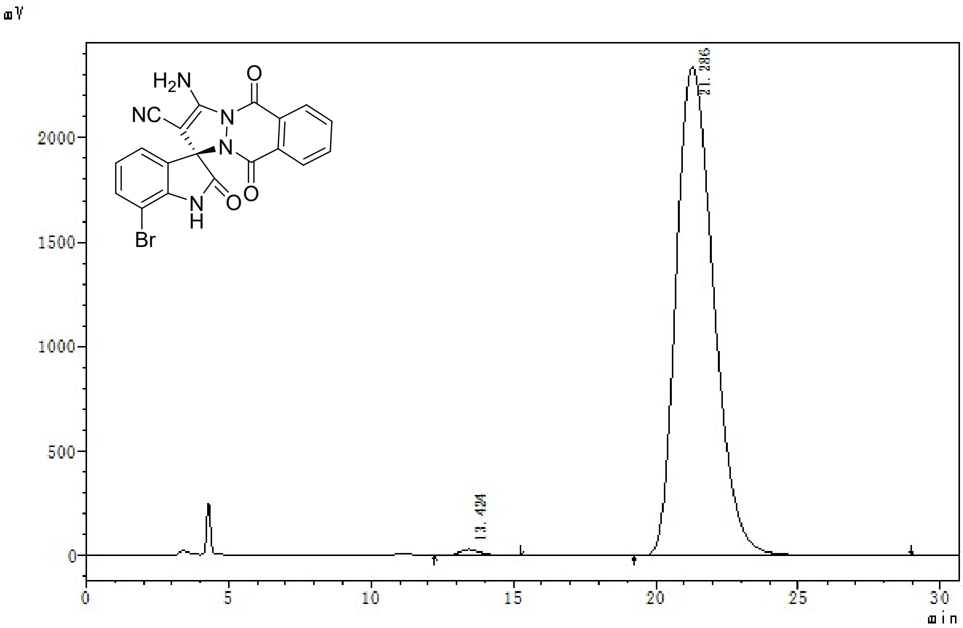

	Retention Time	Area	Height	Area%
1	13.424	1682027	28708	0.782
2	21.286	213369069	2337987	99.218
Total		215051096	2366695	100.000

4n

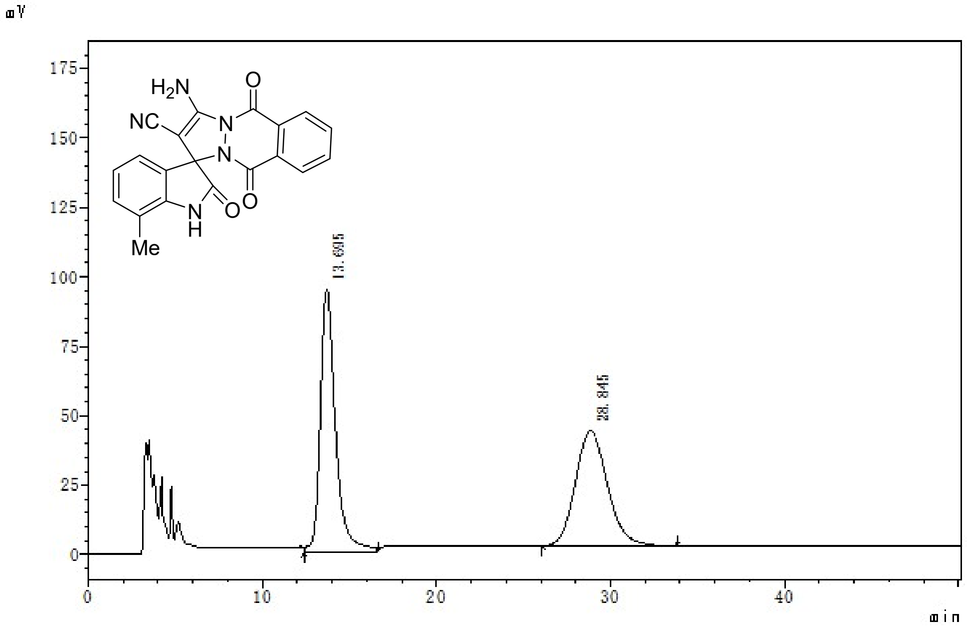

	Retention Time	Area	Height	Area%
1	13.695	5782309	94664	52.336
2	28.845	5266161	41227	47.664
Total		11048470	135890	100.000

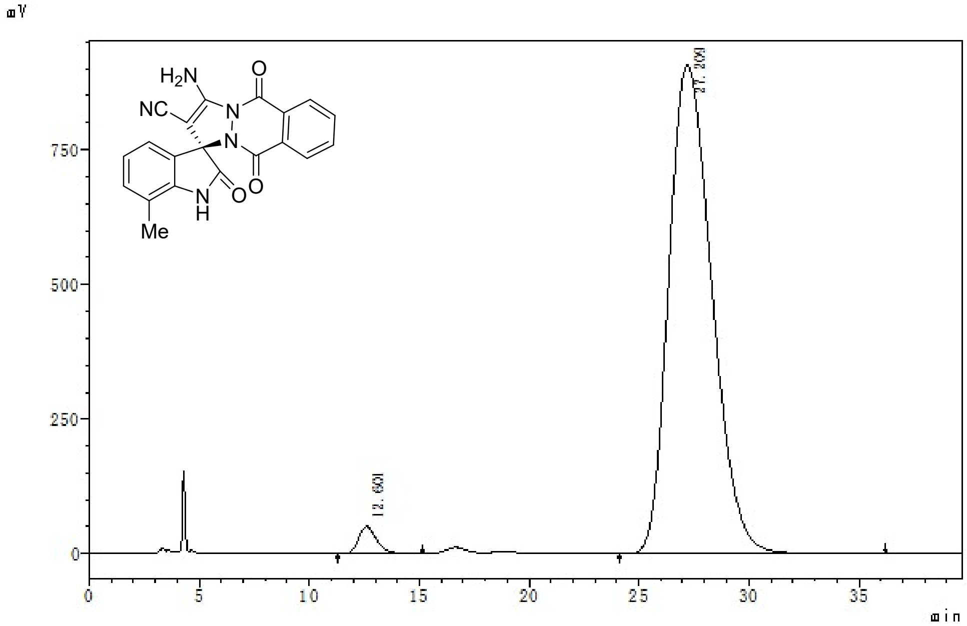

	Retention Time	Area	Height	Area%
1	12.601	2996156	50645	2.369
2	27.209	123493803	907200	97.631
Total		126489959	957845	100.000

4o

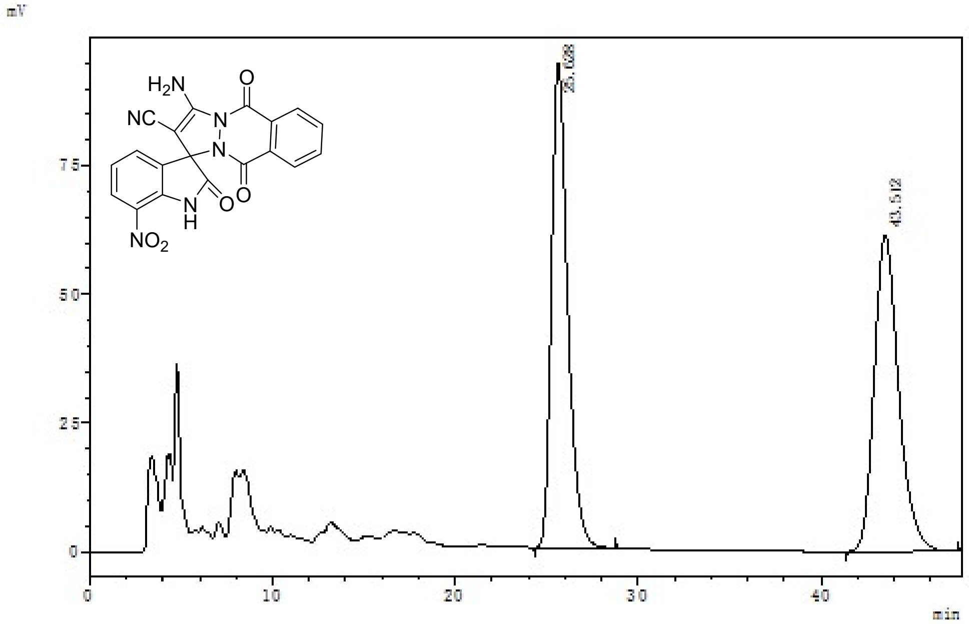

	Retention Time	Area	Height	Area%
1	25.628	5819463	94213	50.497
2	43.512	5704890	61444	49.503
Total		11524353	155658	100.000

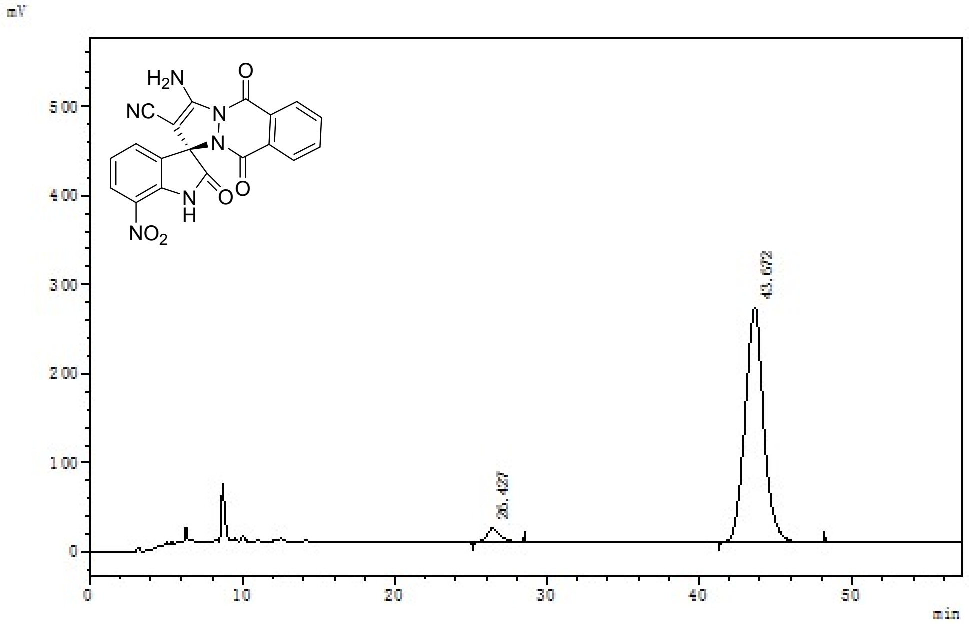

	Retention Time	Area	Height	Area%
1	26.427	854135	14776	3.669
2	43.672	22428415	262835	96.331
Total		23282550	277611	100.000

4p

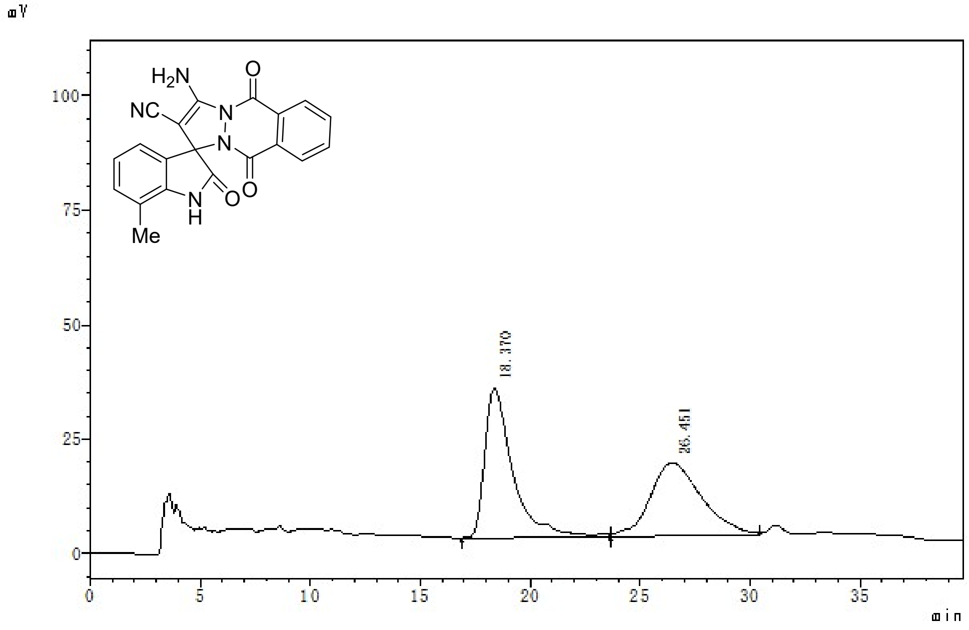

	Retention Time	Area	Height	Area%
1	18.370	2911397	32616	52.759
2	26.451	2606947	15928	47.241
Total		5518343	48544	100.000

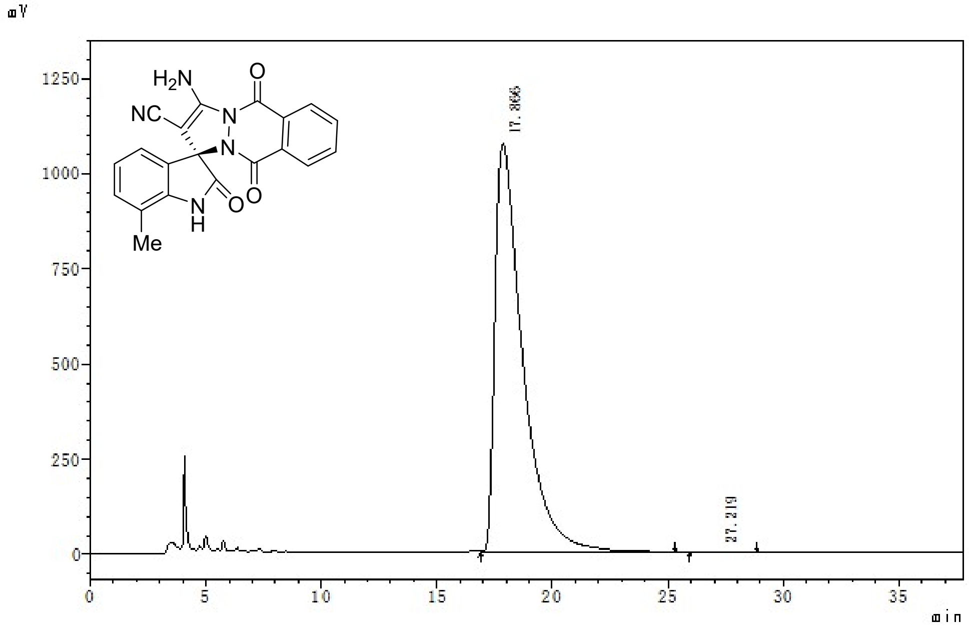

	Retention Time	Area	Height	Area%
1	17.866	90064463	1073639	99.861
2	27.219	125317	1236	0.139
Total		90189779	1074875	100.000

4q

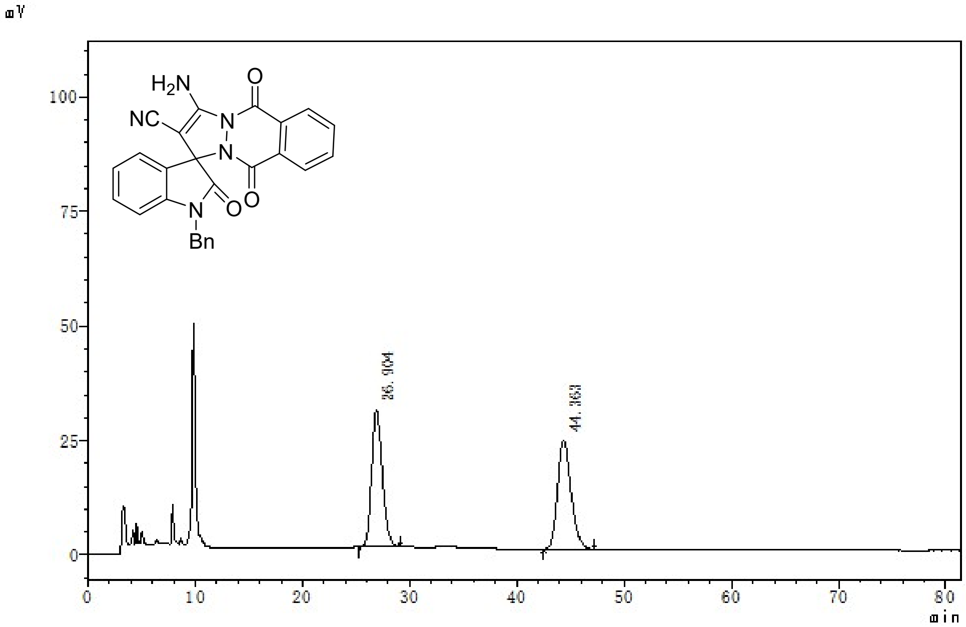

	Retention Time	Area	Height	Area%
1	26.904	2062452	30111	49.770
2	44.363	2081520	23812	50.230
Total		4143973	53922	100.000

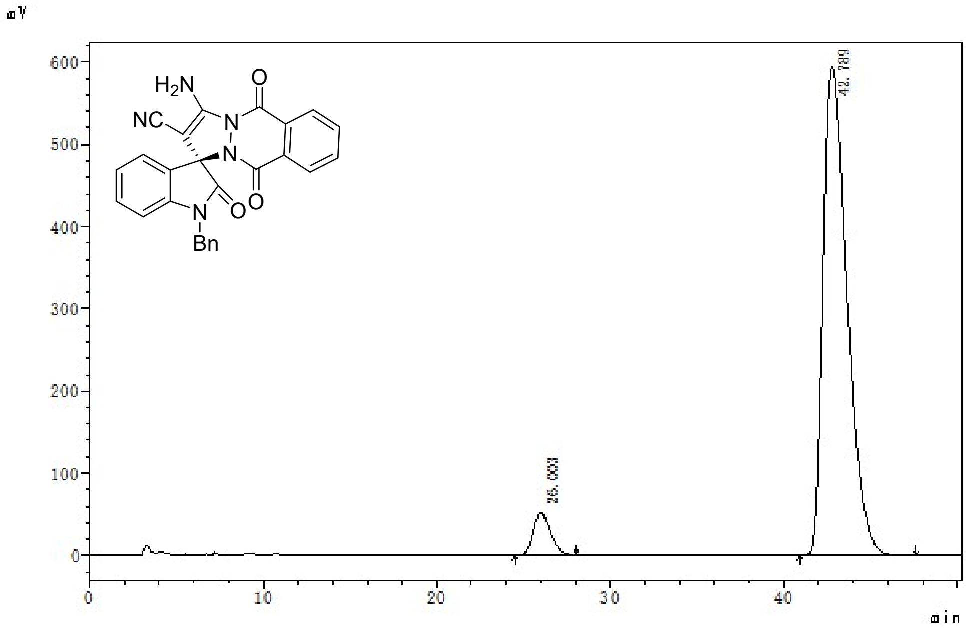

	Retention Time	Area	Height	Area%
1	26.003	3578125	51887	5.865
2	42.789	57427196	594619	94.135
Total		61005320	646506	100.000

4r

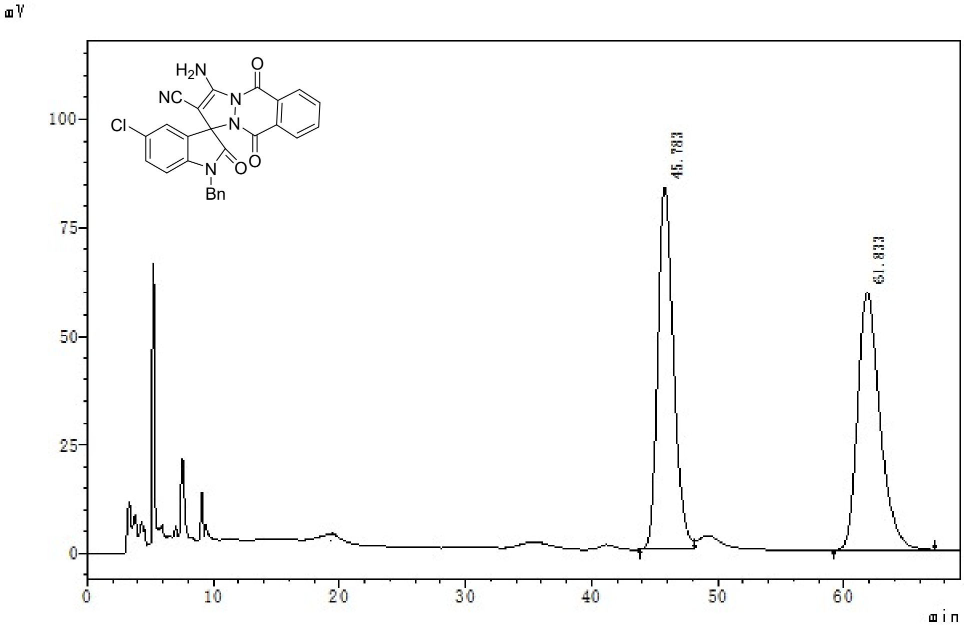

	Retention Time	Area	Height	Area%
1	45.783	7249101	83141	49.840
2	61.833	7295604	59309	50.160
Total		14544706	142450	100.000

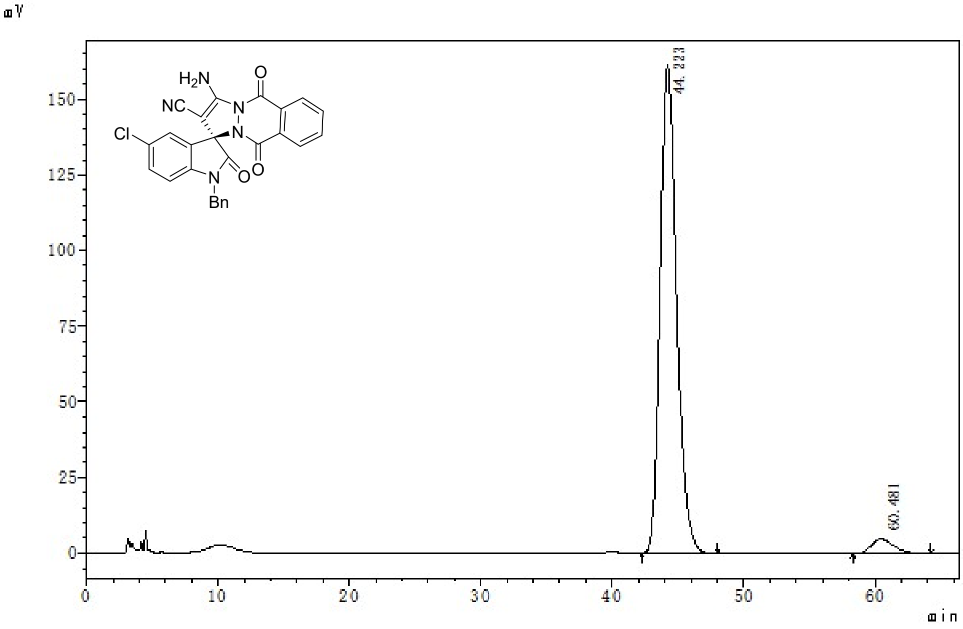

	Retention Time	Area	Height	Area%
1	44.223	14019639	161382	95.907
2	60.481	598275	4901	4.093
Total		14617914	166283	100.000

4s

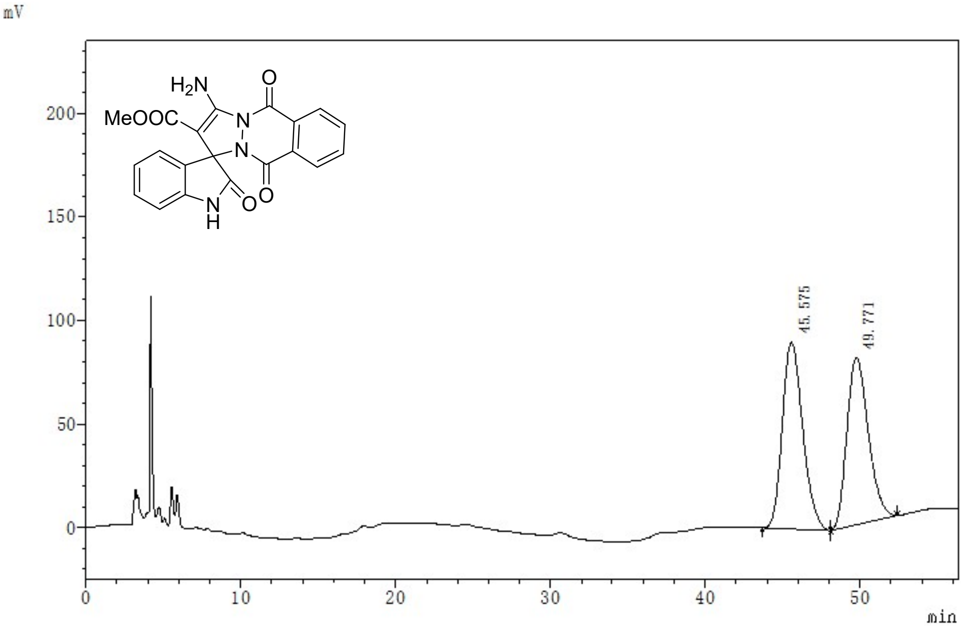

	Retention Time	Area	Height	Area%
1	45.575	8262056	90078	51.656
2	49.771	7732317	80583	48.344
Total		15994373	170661	100.000

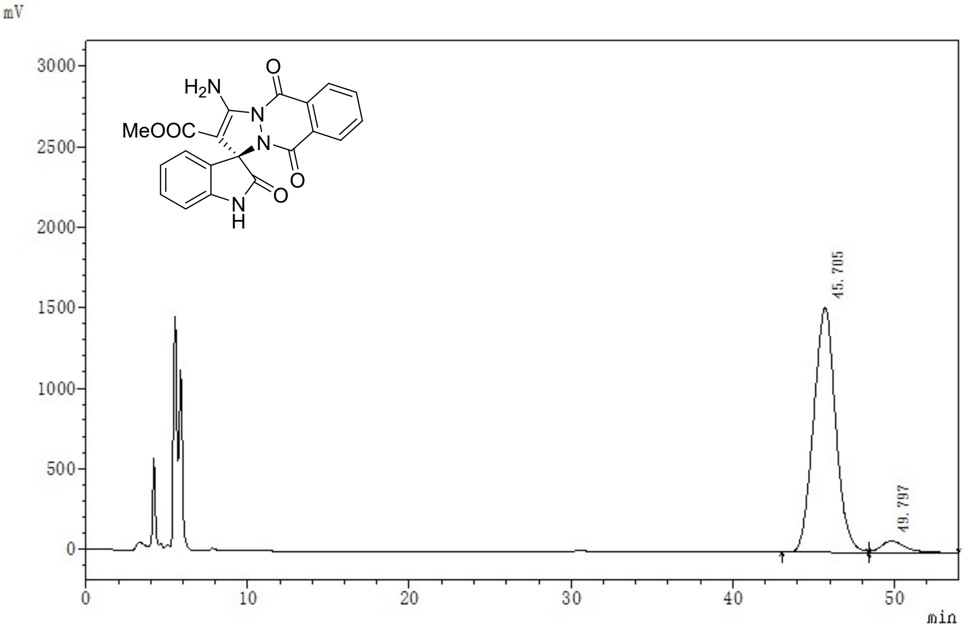

	Retention Time	Area	Height	Area%
1	45.705	143921098	1520289	95.314
2	49.797	7075651	70456	4.686
Total		150996749	1590745	100.000

4t

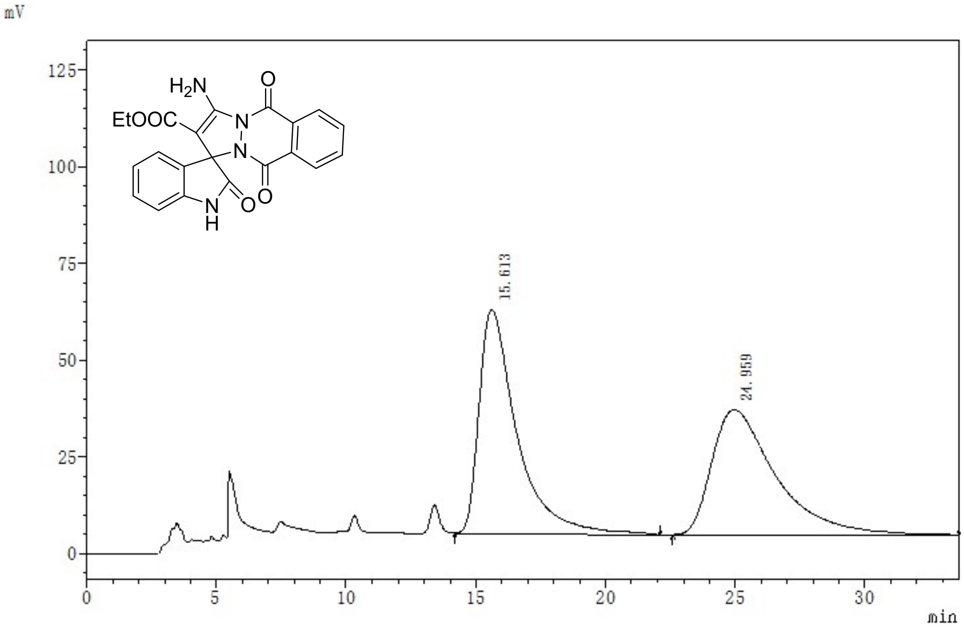

	Retention Time	Area	Height	Area%
1	15.613	5907935	57942	51.470
2	24.959	5570569	32348	48.530
Total		11478504	90289	100.000

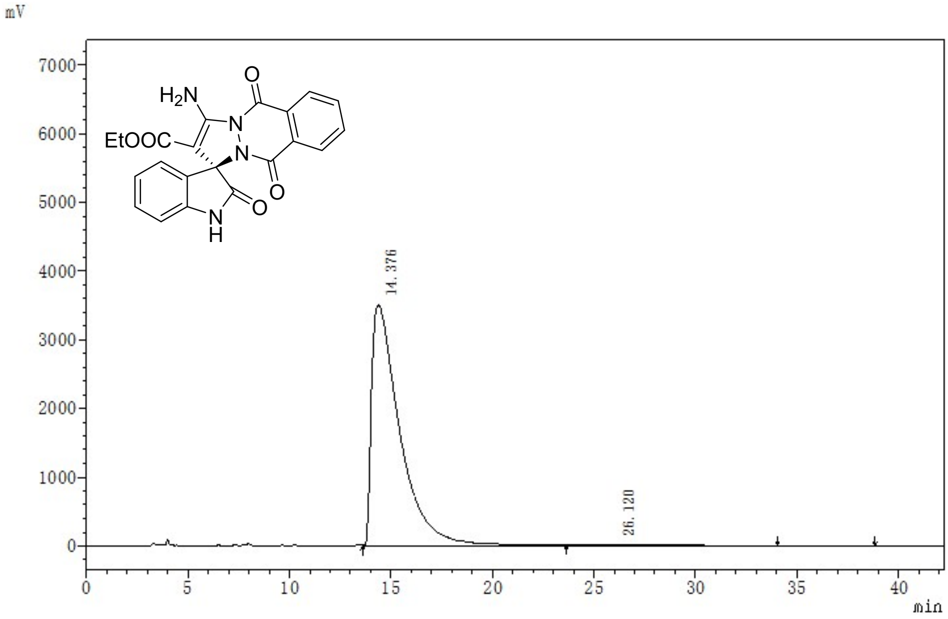

	Retention Time	Area	Height	Area%
1	14.376	343528384	3505480	98.996
2	26.120	3483001	14149	1.004
Total		347011385	3519629	100.000

4u

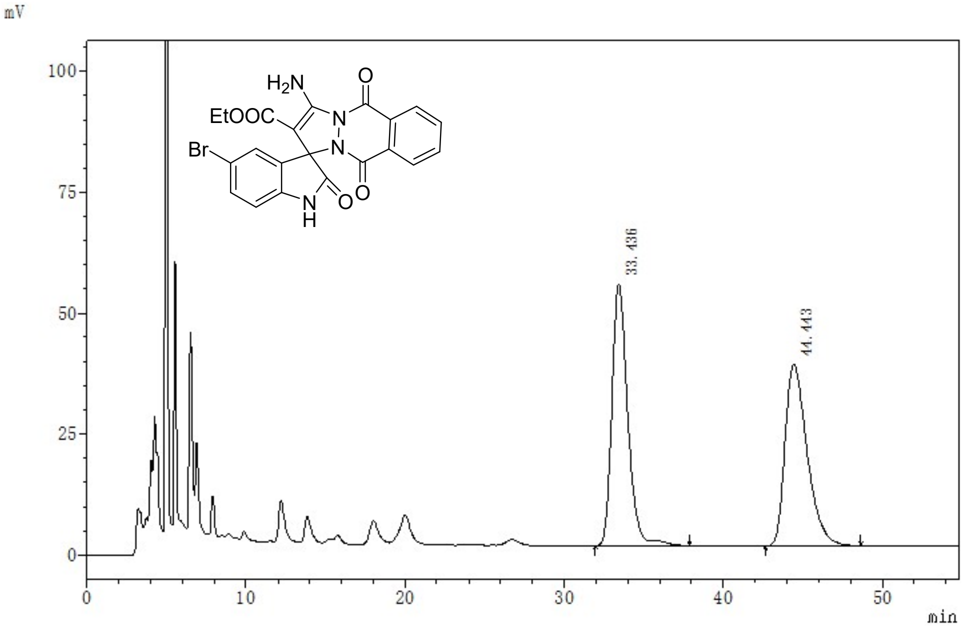

	Retention Time	Area	Height	Area%
1	33.436	3721498	54074	50.635
2	44.443	3628175	37550	49.365
Total		7349673	91625	100.000

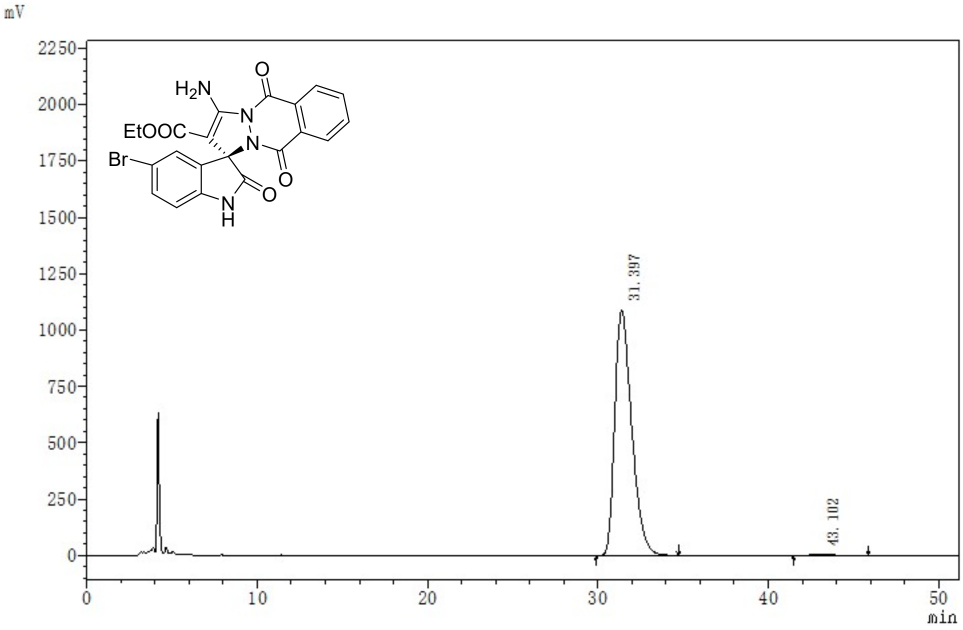

	Retention Time	Area	Height	Area%
1	31.397	75069461	1086561	99.563
2	43.102	329181	3415	0.437
Total		75398642	1089976	100.000

4v

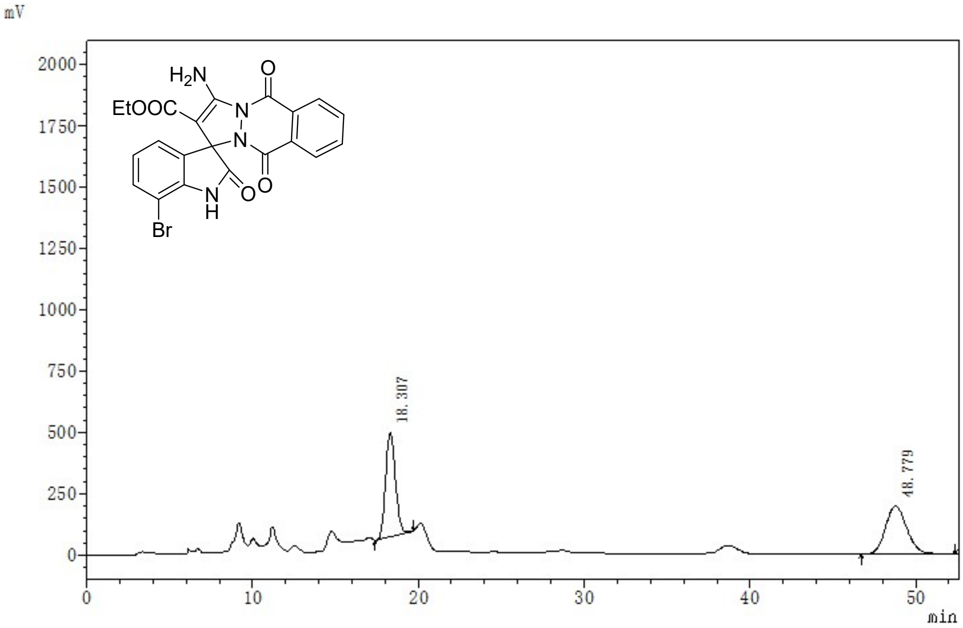

	Retention Time	Area	Height	Area%
1	18.307	17477173	424233	49.680
2	48.779	17702304	197137	50.320
Total		35179477	621370	100.000

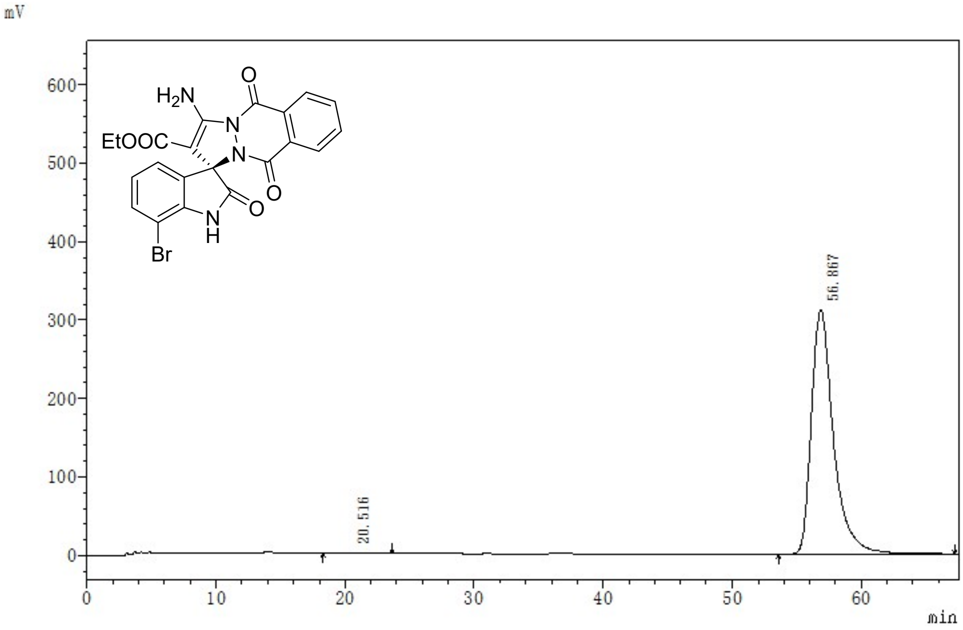

	Retention Time	Area	Height	Area%
1	20.516	203559	1096	0.544
2	56.867	37245728	310737	99.456
Total		37449287	311833	100.000

4w

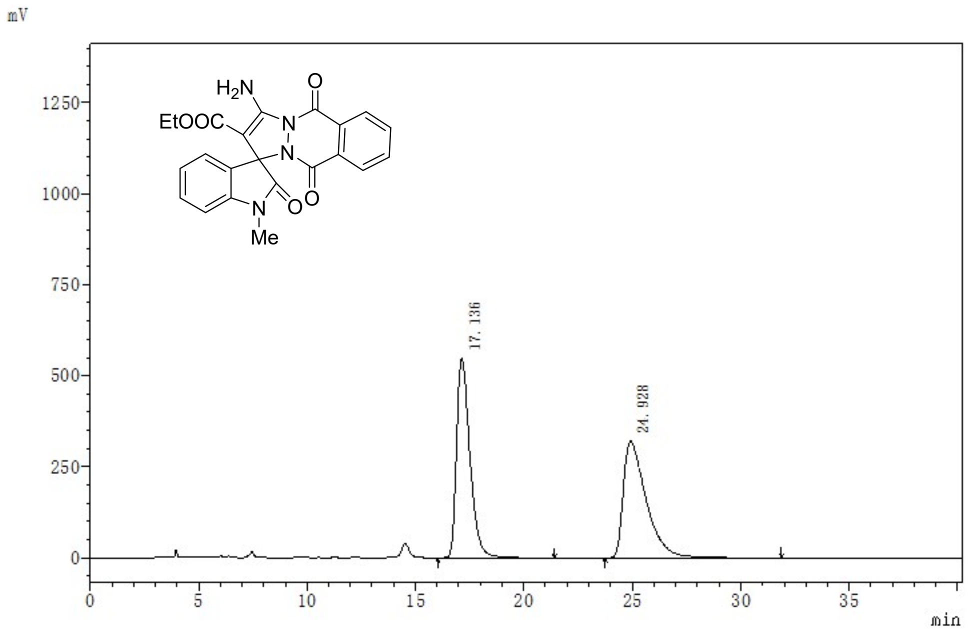

	Retention Time	Area	Height	Area%
1	17.136	23464291	546458	49.866
2	24.928	23590743	320518	50.134
Total		47055034	866976	100.000

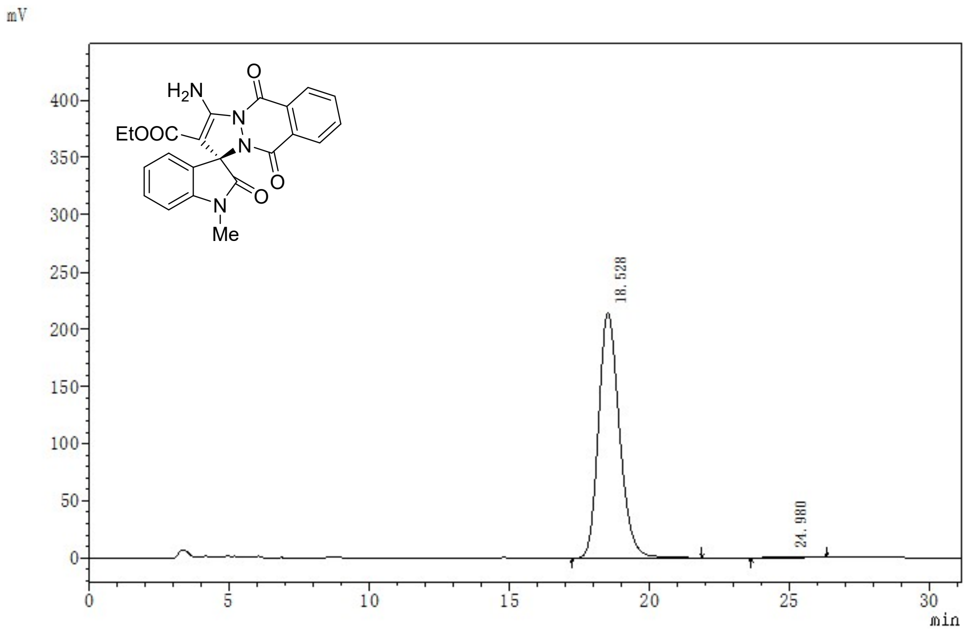

	Retention Time	Area	Height	Area%
1	18.528	11070710	213913	99.538
2	24.980	51393	700	0.462
Total		11122103	214613	100.000

5. X-Ray crystal data of compound 4d

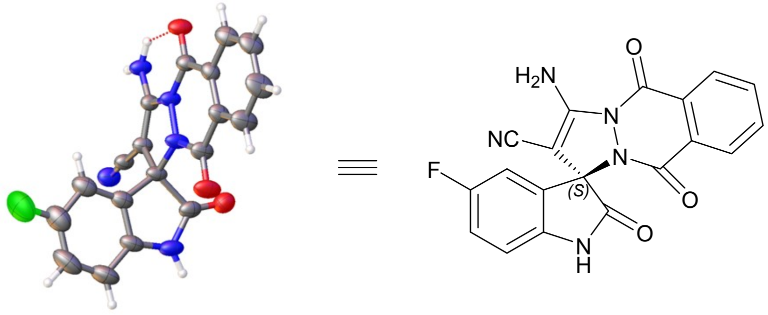

CCDC:2384066

Table S1 Crystal data and structure refinement for 4d.

Identification code	**4d**
Empirical formula	$C_{19.92}H_{11.83}Cl_{1.83}FN_5O_3$
Formula weight	453.20
Temperature/K	173.00
Crystal system	orthorhombic
Space group	$P2_12_12_1$
a/Å	16.7979(6)
b/Å	22.4214(9)
c/Å	35.2924(11)
α/°	90
β/°	90
γ/°	90
Volume/Å^3	13292.3(8)
Z	24
ρ_{calc}g/cm^3	1.359
μ/mm^{-1}	2.802
F(000)	5532.0
Crystal size/mm^3	0.13 × 0.12 × 0.08
Radiation	CuKα (λ = 1.54178)
2Θ range for data collection/°	6.374 to 127.538
Index ranges	$-16 \leq h \leq 19, -22 \leq k \leq 25, -40 \leq l \leq 36$
Reflections collected	51175
Independent reflections	20901 [R_{int} = 0.0419, R_{sigma} = 0.0553]
Data/restraints/parameters	20901/477/1927
Goodness-of-fit on F^2	1.045
Final R indexes [I>=2σ (I)]	R_1 = 0.0649, wR_2 = 0.1716
Final R indexes [all data]	R_1 = 0.0725, wR_2 = 0.1803
Largest diff. peak/hole / e Å^{-3}	0.86/-0.67
Flack parameter	0.05(3)
